# Transforming Silver Nanoclusters from Racemic to Homochiral via Seeded Crystallization

**DOI:** 10.1021/acs.jpclett.3c00794

**Published:** 2023-05-26

**Authors:** Along Ma, Wenjun Du, Jiawei Wang, Kefan Jiang, Cheng Zhang, Wenhan Sheng, Haiyan Zheng, Rongchao Jin, Shuxin Wang

**Affiliations:** † College of Materials Science and Engineering, Qingdao University of Science and Technology, Qingdao, Shandong 266042, P. R. China; ‡ Department of Chemistry, Carnegie Mellon University, Pittsburgh, Pennsylvania 15213, United States; § Anhui Provincial Key Laboratory for Degradation and Monitoring of Pollution of the Environment, School of Chemistry and Materials Engineering, Fuyang Normal University, Fuyang 236037, P. R. China

## Abstract

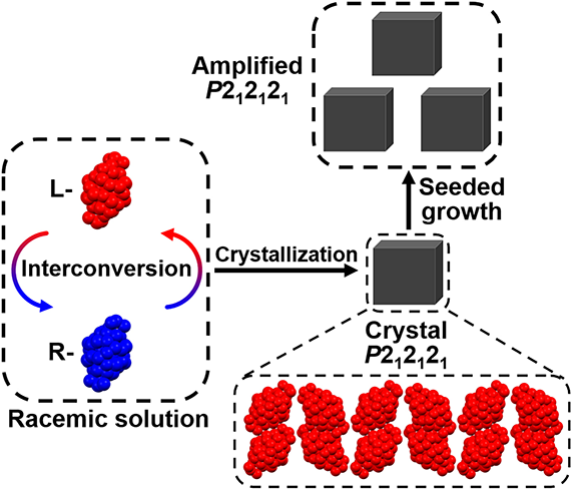

Chirality has risen as an attractive topic in materials research in recent years, but the attainment of enantiopure materials remains a major challenge. Herein, we obtained homochiral nanoclusters by a recrystallization strategy, without any chiral factors (i.e., chiral ligands, counterions, etc.). Through the rapid flipping of configuration of silver nanoclusters in solution, the initial racemic **Ag**_**40**_ (triclinic) nanoclusters are converted to homochiral (orthorhombic) as revealed by X-ray crystallography. In the seeded crystallization, one homochiral **Ag**_**40**_ crystal is used as a seed to direct the growth of crystals with specific chirality. Furthermore, enantiopure **Ag**_**40**_ nanoclusters can be used as amplifiers for the detection of chiral carboxylic drugs. This work not only provides chiral conversion and amplification strategies to obtain homochiral nanoclusters but also explains the chirality origin of nanoclusters at the molecular level.

In nature, chirality is a common feature in numerous systems, ranging from small molecules (e.g., tartaric acid and amino acids) to atomic aggregates (e.g., nanoparticles and nanoclusters) and macroscopic objects (e.g., human hands and atmospheric cyclones). In recent years, great progress has been made in the application of chiral nanomaterials in asymmetric catalysis, biorecognition, chiral medicine, and chiroptics.^1−5^ However, the origin of chirality generation is still unclear. Further, achieving symmetry breaking and amplification of chiral products are challenging. On the one hand, absolute asymmetric synthesis (AAS) was attained; that is, in the absence of chiral ligands or chiral environments, an enantiomer is spontaneously obtained.^6,7^ It is worth noting that external forces are usually required to achieve symmetry breaking, such as stirring, ultrasound, temperature gradients, and so on.^8−11^ Without the presence of such factors, the enantiomeric excess (ee) value of the obtained product will be significantly lower, and even racemic results will be obtained. This motivated us to ponder whether the molecule itself can complete the symmetry breaking and give rise to a product with homochirality in the absence of external forces. On the other hand, the seeded crystallization method has been widely used in the synthesis of nanocrystals.^12,13^ Furthermore, seed crystals can affect the morphology and properties of crystal products.^12,13^ For example, NaClO_3_ is achiral in solution, but it can crystallize into chiral crystals, and chiral seed crystals of NaClO_3_ can induce the formation of chiral crystal products.^13^

Ligand-protected nanoparticles with atomically precise nature, often called nanoclusters (NCs), have been of tremendous interest due to their precise structures and extraordinary properties.^14−32^ By introducing chiral factors, such as chiral ligands or chiral counterions, optically active nanoclusters have been synthesized.^33−40^ In addition, racemic nanoclusters such as Au_38_ were separated into enantiomers through chiral resolution.^41−45^ Further studies have shown that it is difficult to make homogold nanoclusters undergo a configuration reversal at room temperature. However, it is interesting that, after doping silver atoms into the kernel of gold nanoclusters, the temperature for configuration reversal can be significantly reduced.^46,47^ Theoretically, thiolated silver or heavily silver-doped nanoclusters can only exist in the form of racemates in solution.^47−49^ The rapid flip of chirality is achieved by changing the arrangement of the outer metal complex shell. Since the nanocluster itself has a metal core, its chiral construction does not depend on primary nucleation as in the AAS process. In theory, external factors (such as stirring, ultrasound, etc.) are not necessary to achieve symmetry breaking. For the cluster protected by the achiral coligands and crystallized in a noncentrosymmetric space group, if one can build a stable system and obtain only one crystal, then nearly 100% ee value could be obtained.^50^ In the seeded crystallization method, the chirality of seed crystals can affect the chirality of final products. The racemic nanoclusters can rapidly flip between chiral structures in solution. If a chiral crystal is used as a seed, it may be feasible for racemic clusters to grow into homochiral crystals with the initial seed; then amplification of chiral crystals will be realized.

In this work, we devise a crystallization approach for achieving homochiral recrystallization and seeded crystallization. We first synthesized racemic **Ag**_**40**_ nanoclusters. Then, we developed an automatic crystallization system by using a robot in order to quickly screen the crystallization conditions. After optimizing the conditions, the probability of obtaining one crystal in the crystallization vial is close to 10%. After testing more than 40 crystals, we found that 2 of the crystals are in the orthorhombic system (*P*2_1_2_1_2_1_, flack = 0.006), which means that an enantiomeric excess close to 100% has been achieved, and the chirality of the resulting crystal is random in our current work without introducing any chiral factors and external forces. Further, based on the principle of rapid structure flipping of silver nanoclusters in solution, using one homochiral crystal obtained by recrystallization as the seed allows rapid construction of crystals with conspecific chirality in a racemic solution. The chirality of the final product crystals is consistent with the chirality of the initial seed. It should be noted that the state of the resulting products is many crystals and not a single one. In addition, **Ag**_**40**_ nanoclusters can be used as chiral amplifiers to determine the ee values of chiral carboxylic acids. This work not only provides new insight into the chiral origin of nanomaterials but also provides new insight for chiral separation and chiral crystal amplification.

Racemic **Ag**_**40**_ was first synthesized, and crystals were obtained (Figure S1a). Details of the synthesis are provided in the Supporting Information. X-ray crystallography revealed that racemic **Ag**_**40**_ NCs were crystallized in a triclinic *P*1̅ space group, which is achiral. The crystal structure of **Ag**_**40**_ can be viewed as a kernel-shell structure, which contains an icosahedral Ag_13_ kernel and an Ag_27_ complex shell (Figure S2). Notably, the Ag_27_ complex shell consists of three Ag_7_ chains, every two Ag_7_ chains are connected by an Ag_2_(CH_3_COO)(TBBM)_4_ “button”, and the chiral structure is determined by the direction of the chain rotation (Figures 1 and S3). The structure of **Ag**_**40**_ obtained in this work is not the same as the **Ag**_**40**_ previously reported;^51^ however, it is similar to the doped AuAg_39_ nanocluster.^51^ The UV–vis absorption spectrum of **Ag**_**40**_ in CH_2_Cl_2_ (DCM) exhibits three characteristic peaks at 460, 500, and 610 nm (Figure S4a), the corresponding circular dichroism (CD) spectrum shows that **Ag**_**40**_ is racemic in DCM solution (Figure S4b). ESI-MS was performed; however, no meaningful signal of **Ag**_**40**_ was obtained in either the positive or negative ion mode (Figure S5). After the addition of cesium ions, the situation did not improve (Figure S5c). This may be due to the **Ag**_**40**_ nanocluster being neutral.

**Figure 1:**
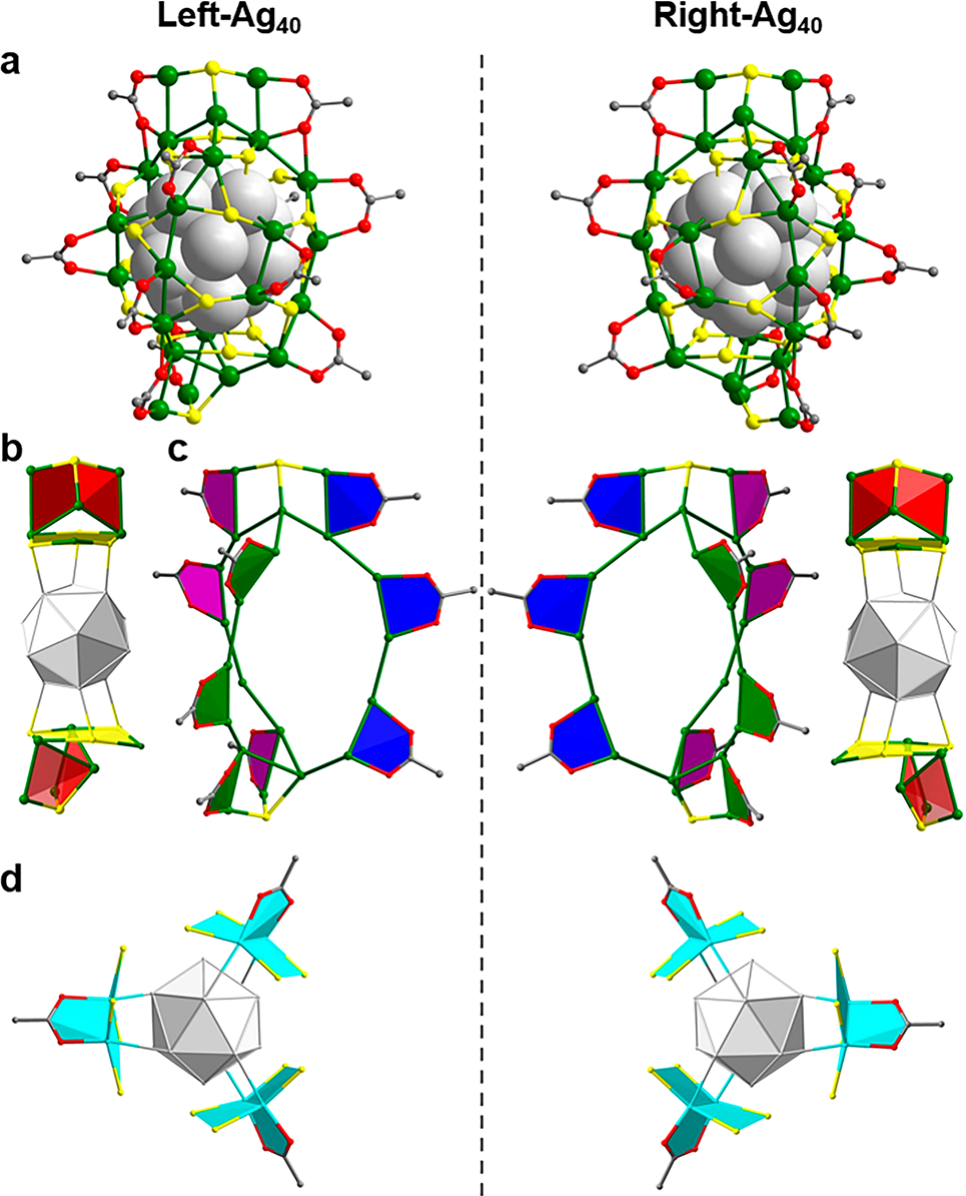
Chiral structure of the **Ag**_**40**_ nanoclusters. (a) X-ray structure of the two mirror-symmetric enantiomers of chiral **Ag**_**40**_. (b) Ag_5_(TBBM) units on top and bottom of **Ag**_**40**_. (c) Ag_21_(TBBM)_2_(CH_3_COO)_9_ spiral structure formed by three Ag_7_ chains in **Ag**_**40**_. (d) Spiral arrangement of Ag_2_(CH_3_COO)(TBBM)_4_ units at the waist of **Ag**_**40**_. Color codes: green or silver, Ag; yellow, S; red, O; gray, C. All H atoms and C atoms in TBBM are omitted for clarity.

The ratio between TBBM and acetic ligands were confirmed by ^1^H NMR in Figure S6. The ^1^H signals at 5.97–8.25 ppm (80 H = 20 × 4), 2.57–5.12 ppm (40 H = 20 × 2), and 0.58–1.48 ppm (180 H = 20 × 9) correspond to phenyl groups, benzyl groups, and tertiary butyl groups in TBBM. The ^1^H signals at 1.66–2.35 ppm (36 H = 12 × 3) correspond to the CH_3_COO^–^. These results are consistent with 20 TBBM and 12 CH_3_COO^–^ ligands as well as single-crystal X-ray diffraction (SC-XRD) in the **Ag**_**40**_ nanocluster. Additionally, X-ray photoelectron spectroscopy (XPS) revealed the elemental composition of **Ag**_**40**_, and semiquantitative analysis indicates the Ag/S/O ratio of 1:0.47:0.49, which is close to the 1:0.5:0.6 of the SC-XRD result (Figure S7). The EDS further proved the presence of Ag, S, and O elements in the **Ag**_**40**_ nanocluster (Figure S8), which was consistent with the cluster compositions obtained by SC-XRD. TGA was also performed to verify the ratio of metals to ligands in **Ag**_**40**_, and the experimental value of 49.49% was in good agreement with the theoretical value of 49.88% (Figure S9).

As shown in Figure 1, the origin of the chirality in the **Ag**_**40**_ lies in the asymmetric arrangement of one Ag_21_ framework (Figure 1c) and three Ag_2_(CH_3_COO)(TBBM)_4_ buttons (Figure 1d), whereas the icosahedral Ag_13_ kernel is achiral. Three chains are bonded to the top and the bottom of the Ag_21_(CH_3_COO)_9_(TBBM)_8_ framework by TBBM ligands, forming two orthogonal Ag_5_(TBBM) units. For the enantiomers, three Ag_2_(CH_3_COO)(TBBM)_4_ buttons form a left- and right-handed helical arrangement at the waist of **Ag**_**40**_ and are related by a *C*_3_ axis. From the top view, the helical arrangement of the Ag_2_(CH_3_COO)(TBBM)_4_ buttons resembles a triblade fan.

Silver or silver-doped nanoclusters undergo rapid racemization in solution.^47^ We rationalize that it should be possible for a rapidly racemizing nanocluster to produce a monochirality crystal during the crystallization. There are two processes involved: (i) synthesizing racemic silver nanoclusters; here, the newly obtained **Ag**_**40**_ nanocluster is chosen as a model in testing our chiral crystallization approach; (ii) screening the crystallization conditions to obtain one crystal in the solution.

In order to achieve rapid screening, we designed a set of high-throughput automated crystallization system (Figures 2 and S10). As such, a grain of homochiral **Ag**_**40**_ crystal was acquired and divided into five pieces (Figure 3). All pieces were homochiral and right-handed unraveled by SC-XRD. Furthermore, the homochiral **Ag**_**40**_ was found to crystallize in an orthorhombic *P*2_1_2_1_2_1_ space group. There are three important variables to be concerned with in this model: (1) Concentration of the DCM solution. To obtain **Ag**_**40**_ nanoclusters in one- and fine-crystal form, the concentration of **Ag**_**40**_ in the DCM solution is very crucial. When the solution is highly concentrated, it gives rise to the primary form of racemic crystals. Here, we control the concentrations to 3, 4, 5, 6, 7, 8, 9, and 10 mg/mL, respectively. (2) Ratio and volume of the diffusion layer. In this experiment, the ratio of the two-solvent diffusion layer is chosen as *V*_DCM_:*V*_CH_3_CN_ = 1:1, 1:2, and 1:3, and the volume of the diffusion layer is 0.5 or an equal multiple of the **Ag**_**40**_ DCM solution that is injected. Too much diffusion layer can make the crystal growth difficult. (3) Recrystallization temperature. To explore the temperature gradient influence, temperature gradients of 10, 20, and 30 °C are controlled to recrystallize **Ag**_**40**_ crystals. With the help of the automatic crystallization system, 144 vials of crystallization can be completed uniformly each time (Figures 2g,h and S10). All the crystallization results are shown in Table S1.

**Figure 2:**
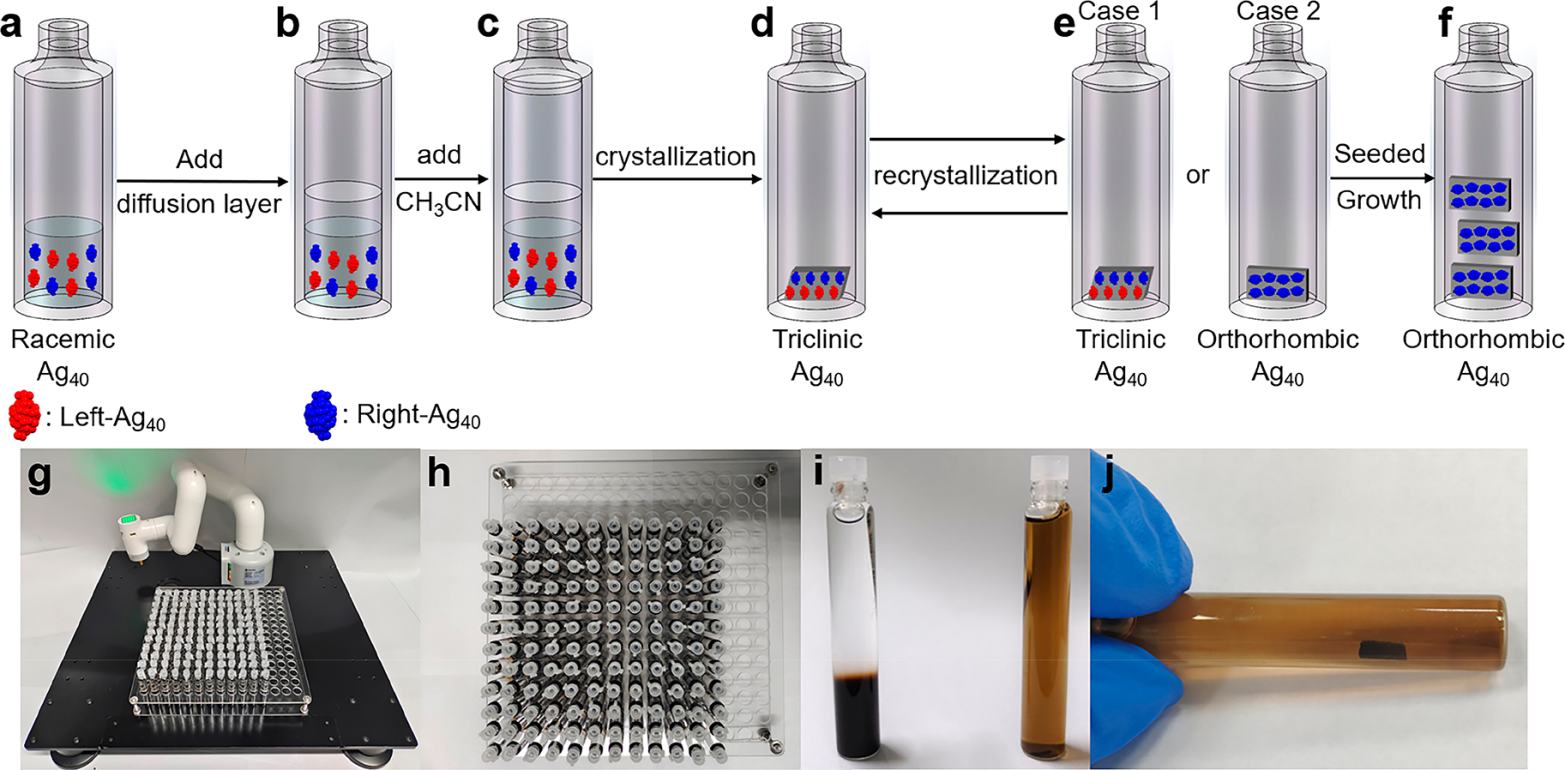
Illustration of crystallization. (a) Adding DCM solution of racemic **Ag**_**40**_. (b) Spread the diffusion layer above the DCM solution of **Ag**_**40**_. (c) Inject CH_3_CN to the top of the diffusion layer. (d) One triclinic **Ag**_**40**_ crystal resulted. (e) One triclinic **Ag**_**40**_ crystal is recrystallized and converted into one triclinic (Case 1) or homochiral **Ag**_**40**_ (Case 2) crystal by dissolution and recombination. (f) One homochiral crystal (Case 2) was used as seed to yield homochiral crystals. (g) Diagram of an automatic crystallization system. (h) Photograph of **Ag**_**40**_ crystals under different recrystallization conditions. (i) Photograph of **Ag**_**40**_ before and after recrystallization. (j) One homochiral **Ag**_**40**_ crystal after recrystallization.

**Figure 3:**
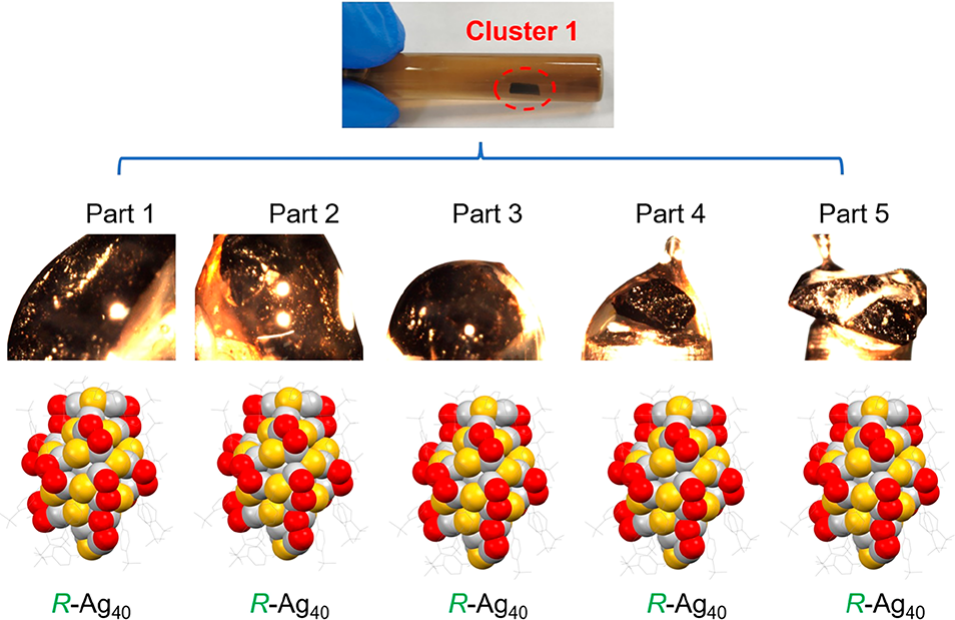
Photographs of the five parts from one homochiral **Ag**_**40**_ crystal. One homochiral **Ag**_**40**_ crystal is divided into five pieces (Parts 1–5), and their structures are all of the right-handed form (solved by SC-XRD). Color codes: silver, Ag; yellow, S; red, O; gray, C. All H atoms are omitted for clarity.

Interestingly, in the crystallization conditions of 30 °C, concentration of 4 mg/mL, and volume of the diffusion layer (*V*_DCM_:*V*_CH_3_CN_ = 1:1) was half of the DCM solution, homochiral **Ag**_**40**_ crystal was successfully acquired (Figure 2i,j). X-ray diffraction identified orthorhombic **Ag**_**40**_ crystals. This remarkable discovery indicates that the crystal system of **Ag**_**40**_ crystals is converted from the originally triclinic system (racemic) to the orthorhombic system (homochiral) after recrystallization. Further, **Ag**_**40**_ nanoclusters show excellent stability in the solution and solid state, which indicates that the clusters are not decomposed during the recrystallization (Figure S11). Under this set of crystallization conditions, more than 40 crystals from different vials were tested, and two of them were found to be homochiral (one crystal is left-handed and the other is right-handed) and orthorhombic (*P*2_1_2_1_2_1_), therefore the probability of racemic **Ag**_**40**_ nanoclusters being converted to one homochiral **Ag**_**40**_ crystal is 5% (Figure 2d,e).

Based on the principle that silver nanoclusters flip rapidly in solution, we have obtained homochiral **Ag**_**40**_ crystals. Meanwhile, this principle also provides the possibility for the amplification of chiral crystals. We used one homochiral **Ag**_**40**_ crystal as seed to make the racemic **Ag**_**40**_ clusters in solution convert into homochiral crystals by the seeded crystallization (Figure 2e,f). Simply, we first identified one homochiral **Ag**_**40**_ crystal (i.e., left-handed **Ag**_**40**_, abbrev. **L-Ag**_**40**_) by SC-XRD. Then, it was placed in a saturated DCM solution of **Ag**_**40**_ (racemic) and CH_3_CN was added; this **L-Ag**_**40**_ crystal remained in the crystalline state. After about 1 week, many **L-Ag**_**40**_ crystals were produced (Figure S1b). The morphology of all **L-Ag**_**40**_ crystals via seeded growth is similar to the homochiral **Ag**_**40**_ crystal and racemic **Ag**_**40**_ crystals, which is of black block (Figures 2j and S1). Expectedly, during the seeded crystallization process, racemic **Ag**_**40**_ clusters in solution rapidly flip, nucleate, and grow into homochiral crystals in the presence of one chiral crystal. We also note that the chirality of the crystals via seeded growth is consistent with the chirality of the initial seed.

It is interesting that the **Ag**_**40**_ nanoclusters which are crystallized in *P*2_1_2_1_2_1_ space group show optical activity in solid state but no activity in solution. This is due to the rapid racemization of the crystal upon redissolution. The CD spectra and corresponding UV–vis spectra of Racemic-**Ag**_**40**_ (**Rac-Ag**_**40**_), **L-Ag**_**40**_, and right-handed **Ag**_**40**_ (**R-Ag**_**40**_) crystals in solid state were collected at 250–800 nm (Figures 4a,b and S12a). The CD spectra of **L-Ag**_**40**_ and **R-Ag**_**40**_ are in mirror image of each other, confirming that they are pure enantiomers. As shown in Figure 4b, the **L-Ag**_**40**_ curve (blue) exhibits seven prominent peaks from 250 to 800 nm, including 261 (+), 283 (−), 326 (+), 386 (−), 432 (+), 469 (−), and 535 nm (+). The corresponding anisotropy factors *g* = Δ*A*/*A* = θ[mdeg]/(32980 × *A*) were calculated over the spectral range; *g*_max_ is about 5.9 × 10^–4^ at 261 nm (Figure 4c). It is worth noting that, using homochiral crystals (i.e., **L-Ag**_**40**_) as seeds, the obtained crystals have CD spectra similar to that of the original crystal (Figure 4d). Notably, the CD value of **L-Ag**_**40**_ seeded crystals decreased by approximately 10%, compared to the initial seed. Additionally, in order to better analyze the chirality of the samples, the corresponding UV–vis spectra and g-factors of **L-Ag**_**40**_ crystal and **L-Ag**_**40**_ seeded crystals in solid state were compared (Figures 4a, S12b, and S13). Notably, the g-factors of **L-Ag**_**40**_ seeded crystals decreased by about 16%. The *g*_max_ of **L-Ag**_**40**_ seeded crystals is about 4.5 × 10^–3^ at 260 nm. The possible reason might be that not all crystals were nucleated and grown with the added chiral crystal.

**Figure 4:**
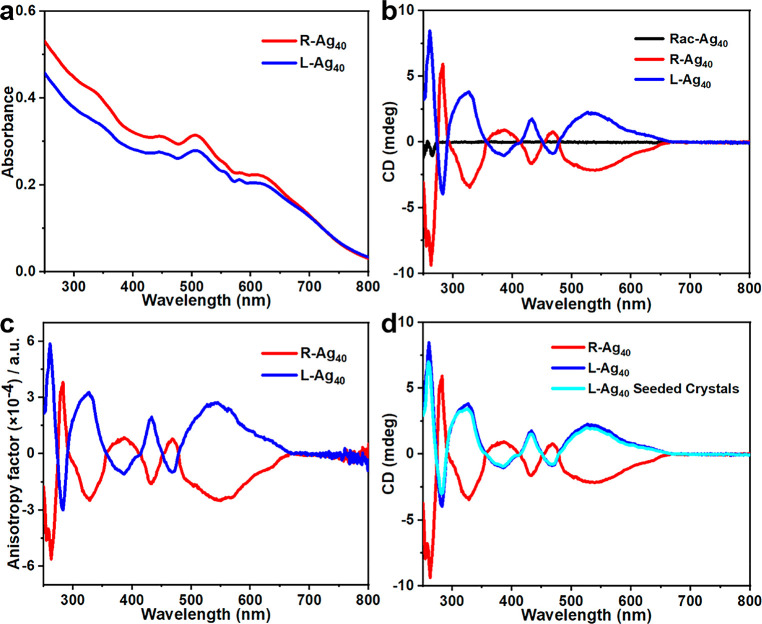
(a) UV–vis spectra of **R-Ag**_**40**_ crystals (red) and **L-Ag**_**40**_ crystals (blue) in solid state. (b) Experimental CD spectra of **Rac-Ag**_**40**_ crystals (black), **R-Ag**_**40**_ crystals (red), and **L-Ag**_**40**_ crystals (blue) in solid state. (c) Corresponding anisotropy factors of **R-Ag**_**40**_ and **L-Ag**_**40**_ enantiomers. (d) Experimental CD spectra of **R-Ag**_**40**_ crystals (red), **L-Ag**_**40**_ crystals (blue), and **L-Ag**_**40**_ seeded crystals (cyan).

There are 12 **Ag**_**40**_ molecules around one central **Ag**_**40**_ molecule in racemic or homochiral **Ag**_**40**_ crystals (Figures S14–S27). The density of the two crystals is calculated. After deducting the influence of the solvent, the density of the racemic **Ag**_**40**_ crystals is 1.6 g/cm^3^ and the homochiral is 1.9 g/cm^3^, explaining that homochiral **Ag**_**40**_ crystals has a higher packing density. For the intermolecular weak interactions, the C–H···π and π–π interactions play a vital role in the packing of **Ag**_**40**_ crystals.^52^ In racemic **Ag**_**40**_ crystals, only **Ag**_**40**_ molecules that are located in the *b*-axis direction have the C–H···π interaction with the central **Ag**_**40**_ molecule, as shown in Figure S28. The average distance for the C–H···π interaction is 3.073 Å for the left or right **Ag**_**40**_ molecule. In plane 1 of the *b*-axis direction of homochiral **Ag**_**40**_, the average distance of the eight C–H···π interactions is 3.466 Å (Figure S29). As shown in Figure S30, the average distance for the C–H···π interaction is 3.560 Å in plane 2 of the *c*-axis direction of homochiral **Ag**_**40**_. The π···π interactions are also discovered, and the average spacing is 5.023 Å. In homochiral **Ag**_**40**_ crystals, the 12 C–H···π interactions and four π···π interactions allow a central molecule to form a closed-ring structure with eight surrounding molecules in planes 1 and 2, which results in restrictions of the motions of the whole nanocluster. The connectivity via C–H···π and π···π interactions continues all the way along the *a*-axis of the unit cell, leading to the formation of the needle-like single crystal along the [100] direction. By contrast, C–H···π interactions exist only in a single plane along the [010] direction, which makes a less compact packing in racemic **Ag**_**40**_ crystals. Under the recrystallization, it is highly likely to obtain the racemic **Ag**_**40**_ crystal of the *P*1̅ space group. With a difference in the kinetic barrier (Ea, high for homochiral crystals), such crystals are not easy to form. This also shows that the acquisition of chiral crystals is a thermodynamic process in this case: higher temperatures favor the denser phase, which happens to be the chiral phase.

Furthermore, the **Ag**_**40**_ nanocluster could react with chiral 2-chloropropionic acid/ibuprofen/naproxen to result in an optically pure enantiomer. ^1^H NMR and CD spectra showed that the CH_3_COO^–^ ligand on the **Ag**_**40**_ surface could be rapidly replaced by these chiral acids (Figures S31–S50). The UV–vis spectra of **Ag**_**40**_ did not change with ligand exchange, indicating that the structure of **Ag**_**40**_ was retained (Figures S46a and S50b). The CD spectra of **Ag**_**40**_ (R-2-chloropropionic acid) and **Ag**_**40**_ (S-2-chloropropionic acid) showed symmetrical peaks at 241, 275, 337, 423, 462, 507, and 623 nm, respectively (Figure S46b). The anisotropy factors were calculated, and *g*_max_ is about 1.1 × 10^–3^ at 343 nm (Figure S46c), which is comparable to that of chiral silver nanoclusters.^53,54^ When using **Ag**_**40**_ to detect chiral compounds such as ibuprofen or naproxen, the maximum CD signal can be obtained when the molar ratio of the chiral compound is around 7 times higher than the cluster (Figure S47c,d). The ^1^H NMR results showed an average of ∼7 ibuprofen or naproxen per cluster (Figures S39, S40, S43, and S44). For chiral 2-chloropropionic acid, the CD signals approached the maximum at 1:12 (Figure S47a). The ^1^H NMR results showed an average of ∼6 “2-chloropropionic acid” per cluster (Figures S34 and S36). These results indicate that chiral inversion can be achieved in the **Ag**_**40**_ clusters if approximately half of the carboxyl groups are replaced by chiral acids. In order to prove that excessive chiral acids can cause the chiral inversion of **Ag**_**40**_ nanoclusters, the ligand exchange between the homochiral **Ag**_**40**_ (chiral acids) and the chiral acids with the opposite chirality and the corresponding CD spectra confirm that the opposite chirality can be induced (Figure S48).^48^ Meanwhile, the enantiomer-dependent CD intensity in the R/S-2-chloropropionic acid ligand-exchange process also linearly correlates with the ee values, and the detection range was from 0 to 100% ee (Figure 5a,b). Notably, R/S-2-chloropropionic acid in CH_2_Cl_2_ exhibited only one CD signal at 232 nm (Figure S49), which is close to the far-ultraviolet region and requires high sensitivity for the instrument. For R/S-2-chloropropionic acid, the **Ag**_**40**_ cluster can effectively extend the detection range from deep ultraviolet to visible light (Figure S47a). Meanwhile, the **Ag**_**40**_ nanocluster can be used as chiral amplifiers for chiral carboxyl drugs such as R/S-ibuprofen and R/S-naproxen (Figures S50 and S51).

**Figure 5:**
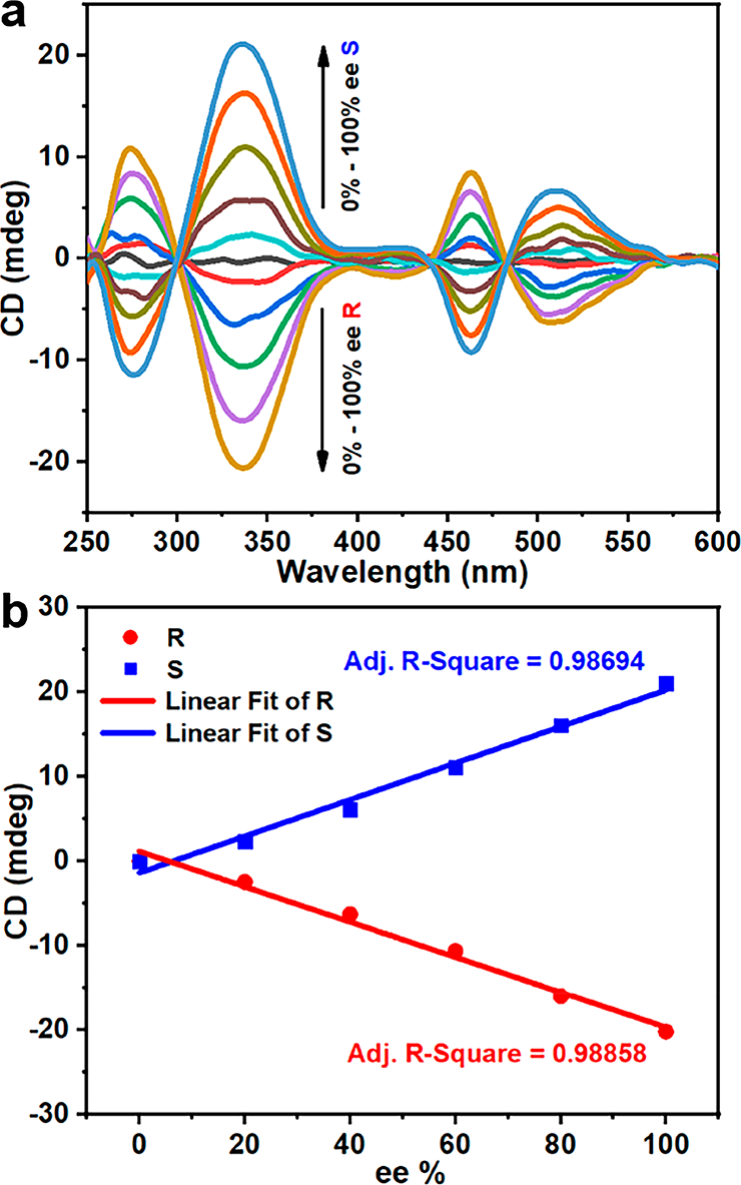
(a) CD spectra of **Ag**_**40**_ and R/S-2-chloropropionic acid of different ee% (0, 20, 40, 60, 80, 100%) in CH_2_Cl_2_. (b) The fitted curve by plotting CD readings at 337 nm in panel a against ee values of chiral R/S-2-chloropropionic acid.

In summary, the **Ag**_**40**_ nanoclusters are analyzed by SC-XRD, UV–vis, ^1^H NMR, XPS, TGA, ESI-MS, and CD. Chiral symmetry breaking, absolute asymmetric synthesis, and the amplification of chiral crystals in metal nanoclusters from racemic **Ag**_**40**_ nanoclusters to homochiral ones are accomplished with the help of the crystallization model. In addition, **Ag**_**40**_ nanoclusters can be used as chiral amplifiers and provide guidance for cluster-based chiral sensors. Our work demonstrates the possibility of homochiral construction without the influence of chiral factors (including ligands, environment, etc.), nor the external forces. Further, the seeded crystallization method proved that the effect of chiral crystal seed on the chirality construction of final products and amplification of chiral crystals were achieved. The results provide important implications for the transformation of chirality between nanoclusters, chiral separation, the amplification of chiral crystals, chiral detection, and the origin of chirality generation.

## Supporting Information Available

Additional experimental details, materials, and methods, including supporting Figures S1–S51 and Tables S1–S8 (PDF)

## References

[ref1] Morrow S. M.; Bissette A. J.; Fletcher S. P. Transmission of chirality through space and across length scales. Nat. Nanotechnol. 2017, 12 (5), 410–419. 10.1038/nnano.2017.62. 28474691

[ref2] Ma W.; Xu L. G.; de Moura A. F.; Wu X. L.; Kuang H.; Xu C. L.; Kotov N. A. Chiral Inorganic Nanostructures. Chem. Rev. 2017, 117 (12), 8041–8093. 10.1021/acs.chemrev.6b00755. 28426196

[ref3] Wang Y.; Xu J.; Wang Y. W.; Chen H. Y. Emerging chirality in nanoscience. Chem. Soc. Rev. 2013, 42 (7), 2930–2962. 10.1039/C2CS35332F. 23207678

[ref4] Pop F.; Zigon N.; Avarvari N. Main-Group-Based Electro-and Photoactive Chiral Materials. Chem. Rev. 2019, 119 (14), 8435–8478. 10.1021/acs.chemrev.8b00770. 30943018

[ref5] Zhang L.; Wang T. Y.; Shen Z. C.; Liu M. H. Chiral Nanoarchitectonics: Towards the Design, Self-Assembly, and Function of Nanoscale Chiral Twists and Helices. Adv. Mater. 2016, 28 (6), 1044–1059. 10.1002/adma.201502590. 26385875

[ref6] Soai K.; Niwa S.; Hori H. Asymmetric self-catalytic reaction. Self-production of chiral 1-(3-pyridyl)alkanols as chiral self-catalysts in the enantioselective addition of dialkylzinc reagents to pyridine-3-carbaldehyde. J. Chem. Soc., Chem. Commun. 1990, 14, 982–983. 10.1039/c39900000982.

[ref7] Soai K.; Kawasaki T.; Matsumoto A. Asymmetric Autocatalysis of Pyrimidyl Alkanol and Its Application to the Study on the Origin of Homochirality. Acc. Chem. Res. 2014, 47 (12), 3643–3654. 10.1021/ar5003208. 25511374

[ref8] Kondepudi D. K.; Kaufman R. J.; Singh N. Chiral symmetry breaking in sodium chlorate crystallizaton. Science 1990, 250 (4983), 975 10.1126/science.250.4983.975. 17746924

[ref9] Zhao L.; Belvin C. A.; Liang R.; Bonn D. A.; Hardy W. N.; Armitage N. P.; Hsieh D. A global inversion-symmetry-broken phase inside the pseudogap region of YBa_2_Cu_3_O_y_. Nat. Phys. 2017, 13 (3), 250–254. 10.1038/nphys3962.

[ref10] Viedma C.; Cintas P. Homochirality beyond grinding: deracemizing chiral crystals by temperature gradient under boiling. Chem. Commun. 2011, 47 (48), 12786–12788. 10.1039/c1cc14857e. 22053323

[ref11] Xiouras C.; Fytopoulos A.; Jordens J.; Boudouvis A. G.; Van Gerven T.; Stefanidis G. D. Applications of ultrasound to chiral crystallization, resolution and deracemization. Ultrason. Sonochem. 2018, 43, 184–192. 10.1016/j.ultsonch.2018.01.014. 29555274

[ref12] Eder R. J. P.; Schmitt E. K.; Grill J.; Radl S.; Gruber-Woelfler H.; Khinast J. G. Seed loading effects on the mean crystal size of acetylsalicylic acid in a continuous-flow crystallization device. Cryst. Res. Technol. 2011, 46 (3), 227–237. 10.1002/crat.201000634.

[ref13] Qian R.-Y.; Botsaris G. D. Nuclei breeding from a chiral crystal seed of NaClO_3_. Chem. Eng. Sci. 1998, 53 (9), 1745–1756. 10.1016/S0009-2509(98)00040-2.

[ref14] Jadzinsky P. D.; Calero G.; Ackerson C. J.; Bushnell D. A.; Kornberg R. D. Structure of a thiol monolayer-protected gold nanoparticle at 1.1 angstrom resolution. Science 2007, 318 (5849), 430–433. 10.1126/science.1148624. 17947577

[ref15] Huang R.-W.; Wei Y.-S.; Dong X.-Y.; Wu X.-H.; Du C.-X.; Zang S.-Q.; Mak T. C. W. Hypersensitive dual-function luminescence switching of a silver-chalcogenolate cluster-based metal-organic framework. Nat. Chem. 2017, 9 (7), 689–697. 10.1038/nchem.2718. 28644463

[ref16] Desireddy A.; Conn B. E.; Guo J. S.; Yoon B.; Barnett R. N.; Monahan B. M.; Kirschbaum K.; Griffith W. P.; Whetten R. L.; Landman U.; Bigioni T. P. Ultrastable silver nanoparticles. Nature 2013, 501 (7467), 399–402. 10.1038/nature12523. 24005327

[ref17] Petty J. T.; Zheng J.; Hud N. V.; Dickson R. M. DNA-templated Ag nanocluster formation. J. Am. Chem. Soc. 2004, 126 (16), 5207–5212. 10.1021/ja031931o. 15099104

[ref18] Liu C.; Li T.; Abroshan H.; Li Z.; Zhang C.; Kim H. J.; Li G.; Jin R. Chiral Ag_23_ nanocluster with open shell electronic structure and helical face-centered cubic framework. Nat. Commun. 2018, 9, 744 10.1038/s41467-018-03136-9. 29467372 PMC5821857

[ref19] Conn B. E.; Atnagulov A.; Yoon B.; Barnett R. N.; Landman U.; Bigioni T. P. Confirmation of a de novo structure prediction for an atomically precise monolayer-coated silver nanoparticle. Sci. Adv. 2016, 2 (11), e1601609 10.1126/sciadv.1601609. 28138537 PMC5262450

[ref20] Wan X. K.; Wang J. Q.; Nan Z. A.; Wang Q. M. Ligand effects in catalysis by atomically precise gold nanoclusters. Sci. Adv. 2017, 3 (10), e1701823 10.1126/sciadv.1701823. 28989966 PMC5630233

[ref21] Chakraborty I.; Pradeep T. Atomically Precise Clusters of Noble Metals: Emerging Link between Atoms and Nanoparticles. Chem. Rev. 2017, 117 (12), 8208–8271. 10.1021/acs.chemrev.6b00769. 28586213

[ref22] Joshi C. P.; Bootharaju M. S.; Alhilaly M. J.; Bakr O. M. [Ag_25_(SR)_18_]^−^: The ″Golden″ Silver Nanoparticle. J. Am. Chem. Soc. 2015, 137 (36), 11578–11581. 10.1021/jacs.5b07088. 26322865

[ref23] Jin R.; Zeng C. J.; Zhou M.; Chen Y. X. Atomically Precise Colloidal Metal Nanoclusters and Nanoparticles: Fundamentals and Opportunities. Chem. Rev. 2016, 116 (18), 10346–10413. 10.1021/acs.chemrev.5b00703. 27585252

[ref24] Kwak K.; Choi W.; Tang Q.; Kim M.; Lee Y.; Jiang D. E.; Lee D. A molecule-like PtAu_24_(SC_6_H_13_)_18_ nanocluster as an electrocatalyst for hydrogen production. Nat. Commun. 2017, 8, 14723 10.1038/ncomms14723. 28281526 PMC5353570

[ref25] Liu Z. H.; Wu Z. N.; Yao Q. F.; Cao Y. T.; Chai O. J. H.; Xie J. P. Correlations between the fundamentals and applications of ultrasmall metal nanoclusters: Recent advances in catalysis and biomedical applications. Nano Today 2021, 36, 101053 10.1016/j.nantod.2020.101053.

[ref26] Ma X. S.; Xiong L.; Qin L. B.; Tang Y.; Ma G. Y.; Pei Y.; Tang Z. H. A homoleptic alkynyl-protected [Ag_9_Cu_6_(^t^BuC≡C)_12_]^+^ superatom with free electrons: synthesis, structure analysis, and different properties compared with the Au_7_Ag_8_ cluster in the M_15_^+^ series. Chem. Sci. 2021, 12 (38), 12819–12826. 10.1039/D1SC03679C. 34703569 PMC8494057

[ref27] Tang Q.; Lee Y. J.; Li D. Y.; Choi W.; Liu C. W.; Lee D.; Jiang D. E. Lattice-Hydride Mechanism in Electrocatalytic CO_2_ Reduction by Structurally Precise Copper-Hydride Nanoclusters. J. Am. Chem. Soc. 2017, 139 (28), 9728–9736. 10.1021/jacs.7b05591. 28640611

[ref28] Wang S. T.; Gao X. H.; Hang X. X.; Zhu X. F.; Han H. T.; Liao W. P.; Chen W. Ultrafine Pt Nanoclusters Confined in a Calixarene-Based {Ni_24_} Coordination Cage for High-Efficient Hydrogen Evolution Reaction. J. Am. Chem. Soc. 2016, 138 (50), 16236–16239. 10.1021/jacs.6b11218. 27935678

[ref29] Narouz M. R.; Osten K. M.; Unsworth P. J.; Man R. W. Y.; Salorinne K.; Takano S.; Tomihara R.; Kaappa S.; Malola S.; Dinh C.-T.; Padmos J. D.; Ayoo K.; Garrett P. J.; Nambo M.; Horton J. H.; Sargent E. H.; Häkkinen H.; Tsukuda T.; Crudden C. M. N-heterocyclic carbene-functionalized magic-number gold nanoclusters. Nat. Chem. 2019, 11 (5), 419–425. 10.1038/s41557-019-0246-5. 30988416

[ref30] Chen S.; Ma H. D.; Padelford J. W.; Qinchen W. L.; Yu W.; Wang S. X.; Zhu M. Z.; Wang G. Near Infrared Electrochemiluminescence of Rod-Shape 25-Atom AuAg Nanoclusters That Is Hundreds-Fold Stronger Than That of Ru(bpy)_3_ Standard. J. Am. Chem. Soc. 2019, 141 (24), 9603–9609. 10.1021/jacs.9b02547. 31184150

[ref31] Wu Z. L.; Hu G. X.; Jiang D. E.; Mullins D. R.; Zhang Q. F.; Allard L. F.; Wang L. S.; Overbury S. H. Diphosphine-Protected Au_22_ Nanoclusters on Oxide Supports Are Active for Gas-Phase Catalysis without Ligand Removal. Nano Lett. 2016, 16 (10), 6560–6567. 10.1021/acs.nanolett.6b03221. 27685318

[ref32] Cook A. W.; Jones Z. R.; Wu G.; Scott S. L.; Hayton T. W. An Organometallic Cu_20_ Nanocluster: Synthesis, Characterization, Immobilization on Silica, and ″Click″ Chemistry. J. Am. Chem. Soc. 2018, 140 (1), 394–400. 10.1021/jacs.7b10960. 29211459

[ref33] Liang X. Q.; Li Y. Z.; Wang Z.; Zhang S. S.; Liu Y. C.; Cao Z. Z.; Feng L.; Gao Z. Y.; Xue Q. W.; Tung C. H.; Sun D. Revealing the chirality origin and homochirality crystallization of Ag_14_ nanocluster at the molecular level. Nat. Commun. 2021, 12, 4966 10.1038/s41467-021-25275-2. 34404784 PMC8371133

[ref34] Yan J. Z.; Su H. F.; Yang H. Y.; Hu C. Y.; Malola S.; Lin S. C.; Teo B. K.; Hakkinen H.; Zheng N. F. Asymmetric Synthesis of Chiral Bimetallic [Ag_28_Cu_12_(SR)_24_]^4-^ Nanoclusters via Ion Pairing. J. Am. Chem. Soc. 2016, 138 (39), 12751–12754. 10.1021/jacs.6b08100. 27626935

[ref35] Zhu M. Z.; Qian H. F.; Meng X. M.; Jin S. S.; Wu Z. K.; Jin R. Chiral Au_25_ Nanospheres and Nanorods: Synthesis and Insight into the Origin of Chirality. Nano Lett. 2011, 11 (9), 3963–3969. 10.1021/nl202288j. 21834520

[ref36] Yang H. Y.; Yan J. Z.; Wang Y.; Deng G. C.; Su H. F.; Zhao X. J.; Xu C. F.; Teo B. K.; Zheng N. F. From Racemic Metal Nanoparticles to Optically Pure Enantiomers in One Pot. J. Am. Chem. Soc. 2017, 139 (45), 16113–16116. 10.1021/jacs.7b10448. 29053274

[ref37] Li S.; Du X. S.; Li B.; Wang J. Y.; Li G. P.; Gao G. G.; Zang S. Q. Atom-Precise Modification of Silver(I) Thiolate Cluster by Shell Ligand Substitution: A New Approach to Generation of Cluster Functionality and Chirality. J. Am. Chem. Soc. 2018, 140 (2), 594–597. 10.1021/jacs.7b12136. 29281275

[ref38] Shen H.; Xu Z.; Wang L. Z.; Han Y. Z.; Liu X. H.; Malola S.; Teo B. K.; Hakkinen H.; Zheng N. F. Tertiary Chiral Nanostructures from C-H···F Directed Assembly of Chiroptical Superatoms. Angew. Chem., Int. Ed. 2021, 60 (41), 22411–22416. 10.1002/anie.202108141. 34347339

[ref39] Liu W. D.; Wang J. Q.; Yuan S. F.; Chen X.; Wang Q. M. Chiral Superatomic Nanoclusters Ag_47_ Induced by the Ligation of Amino Acids. Angew. Chem., Int. Ed. 2021, 60 (20), 11430–11435. 10.1002/anie.202100972. 33629455

[ref40] Sugiuchi M.; Shichibu Y.; Konishi K. An Inherently Chiral Au_24_ Framework with Double-Helical Hexagold Strands. Angew. Chem., Int. Ed. 2018, 57 (26), 7855–7859. 10.1002/anie.201804087. 29719106

[ref41] Dolamic I.; Knoppe S.; Dass A.; Burgi T. First enantioseparation and circular dichroism spectra of Au_38_ clusters protected by achiral ligands. Nat. Commun. 2012, 3, 798 10.1038/ncomms1802. 22531183 PMC3337976

[ref42] Zeng C.; Li T.; Das A.; Rosi N. L.; Jin R. Chiral Structure of Thiolate-Protected 28-Gold-Atom Nanocluster Determined by X-ray Crystallography. J. Am. Chem. Soc. 2013, 135 (27), 10011–10013. 10.1021/ja404058q. 23815445

[ref43] Knoppe S.; Dolamic I.; Dass A.; Burgi T. Separation of Enantiomers and CD Spectra of Au_40_(SCH_2_CH_2_Ph)_24_: Spectroscopic Evidence for Intrinsic Chirality. Angew. Chem., Int. Ed. 2012, 51 (30), 7589–7591. 10.1002/anie.201202369. 22707380

[ref44] Zhu Y. F.; Wang H.; Wan K. W.; Guo J.; He C. T.; Yu Y.; Zhao L. Y.; Zhang Y.; Lv J. W.; Shi L.; Jin R. X.; Zhang X. X.; Shi X. H.; Tang Z. Y. Enantioseparation of Au_20_(PP_3_)_4_Cl_4_ Clusters with Intrinsically Chiral Cores. Angew. Chem., Int. Ed. 2018, 57 (29), 9059–9063. 10.1002/anie.201805695. 29877009

[ref45] Knoppe S.; Wong O. A.; Malola S.; Hakkinen H.; Burgi T.; Verbiest T.; Ackerson C. J. Chiral Phase Transfer and Enantioenrichment of Thiolate-Protected Au_102_ Clusters. J. Am. Chem. Soc. 2014, 136 (11), 4129–4132. 10.1021/ja500809p. 24588769

[ref46] Knoppe S.; Dolamic I.; Buergi T. Racemization of a Chiral Nanoparticle Evidences the Flexibility of the Gold-Thiolate Interface. J. Am. Chem. Soc. 2012, 134 (31), 13114–13120. 10.1021/ja3053865. 22793992

[ref47] Zhang B.; Burgi T. Doping Silver Increases the Au_38_(SR)_24_ Cluster Surface Flexibility. J. Phys. Chem. C 2016, 120 (8), 4660–4666. 10.1021/acs.jpcc.5b12690.

[ref48] Chai J.; Yang S.; Chen T.; Li Q.; Wang S.; Zhu M. Chiral Inversion and Conservation of Clusters: A Case Study of Racemic Ag_32_Cu_12_ Nanocluster. Inorg. Chem. 2021, 60 (12), 9050–9056. 10.1021/acs.inorgchem.1c01049. 34061506

[ref49] Bootharaju M. S.; Joshi C. P.; Alhilaly M. J.; Bakr O. M. Switching a Nanocluster Core from Hollow to Nonhollow. Chem. Mater. 2016, 28 (10), 3292–3297. 10.1021/acs.chemmater.5b05008.

[ref50] Soai K.; Shibata T.; Morioka H.; Choji K. Asymmetric autocatalysis and amplification of enantiomeric excess of a chiral molecule. Nature 1995, 378 (6559), 767–768. 10.1038/378767a0.

[ref51] Du W. J.; Kang X.; Jin S.; Liu D. Y.; Wang S. X.; Zhu M. Z. Different Types of Ligand Exchange Induced by Au Substitution in a Maintained Nanocluster Template. Inorg. Chem. 2020, 59 (3), 1675–1681. 10.1021/acs.inorgchem.9b02792. 31944677

[ref52] He L. Z.; Gan Z. B.; Xia N.; Liao L. W.; Wu Z. K. Alternating Array Stacking of Ag_26_Au and Ag_24_Au Nanoclusters. Angew. Chem., Int. Ed. 2019, 58 (29), 9897–9901. 10.1002/anie.201900831. 31070836

[ref53] Liu W.-D.; Wang J.-Q.; Yuan S.-F.; Chen X.; Wang Q.-M. Chiral Superatomic Nanoclusters Ag_47_ Induced by the Ligation of Amino Acids. Angew. Chem., Int. Ed. 2021, 60 (20), 11430–11435. 10.1002/anie.202100972. 33629455

[ref54] Yoshida H.; Ehara M.; Priyakumar U. D.; Kawai T.; Nakashima T. Enantioseparation and chiral induction in Ag_29_ nanoclusters with intrinsic chirality. Chem. Sci. 2020, 11 (9), 2394–2400. 10.1039/C9SC05299B. 34084402 PMC8157427

