# Supplementary material for: Transforming Silver Nanoclusters from Racemic to Homochiral via Seeded Crystallization
Source: J Phys Chem Lett. 2023 May 26;14(22):5095–101. doi: 10.1021/acs.jpclett.3c00794 (PMC10258841; doi:10.1021/acs.jpclett.3c00794)
Supplement: Supplementary file 1 — jz3c00794_si_001.pdf [file jz3c00794_si_001.pdf]

Supporting Information

Transforming Silver Nanoclusters from Racemic to Homochiral via Seeded Crystallization

Along Ma[1], Wenjun Du[3], Jiawei Wang[1], Kefan Jiang[1], Cheng Zhang[1], Wenhan Sheng[1], Haiyan Zheng[1], Rongchao Jin[2,*], and Shuxin Wang[1,*]

[1]College of Materials Science and Engineering, Qingdao University of Science and Technology, Qingdao, Shandong 266042, P. R. China

[2]Department of Chemistry, Carnegie Mellon University, Pittsburgh, Pennsylvania 15213, United States

[3]Anhui Provincial Key Laboratory for Degradation and Monitoring of Pollution of the Environment, School of Chemistry and Materials Engineering, Fuyang Normal University, Fuyang 236037, P. R. China

*Corresponding authors. E-mail: rongchao@andrew.cmu.edu (Rongchao Jin); shuxin_wang@qust.edu.cn (Shuxin Wang)

Chemicals and Materials. All reagents were commercially available and used without further purification. Silver acetate (CH_3COOAg, 99.5% metals basis), 4-tert-butylbenzyl mercaptan ($HSCH_2Ph\text{-}^tBu$, TBBM, 98%), sodium borohydride ($NaBH_4$, 98%), triethylamine ($(C_2H_5)_3N$, 99%), cesium acetate (CH_3COOCs, 98%), R-2-chloropropionic acid ($C_3H_5ClO_2$, 98%), S-2-chloropropionic acid ($C_3H_5ClO_2$, 98%), R-ibuprofen ($C_{13}H_{18}O_2$, 98%), S-ibuprofen ($C_{13}H_{18}O_2$, 98%), R-naproxen ($C_{14}H_{14}O_3$, 99%), S-naproxen ($C_{14}H_{14}O_3$, 99%), acetonitrile (CH_3CN, 99.0%), methyl alcohol (MeOH, 99.5%), ethyl alcohol (EtOH, 99.7%), dichloromethane (DCM, 99.5%), and n-hexane (Hex, 97.0%), were purchased from Sigma-Aldrich.

Synthesis of racemic Ag_{40} NC. Typically, 15 mL MeOH solution of silver acetate (60 mg, 0.36 mmol) firstly was injected into the flask. After stirring for 10 min, TBBM (18.5 μL, 0.095 mmol) ligand dissolved in 1 mL MeOH was introduced by injection pump, resulting in a yellow solution of metal-thiolate in 40 min. $NaBH_4$ (12.5 mg, 0.33 mmol) and $(C_2H_5)_3N$ (5 μL, 0.036 mmol) dissolved in 2 mL of EtOH was added into the solution. The reaction was continuously stirred for 20 h at room temperature. After that, in order to obtain the pure $\mathbf{Ag_{40}}$ nanoclusters, the solution was centrifuged, and the precipitate was dissolved in *n*-hexane

(10 mL). Then, the solution of *n*-hexane was centrifuged and the supernatant was concentrated using a rotary evaporator. The acquired solid was repeatedly washed with CH_3CN and dried again to obtain a powder of pure **Ag_{40}** nanoclusters. Black, block-shaped crystals were acquired by crystallizing the pure nanoclusters in CH_2Cl_2/CH_3CN (1 : 2) after ~4 days at room temperature. The yield of **Ag_{40}** is ∼35% (on the Ag atom basis).

Conversion of racemic Ag_{40} to homochiral Ag_{40}. Homochiral **Ag_{40}** was obtained by recrystallizing racemic **Ag_{40}** crystals. Typically, 80 mg of racemic **Ag_{40}** crystals were dissolved in 20 mL of DCM solvent with a concentration of 4 mg/mL. Glass vials (3 mL) were used in crystallization. Typically, 1 mL DCM solution of racemic **Ag_{40}** was injected into each vial. Then, 0.5 mL of diffusion layer was spread on top of it, note: the diffusion layer is composed of DCM and CH_3CN in a volume ratio of 1:1. Finally, 1.5 mL of CH_3CN was slowly transfused into each vial. Homochiral **Ag_{40}** crystals were obtained after ~ a week at 30 ^oC. More than 40 crystals from different vials were tested and two of them were found to be homochiral and orthorhombic ($P2_12_12_1$), it should be noted that the two crystals come from two different vials and that only one crystal was formed in a vial and the two crystals were homochiral. Therefore, the probability of racemic **Ag_{40}** nanoclusters being converted to one homochiral **Ag_{40}** crystal is 2/40 = 5%.

Seed crystal method to yielding homochiral Ag_{40} crystals. First, 750 mg of racemic **Ag_{40}** crystals were mixed with 1.5 mL of DCM and stirred for 10 min. Then, the mixture was centrifuged and the precipitate (~20 mg) was removed to obtain a saturated solution of **Ag_{40}** in DCM. Next, we identified a homochiral (orthorhombic, $P2_12_12_1$) **Ag_{40}** crystal with SC-XRD. The homochiral **Ag_{40}** crystal was used as the seed and placed in a vial. Then 1 mL of saturated solution of **Ag_{40}** in DCM was slowly added, followed by 2 mL of CH_3CN. Homochiral **Ag_{40}** crystals were obtained after ~one week. The yield of homochiral **Ag_{40}** is ~90%. The chirality of the enlarged crystals is consistent with the chirality of the seed.

Chiral Amplification of 2-Chloropropionic acid/ibuprofen/naproxen. Typically, 1 mL of CH_2Cl_2 solution of R/S-2-chloropropionic acid (5.8×10^{-6} mol/L) was added to 1 mL of a CH_2Cl_2 solution of **Ag_{40}** (6.96×10^{-5} mol/L). The mixture was stirred at room temperature and directly used for the CD measurement.

Characterization. Ultraviolet-visible absorption spectra in this study were recorded on a Shanghai Metash UV-8000 spectrophotometer and all samples were dissolved in DCM for spectrum measurements. Electrospray ionization time-of-flight mass spectrometry (ESI-TOF-MS) measurements were carried out on a Bruker micro TOF-Q system in positive or negative ion mode in the range m/z = 2000−14000. To prepare the ESI sample, clusters were dissolved in CH_2Cl_2/CH_3OH (v/v = 1:3) and diluted in the same solvent to a concentration of approximately 10 ppm and the molar ratio of $\mathbf{Ag_{40}}$ nanoclusters to CH_3COOCs is 1 : 30. The X-ray photoelectron spectroscopy (XPS) measurements were performed on ESCALAB XI+ configured with a monochromated AlKα (1486.8 eV) 150W X-ray source, 0.5 mm circular spot size, a flood gun to counter charging effects, and the analysis chamber base pressure lower than 1 $x10^{-9}$ mbar, data were collected with FAT = 20 eV. Nuclear magnetic resonance (NMR) measurements were performed on a Bruker Avance spectrometer instrument operating at 500 MHz for ^1H. All data were collected with 8 mg of products dissolved in 0.6 mL of $CDCl_3$. Thermogravimetric analysis (TGA) was carried out on a thermogravimetric analyzer (DTG-60H) with ~8 mg of nanoclusters in a SiO_2 pan at a heating rate of 10 K/min under an N_2 atmosphere. And the sample was extracted for 2 h to remove the solvent molecules using a vacuum pump, before TGA testing. Circular dichroism (CD) spectra in CH_2Cl_2 solution were measured using a J-1500 CD spectrometer in a 1 cm path. Circular dichroism (CD) spectra in solid state were recorded with a BioLogic MOS-500 CD spectropolarimeter and one crystal were dispersed and placed between two quartz plates for testing. Analysis of Single-Crystal X-ray Diffraction: The crystal data of racemic $\mathbf{Ag_{40}}$ and homochiral $\mathbf{Ag_{40}}$ were collected on a Rigaku Oxford Diffraction. Crystal data and structure refinement for all crystals are summarized in tables S2-S8. The intensity diffraction data of nanoclusters were collected on a Rigaku Agilent SuperNova Dual system at 170 K. Using Olex2,[1] the structures were solved with the ShelXT structure solution program using Intrinsic Phasing and refined with the ShelXL refinement package using Least Squares minimization.[2] Non-hydrogen atoms were refined anisotropically. The hydrogen atoms of organic ligands were generated geometrically. SQUEEZE routines in PLATON were employed to deal with undefined disordered solvent molecules.

Supplementary Figures and Tables

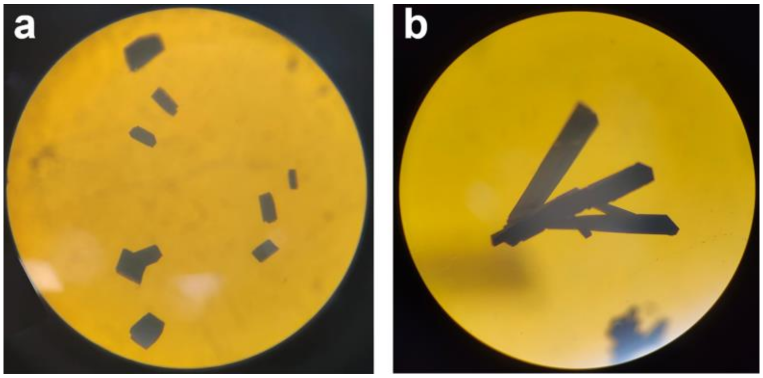

Fig. S1 The optical microscopic images. (a) Racemic **Ag_{40}**. (b) **L-Ag_{40}** seeded crystals.

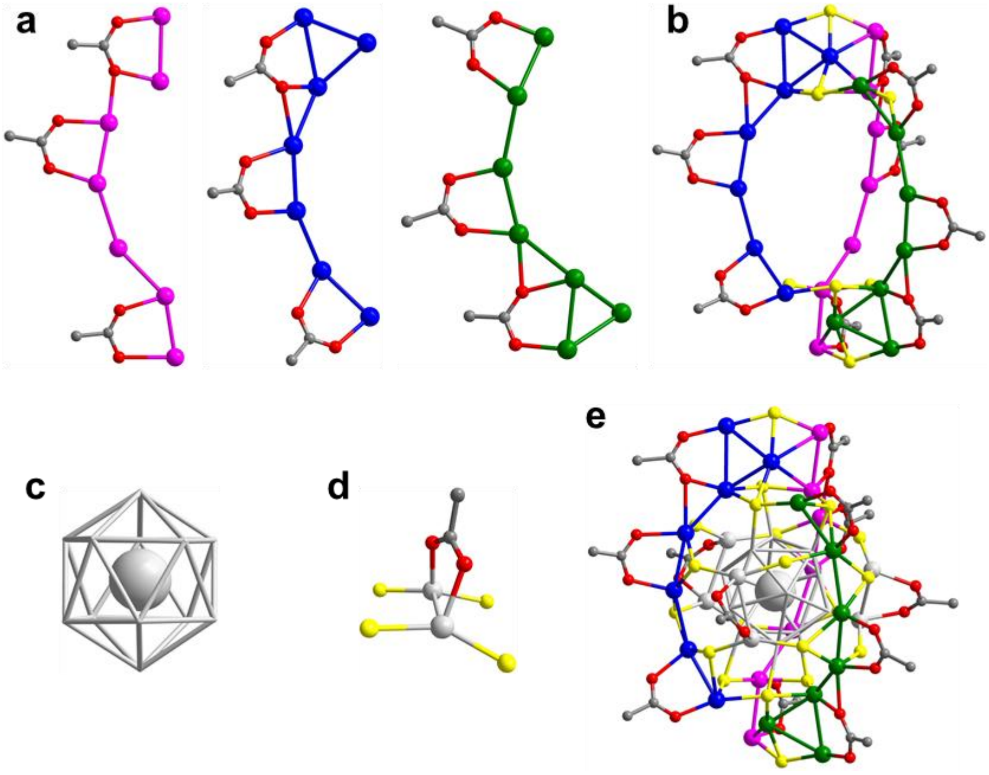

Fig. S2 Anatomy of the crystal structure of **$Ag_{40}(TBBM)_{20}(CH_3COO)_{12}$** showing. (**a**) Three $Ag_7(CH_3COO)_3$ chains on surface of Ag_{13} kernel. (**b**) Ag_{21} spiral structure formed by three Ag_7 chains of **Ag_{40}**. (**c**) Icosahedral Ag_{13} kernel. (**d**) $Ag_2(CH_3COO)(TBBM)_4$ button. (**e**) Front view for the overall structure of **$Ag_{40}(TBBM)_{20}(CH_3COO)_{12}$**. Color codes: silver/blue/green/magenta, Ag; yellow, S; red, O; gray, C. All H atoms and C atoms in TBBM are omitted for clarity.

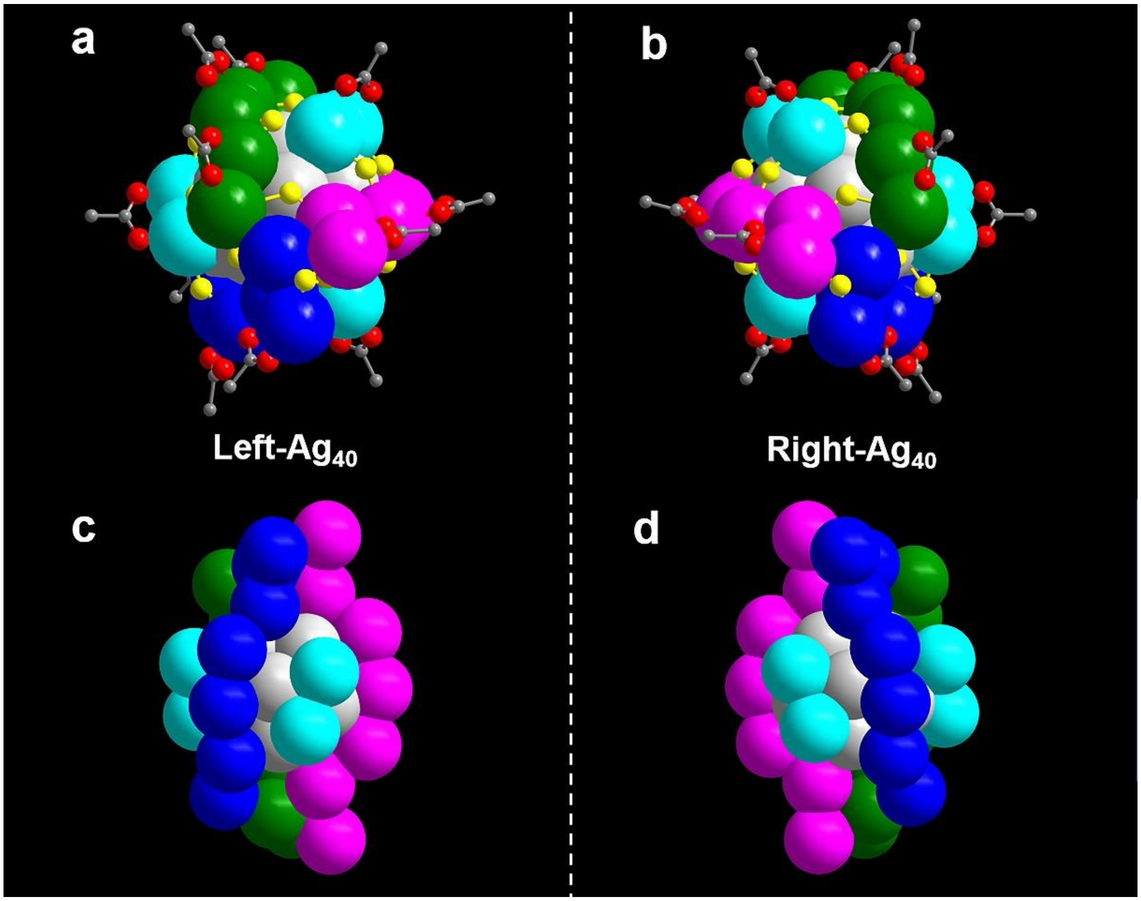

Fig. S3 (**a**) and (**b**) X-ray structures of the two **Ag_{40}** enantiomers viewed from the top, (**c**) and (**d**) arrangements of Ag_{27} viewed from the side. Color codes: silver/blue/green/turquiose/magenta, Ag; yellow, S; red, O; gray, C. All H atoms and C atoms in TBBM are omitted for clarity.

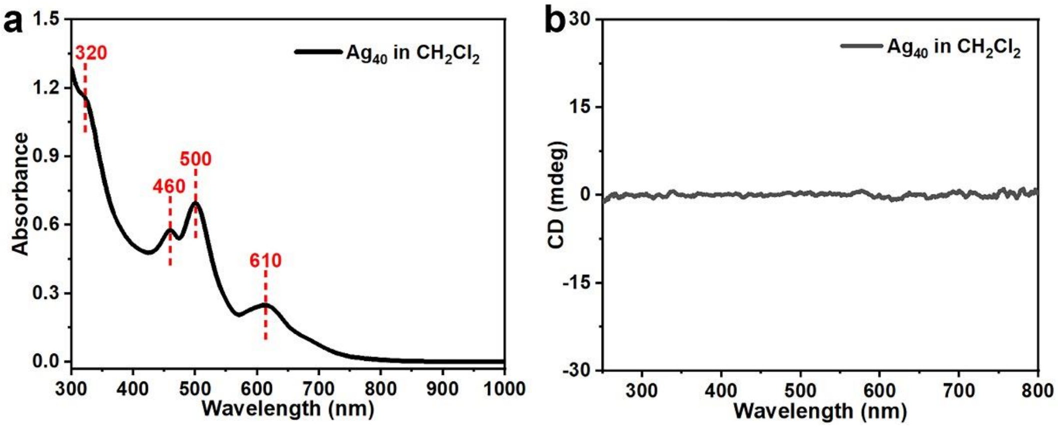

Fig. S4 (**a**) The UV-vis spectrum of **Ag_{40}** in CH_2Cl_2. (**b**) The CD spectrum of **Ag_{40}** in CH_2Cl_2.

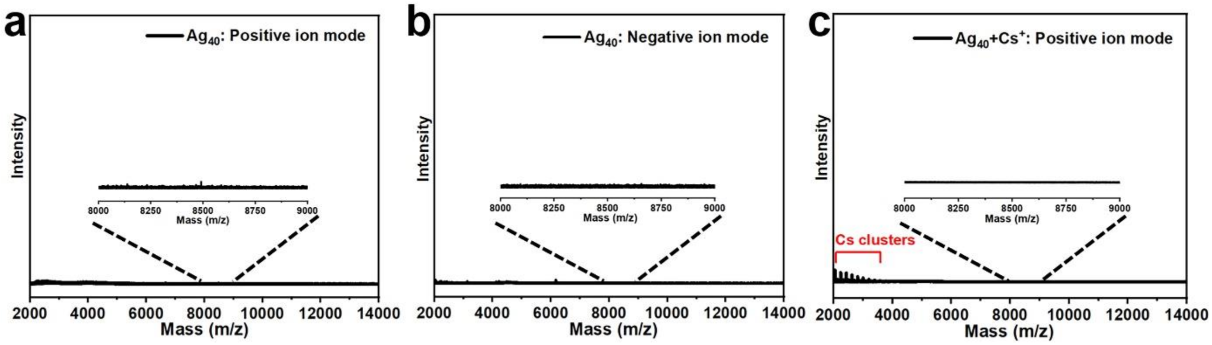

Fig. S5 ESI-MS spectra of $\mathbf{Ag_{40}}$. (**a**) Positive ion mode. (**b**) Negative ion mode. (**c**) Positive ion mode with the addition of Cs^+.

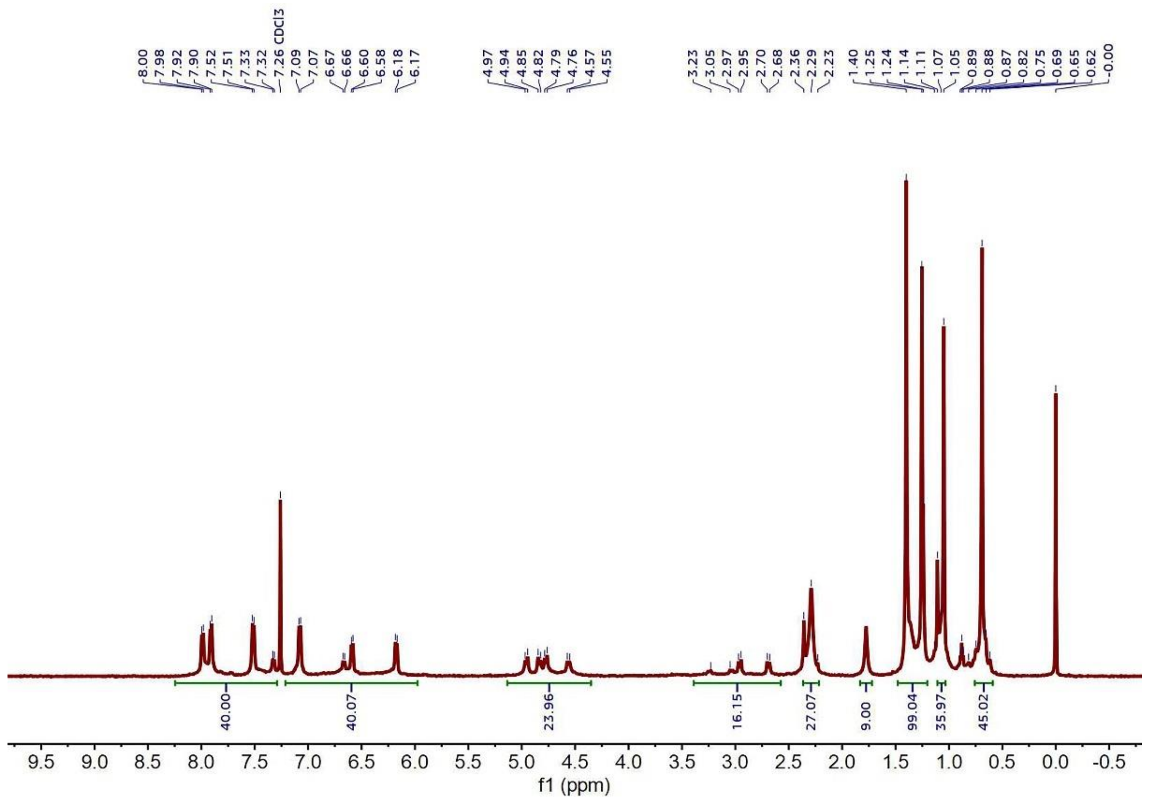

Fig. S6 ^1H-NMR spectrum of $\mathbf{Ag_{40}}$ dissolved in $CDCl_3$. The 7.26 ppm is the solvent peak of $CDCl_3$. The ^1H signals at 5.97 - 8.25 ppm (80 H = 20 × 4), 2.57 - 5.12 ppm (40 H = 20 × 2) and 0.58 - 1.48 ppm (180 H = 20 × 9) correspond to phenyl groups, benzyl groups and tertiary butyl groups in TBBM. The ^1H signals at 1.66 - 2.35 ppm (36 H = 12 × 3) correspond to the CH_3COO^-.

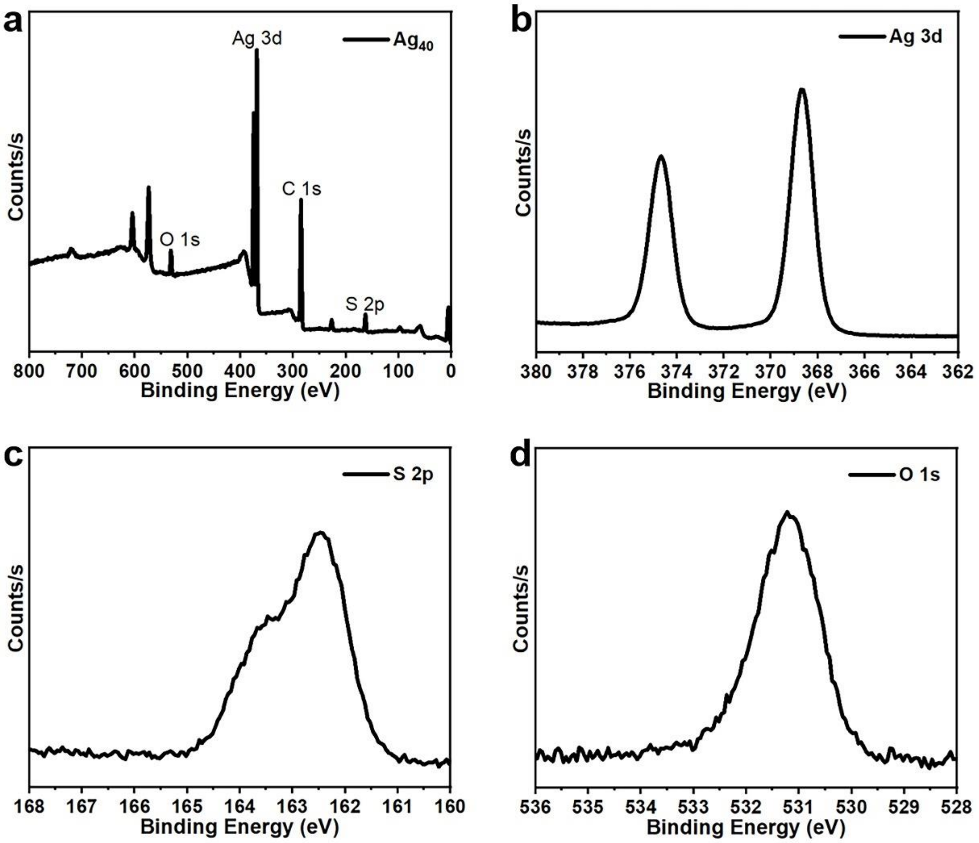

Fig. S7 The XPS data of **Ag_{40}**. (**a**) Survey spectrum. (**b**) Ag 3d. (**c**) S 2p. (**d**) O 1s.

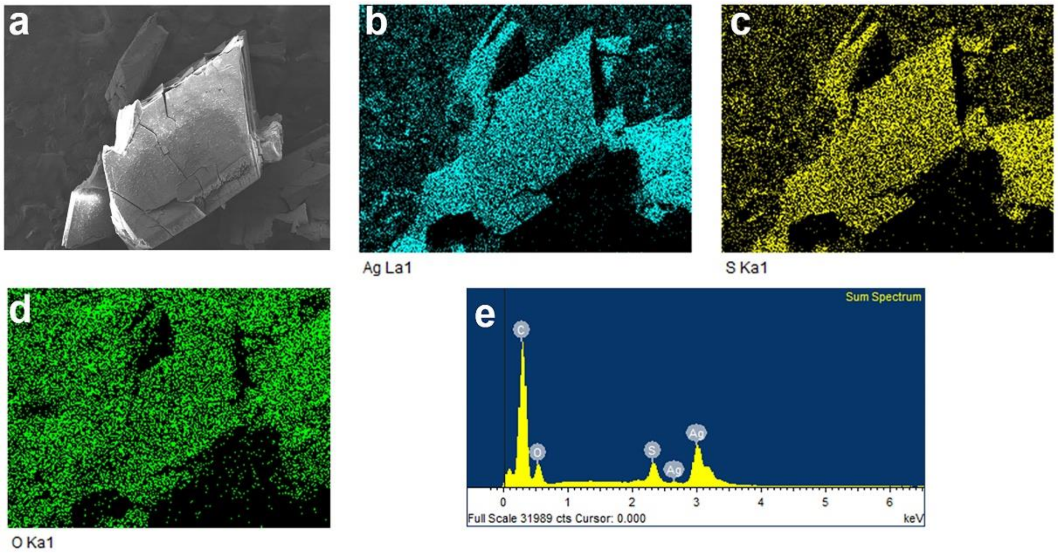

Fig. S8 (**a**) SEM image of a small (deformed) single crystal of **Ag_{40}**. (**b**)-(**d**) Elemental mapping images of Ag, S and O, respectively. (**e**) EDS spectrum confirming the presence of above elements in **Ag_{40}** cluster, which is consistent with the cluster composition obtained by SC-XRD.

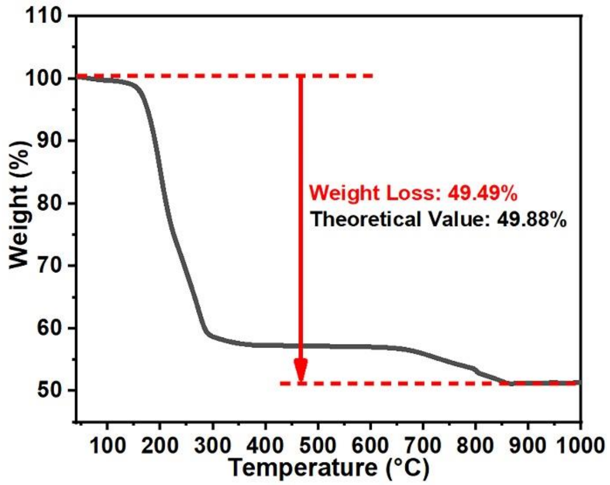

Fig. S9 TGA analysis of $\mathbf{Ag_{40}}$.

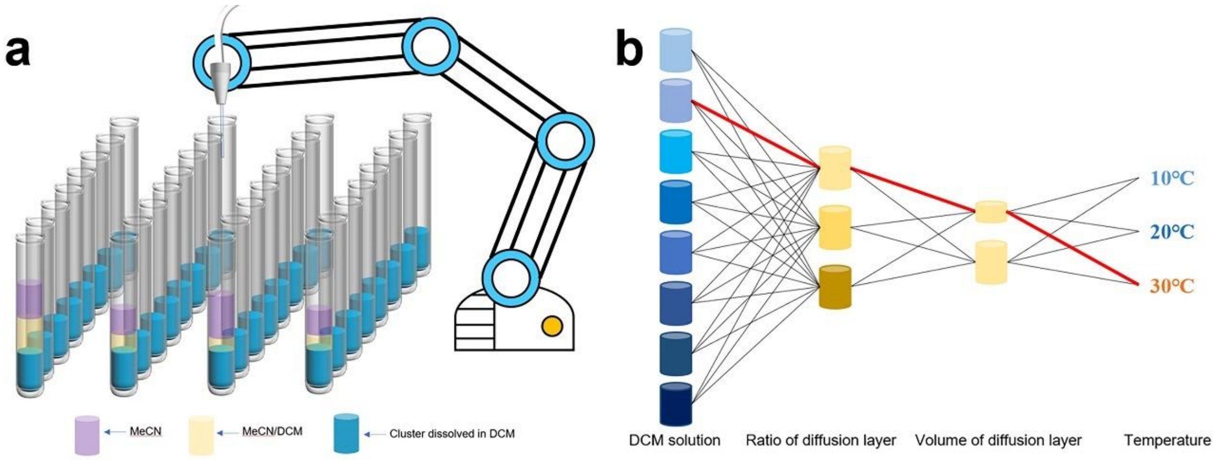

Fig. S10 Automatic crystallization system and crystallization conditions. (**a**) Schematic diagram of an automatic crystallization system. (**b**) Schematic diagram of recrystallization condition. The concentrations of Ag_{40} in DCM solutions are 3, 4, 5, 6, 7, 8, 9 and 10 mg/mL. The ratios of DCM and CH_3CN in the diffusion layer are 1 : 1, 1 : 2 and 1 : 3. The volume ratios of DCM solution to diffusion layer solution is equal to 1 : 0.5 and 1 : 1. The three temperature conditions are 10, 20 and 30 ℃.

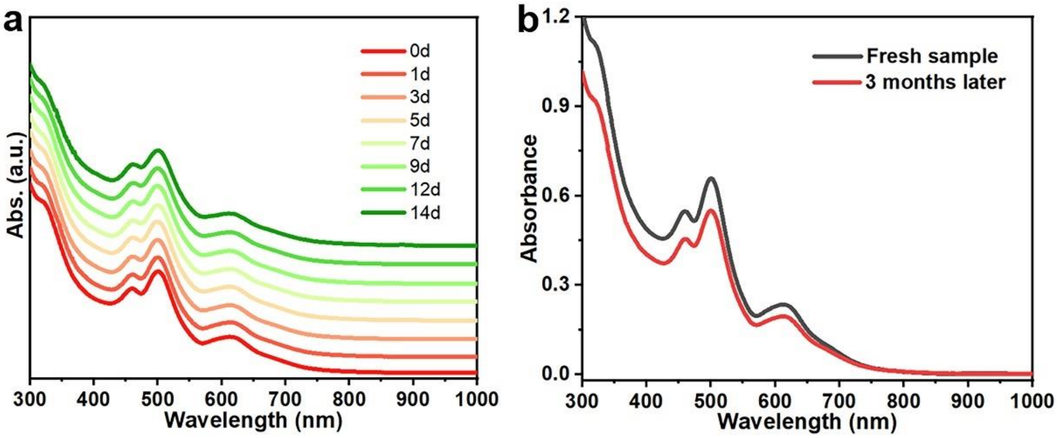

Fig. S11 Monitoring the stability of $\mathbf{Ag_{40}}$. (**a**) Time-dependent UV-vis spectra of $\mathbf{Ag_{40}}$ in the CH_2Cl_2 solution. (**b**) Time-dependent UV-vis spectra of $\mathbf{Ag_{40}}$ in the solid state.

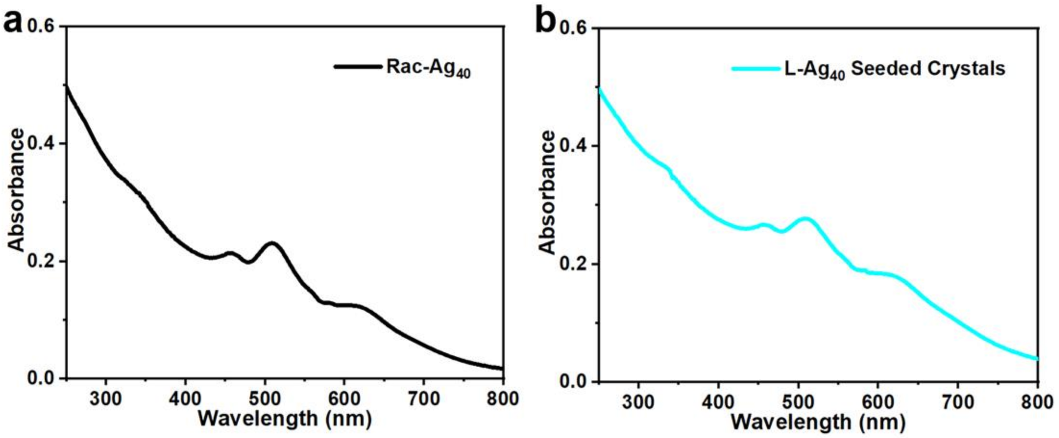

Fig. S12 UV-vis spectra of $\mathbf{Ag_{40}}$ crystals in solid state. (**a**) **Rac-**$\mathbf{Ag_{40}}$. **(b) L-**$\mathbf{Ag_{40}}$ seeded crystals.

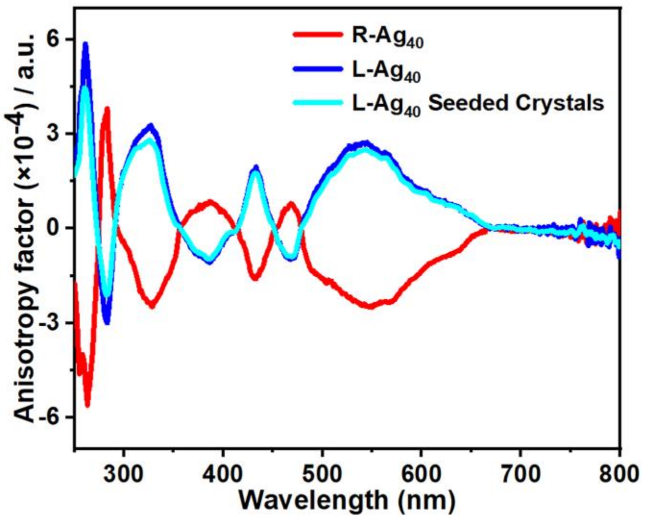

Fig. S13 Anisotropy factors of **R-**$\mathbf{Ag_{40}}$ (red), **L-**$\mathbf{Ag_{40}}$ (blue) and **L-**$\mathbf{Ag_{40}}$ seeded crystals (cyan).

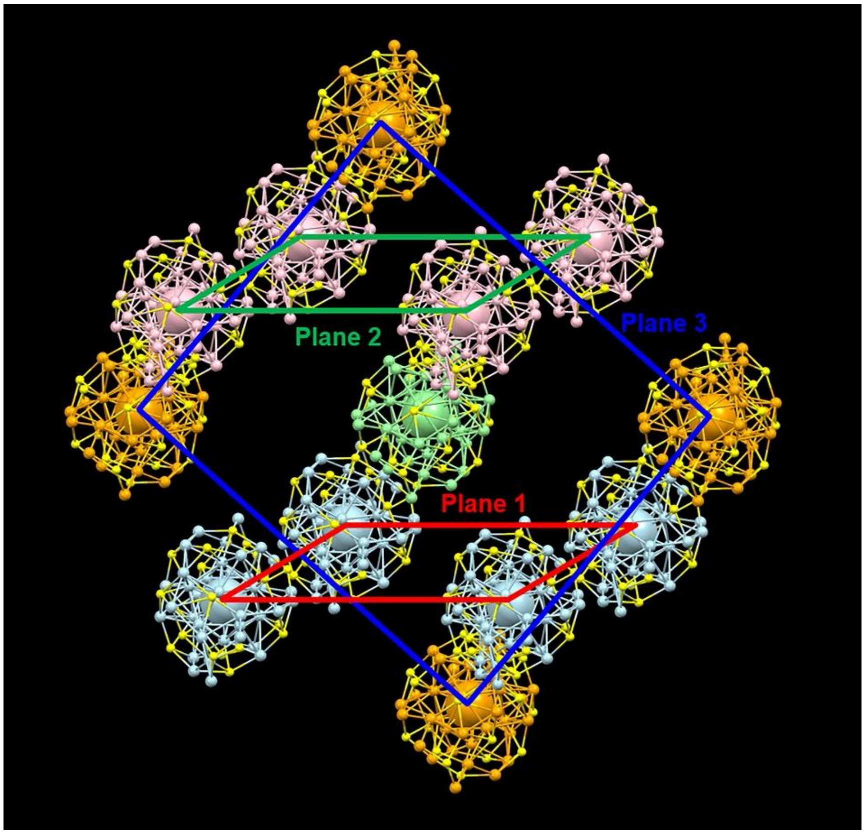

Fig. S14 Illustration of twelve **Ag_{40}** molecules surrounding one **Ag_{40}** molecule (central molecule, Ag = light blue) and the twelve **Ag_{40}** molecules are divided into three planes (Plane 1, Plane 2 and Plane 3) in racemic **Ag_{40}** crystals. Color codes: pink/orange/light green/light blue, Ag; yellow, S. All H, O and C atoms are omitted for clarity.

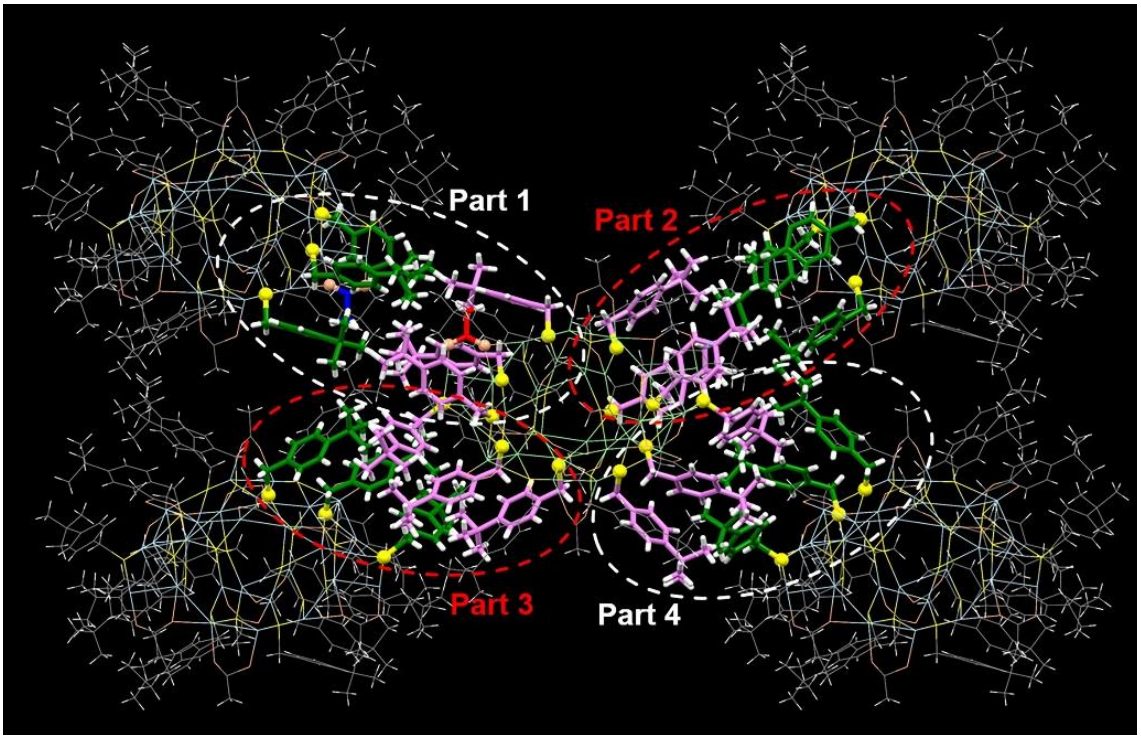

Fig. S15 Illustration of the steric-hindrance effects between the four neighbouring **Ag_{40}** molecules in Plane 1 and the one central **Ag_{40}** molecule along the b axis. Color codes: light blue/light green, Ag; yellow, S; light orange, O; green/blue, C in the four neighbouring **Ag_{40}** molecules in Plane 1; violet/red, C in the central **Ag_{40}** molecule; green and violet C atoms in TBBM; blue and red C atoms in CH_3COO. All Ag_{13} kernels are omitted for clarity.

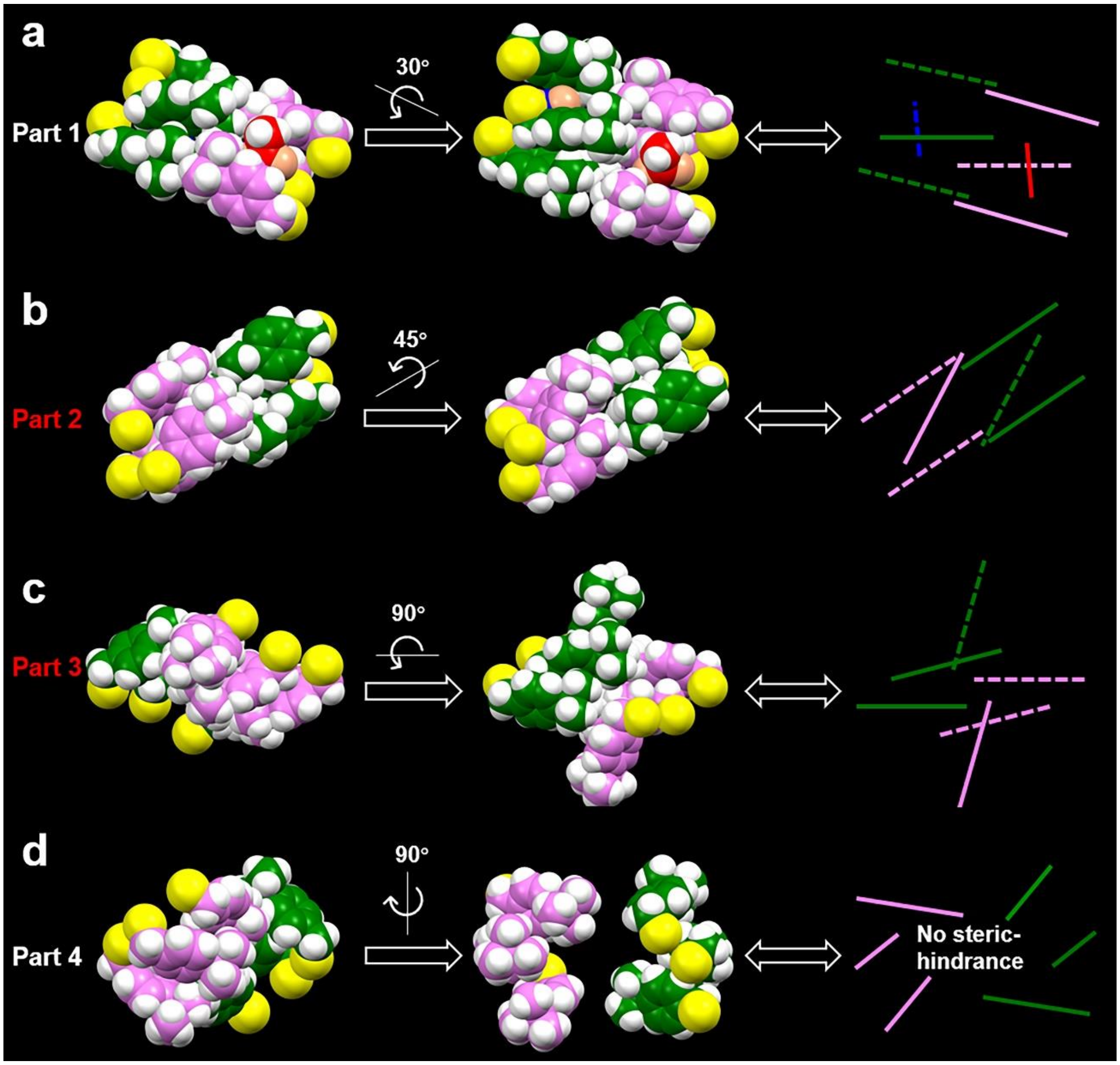

Fig. S16 Illustration is the enlarged section circled in Fig. S15. Color codes: yellow, S; light orange, O; green/blue, C in the four neighbouring **Ag_{40}** molecules in Plane 1; violet/red, C in the central **Ag_{40}** molecule; green and violet C atoms in TBBM; blue and red C atoms in CH_3COO.

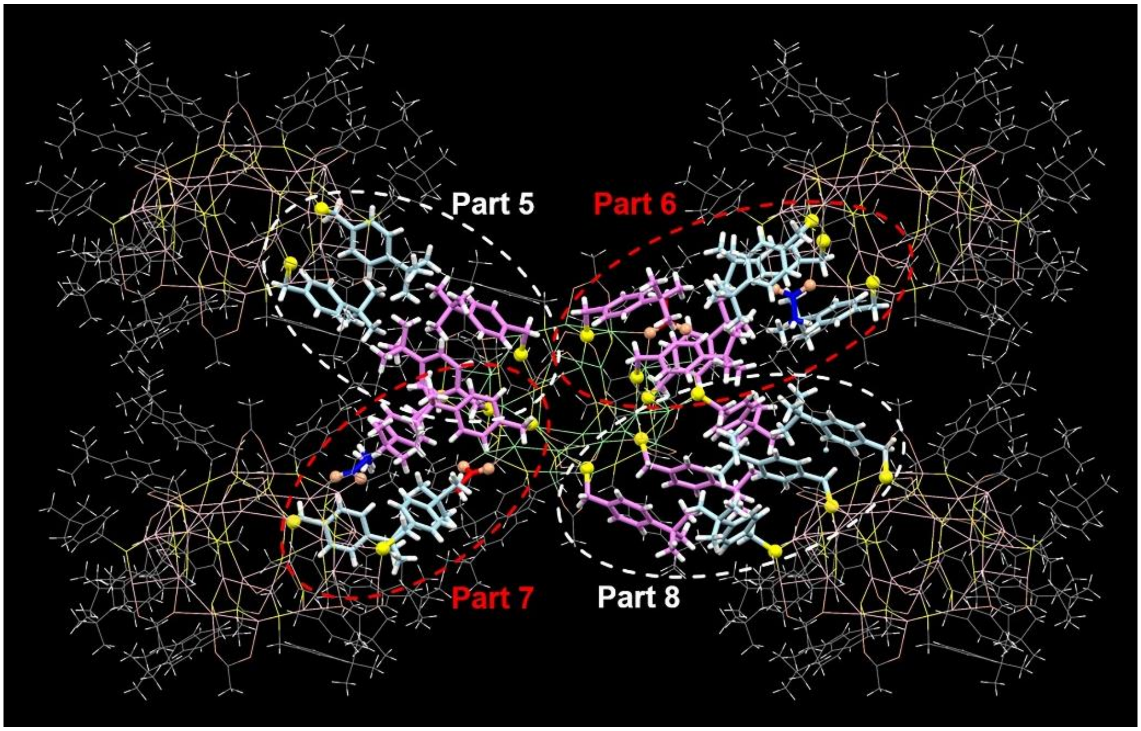

Fig. S17 Illustration of the steric-hindrance effects between the four neighbouring $\mathbf{Ag_{40}}$ molecules in Plane 2 and the one central $\mathbf{Ag_{40}}$ molecule along the b axis. Color codes: pink/light green, Ag; yellow, S; light orange, O; light blue/blue, C in the four neighbouring $\mathbf{Ag_{40}}$ molecules in Plane 2; violet/red, C in the central $\mathbf{Ag_{40}}$ molecule; light blue and violet C atoms in TBBM; blue and red C atoms in CH_3COO. All Ag_{13} kernels are omitted for clarity.

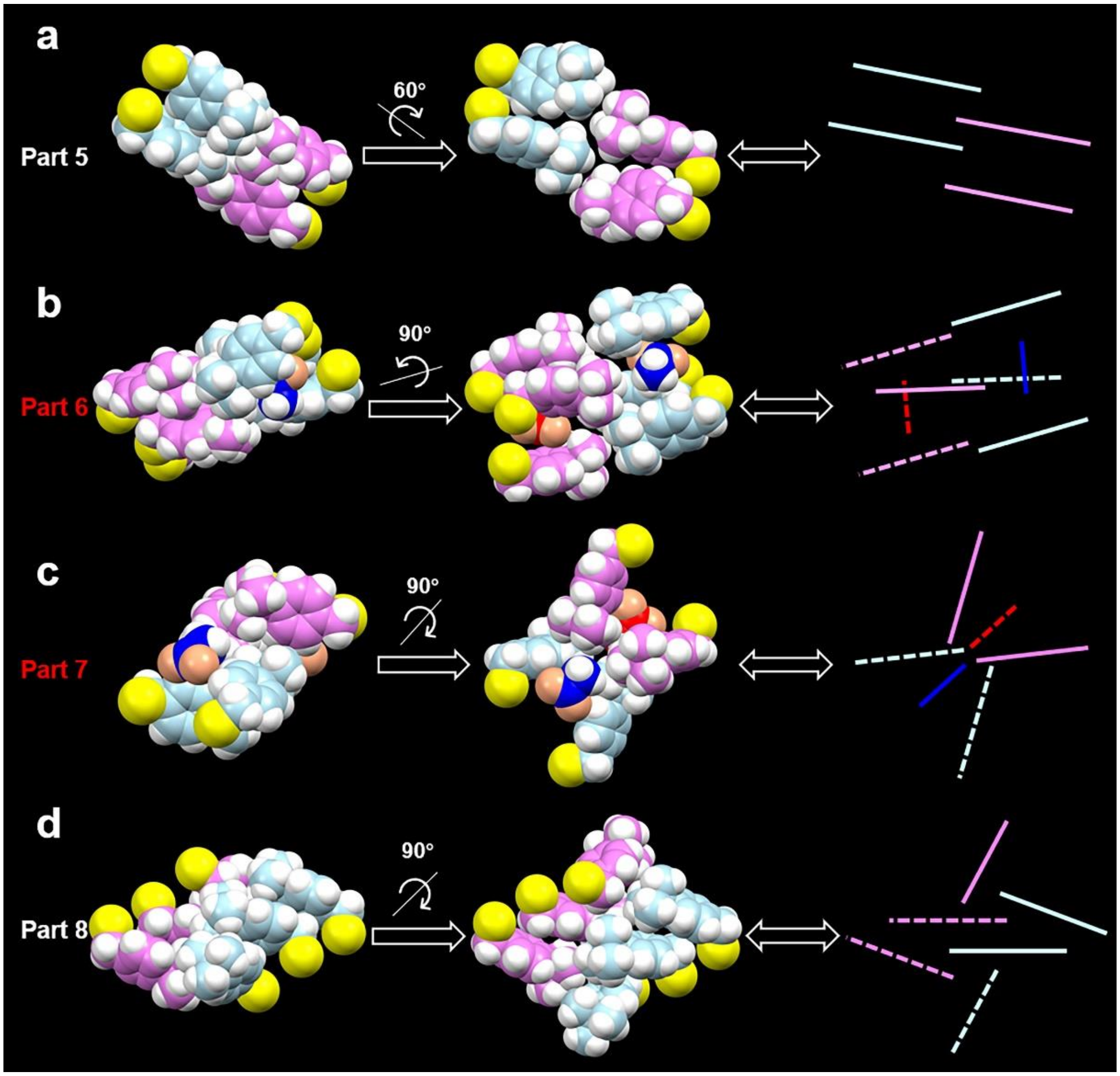

Fig. S18 Illustration is the enlarged section circled in Fig. S17. Color codes: yellow, S; light orange, O; light blue/blue, C in the four neighbouring **Ag_{40}** molecules in Plane 2; violet/red, C in the central **Ag_{40}** molecule; light blue and violet C atoms in TBBM; blue and red C atoms in CH_3COO.

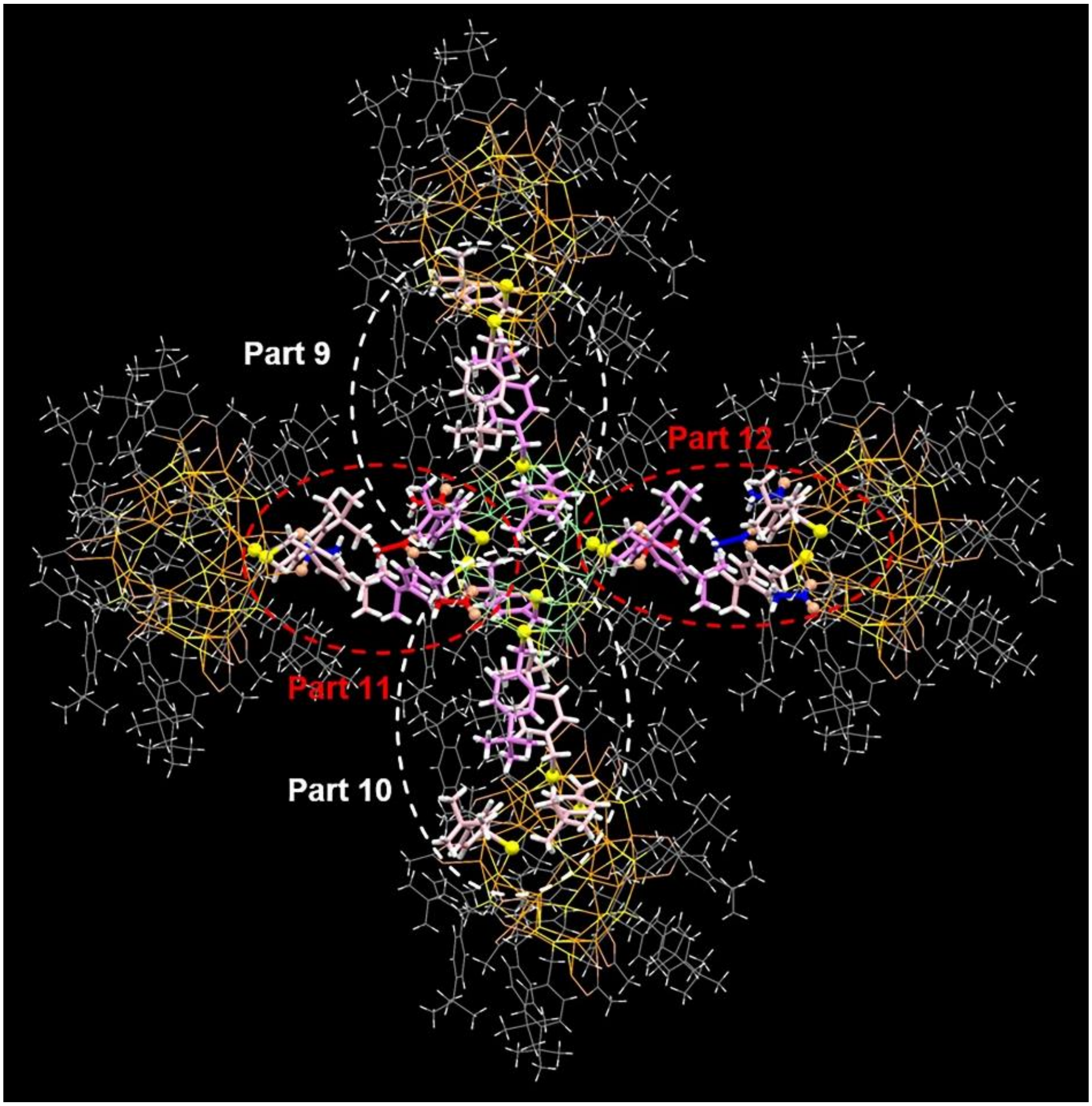

Fig. S19 Illustration of the steric-hindrance effects between the four neighbouring **Ag_{40}** molecules in Plane 3 and the one central **Ag_{40}** molecule along the c axis. Color codes: orange/light green, Ag; yellow, S; light orange, O; pink/blue, C in the four neighbouring **Ag_{40}** molecules in Plane 3; violet/red, C in the central **Ag_{40}** molecule; pink and violet C atoms in TBBM; blue and red C atoms in CH_3COO. All Ag_{13} kernels are omitted for clarity.

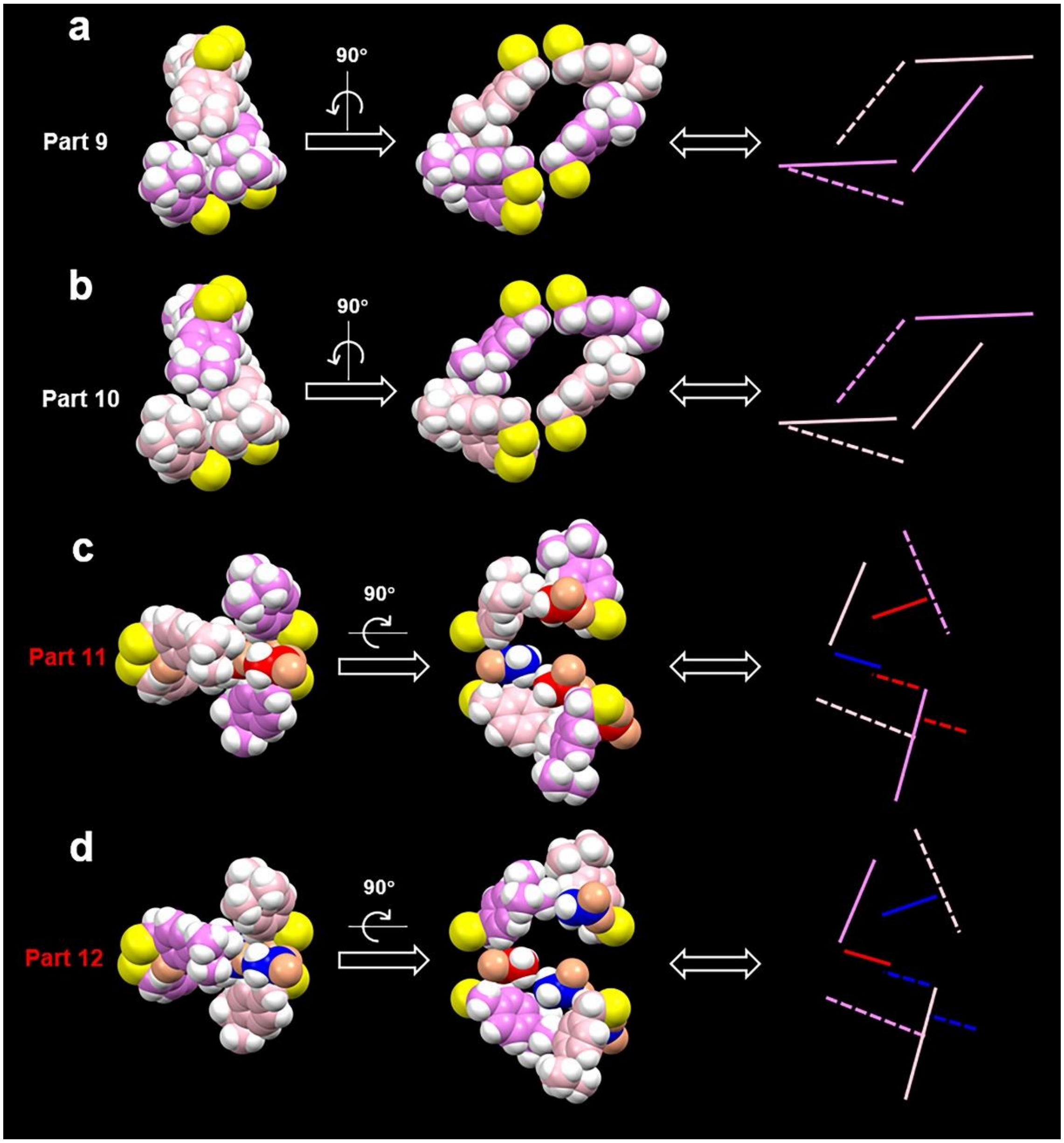

Fig. S20 Illustration is the enlarged section circled in Fig. S19. Color codes: yellow, S; light orange, O; pink/blue, C in the four neighbouring **Ag_{40}** molecules in Plane 3; violet/red, C in the central **Ag_{40}** molecule; pink and violet C atoms in TBBM; blue and red C atoms in CH_3COO.

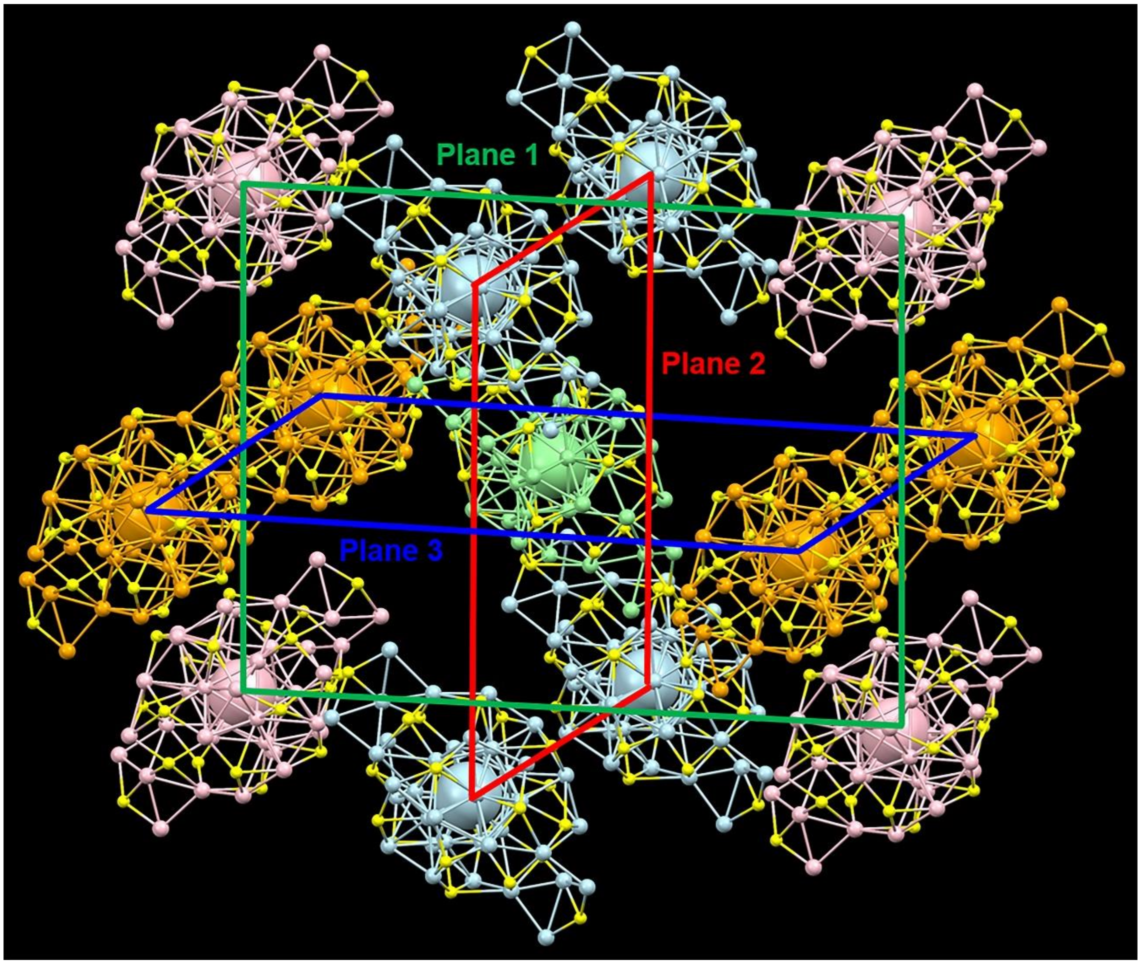

Fig. S21 Illustration of twelve **Ag_{40}** molecules surrounding one **Ag_{40}** molecule (central molecule, Ag = light blue) and the twelve **Ag_{40}** molecules are divided into three planes (Plane 1, Plane 2 and Plane 3) in homochiral **Ag_{40}** crystals. Color codes: pink/orange/light green/light blue, Ag; yellow, S. All H, O and C atoms are omitted for clarity.

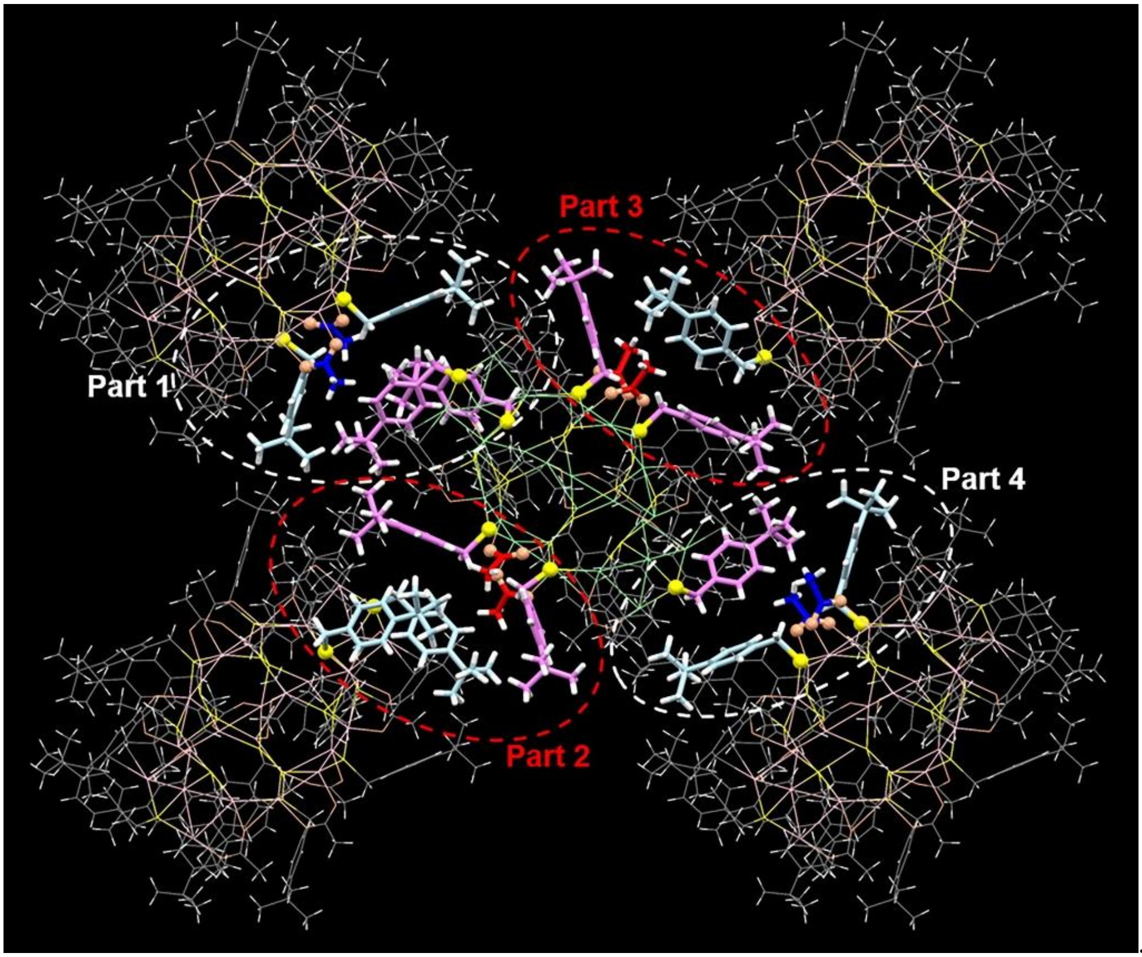

Fig. S22 Illustration of the steric-hindrance effects between the four neighbouring **Ag_{40}** molecules in Plane 1 and the one central **Ag_{40}** molecule along the b axis. Color codes: pink/light green,Ag; yellow, S; light orange, O; light blue/blue, C in the four neighbouring **Ag_{40}** molecules in Plane 1; violet/red, C in the central **Ag_{40}** molecule; light blue and violet C atoms in TBBM; blue and red C atoms in CH_3COO. All Ag_{13} kernels are omitted for clarity.

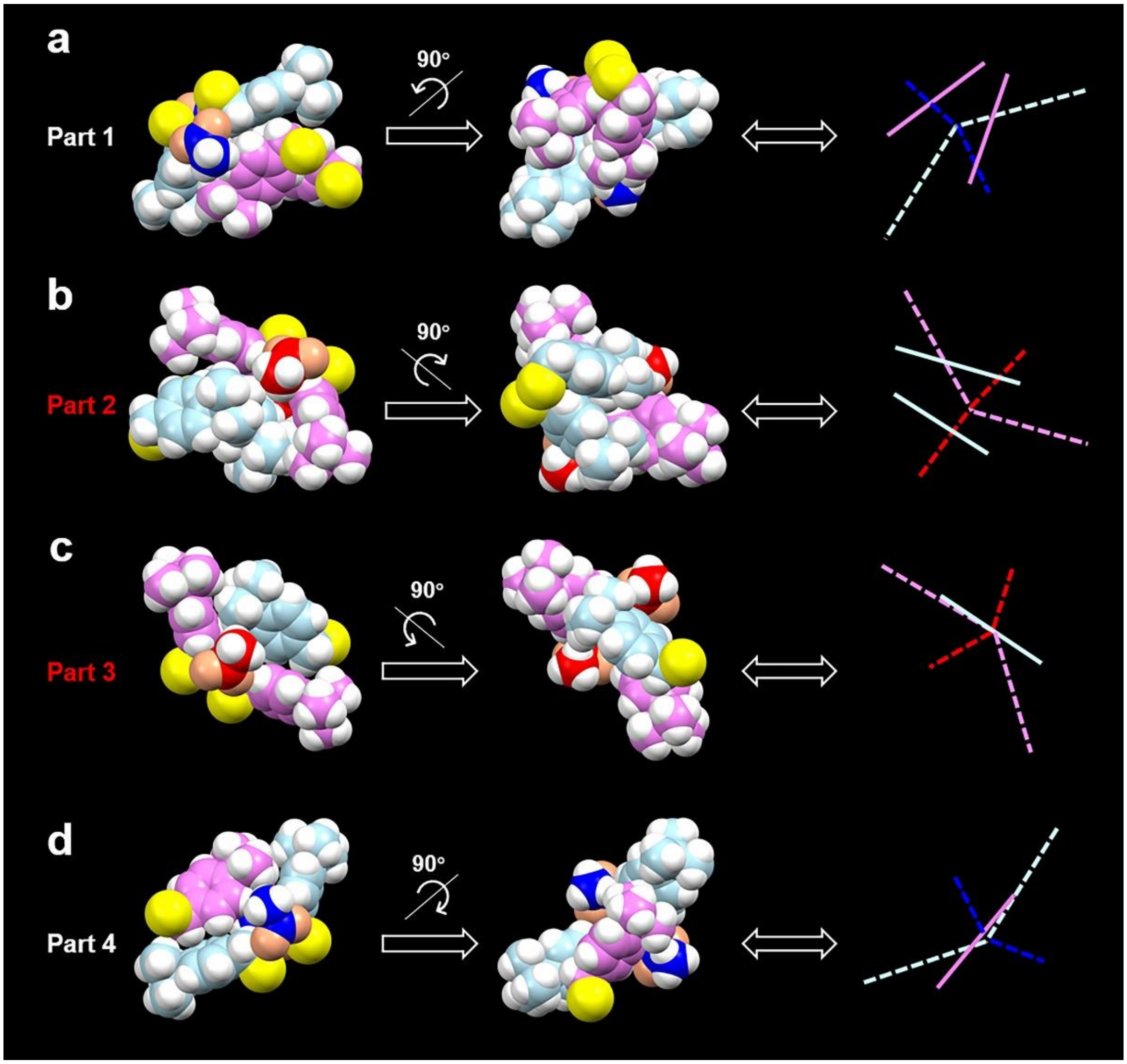

Fig. S23 Illustration is the enlarged section circled in Fig. S22. Color codes: yellow, S; light orange, O; light blue/blue, C in the four neighbouring **Ag_{40}** molecules in Plane 2; violet/red, C in the central **Ag_{40}** molecule; light blue and violet C atoms in TBBM; blue and red C atoms in CH_3COO.

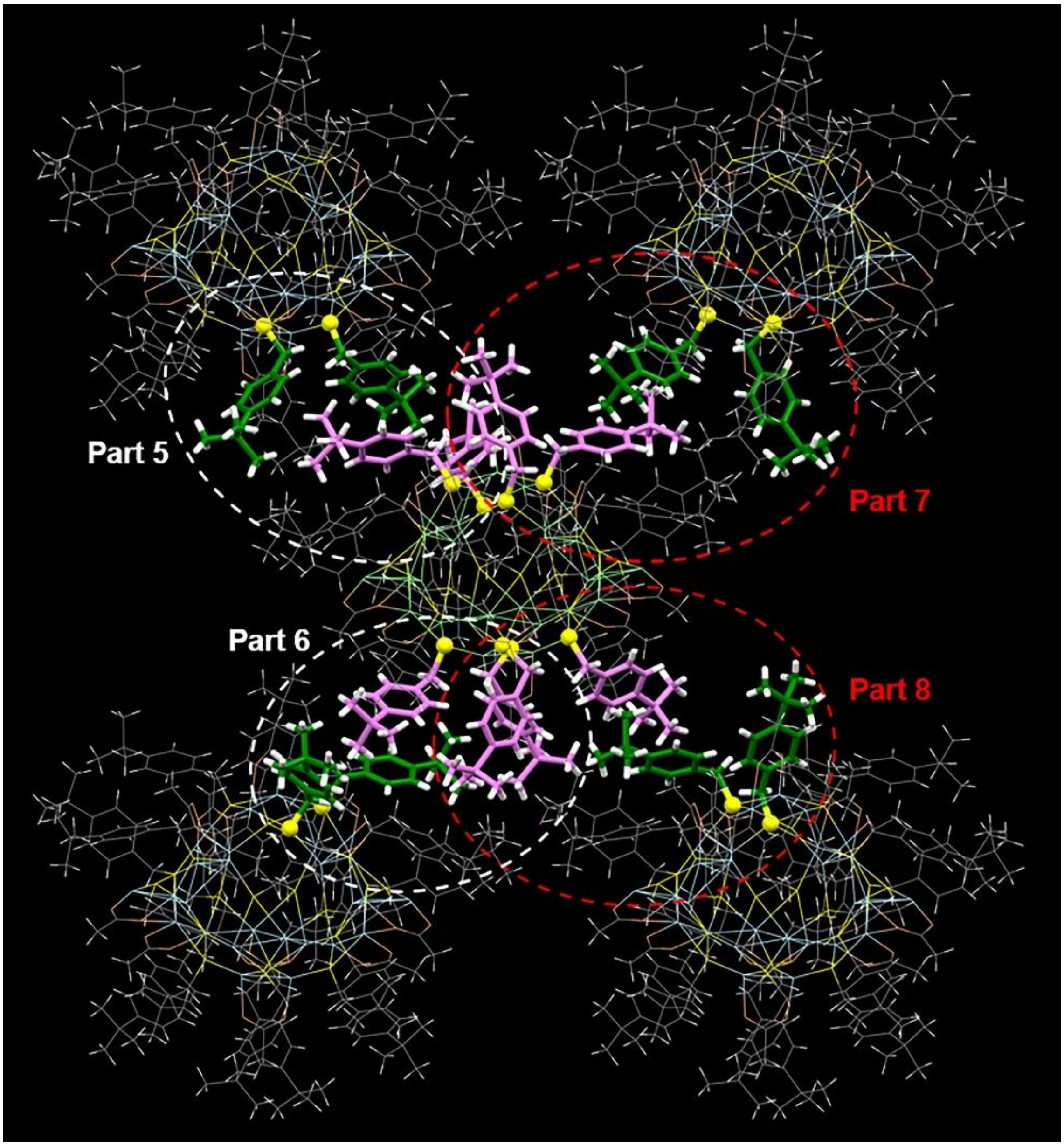

Fig. S24 Illustration of the steric-hindrance effects between the four neighbouring **Ag_{40}** molecules in Plane 2 and the one central **Ag_{40}** molecule along the c axis. Color codes: light blue/light green, Ag; yellow, S; light orange, O; green, C in the four neighbouring **Ag_{40}** molecules in Plane 2; violet, C in the central **Ag_{40}** molecule. All Ag_{13} kernels are omitted for clarity.

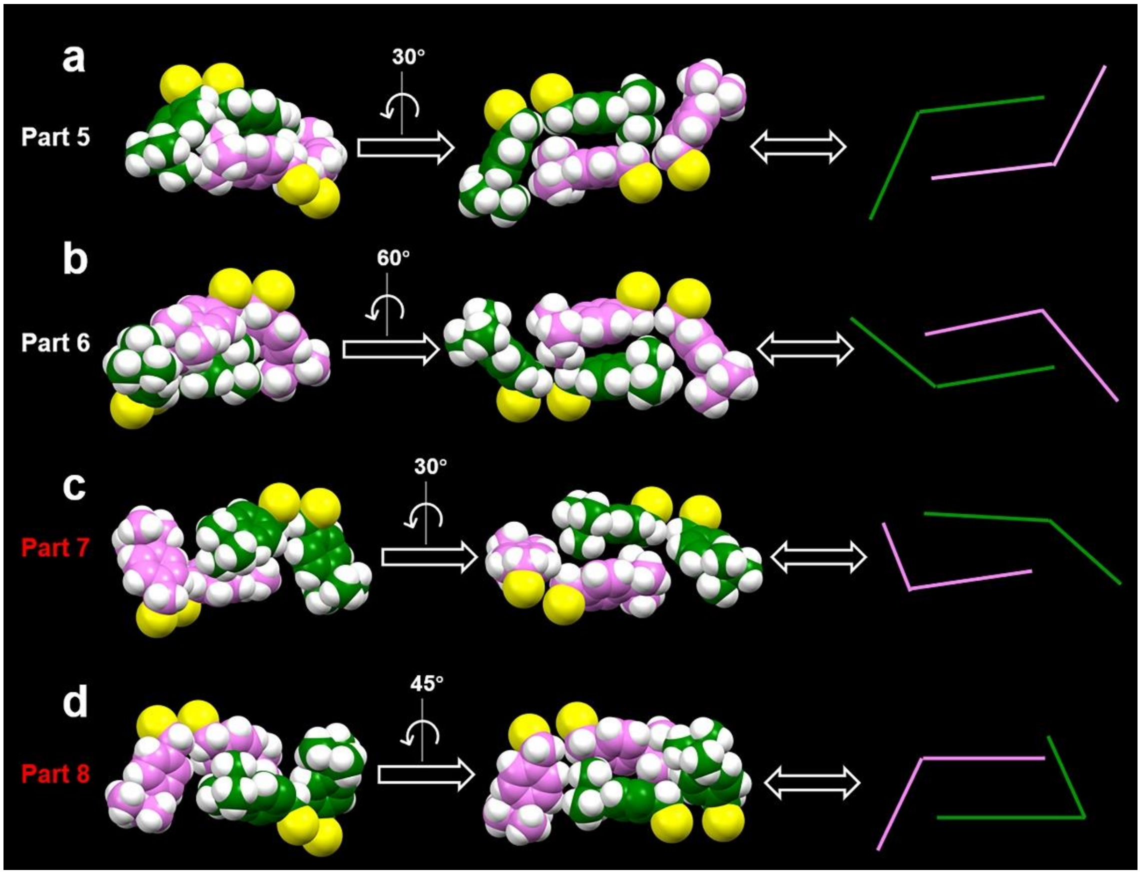

Fig. S25 Illustration is the enlarged section circled in Fig. S24. Color codes: yellow, S; light orange, O; green, C in the four neighbouring $\mathbf{Ag_{40}}$ molecules in Plane 2; violet, C in the central $\mathbf{Ag_{40}}$ molecule.

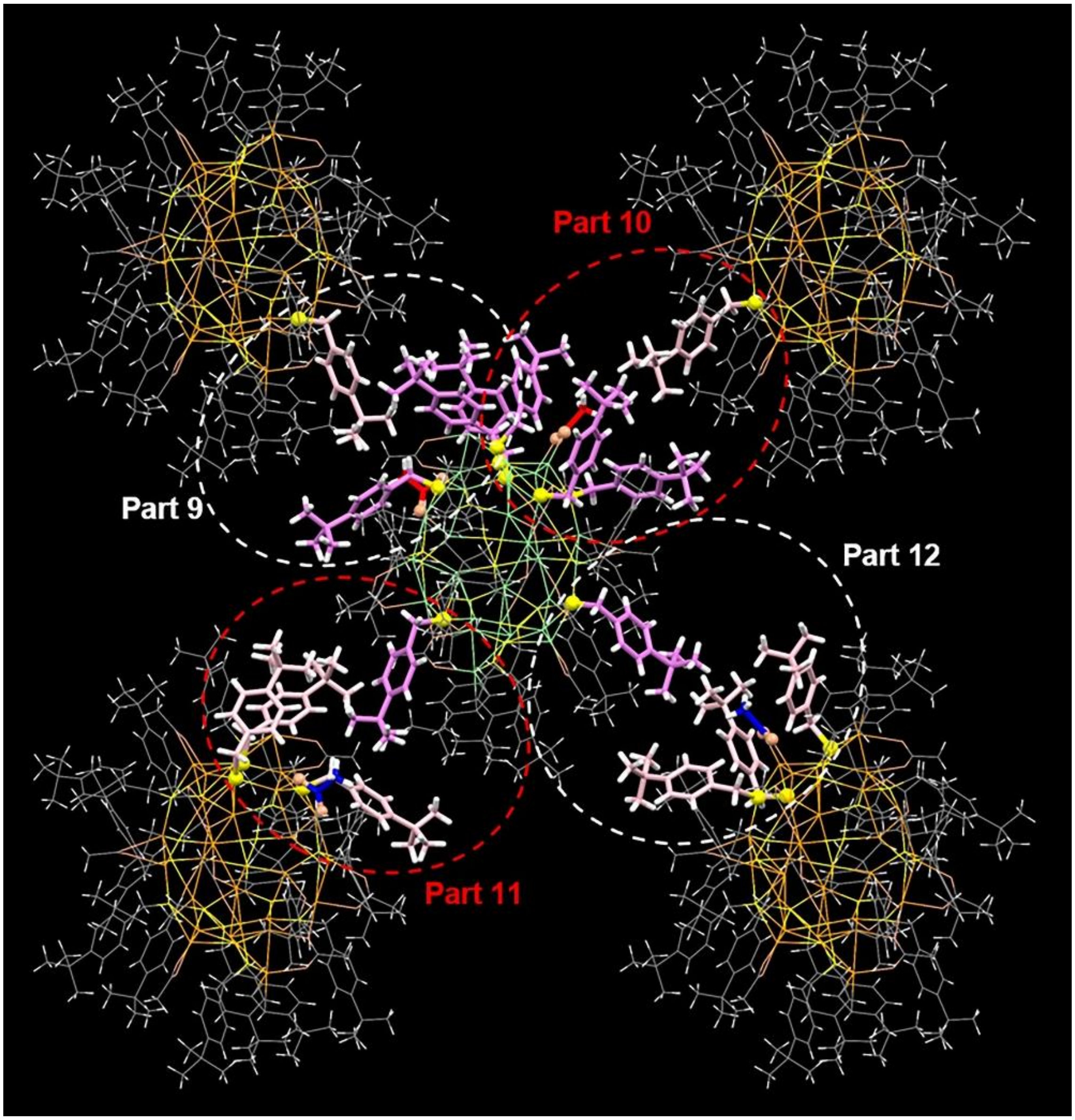

Fig. S26 Illustration of the steric-hindrance effects between the four neighbouring **Ag_{40}** molecules in Plane 3 and the one central **Ag_{40}** molecule along the a axis. Color codes: orange/light green, Ag; yellow, S; light orange, O; pink/blue, C in the four neighbouring **Ag_{40}** molecules in Plane 3; violet/red, C in the central **Ag_{40}** molecule; pink and violet C atoms in TBBM; blue and red C atoms in CH_3COO. All Ag_{13} kernels are omitted for clarity.

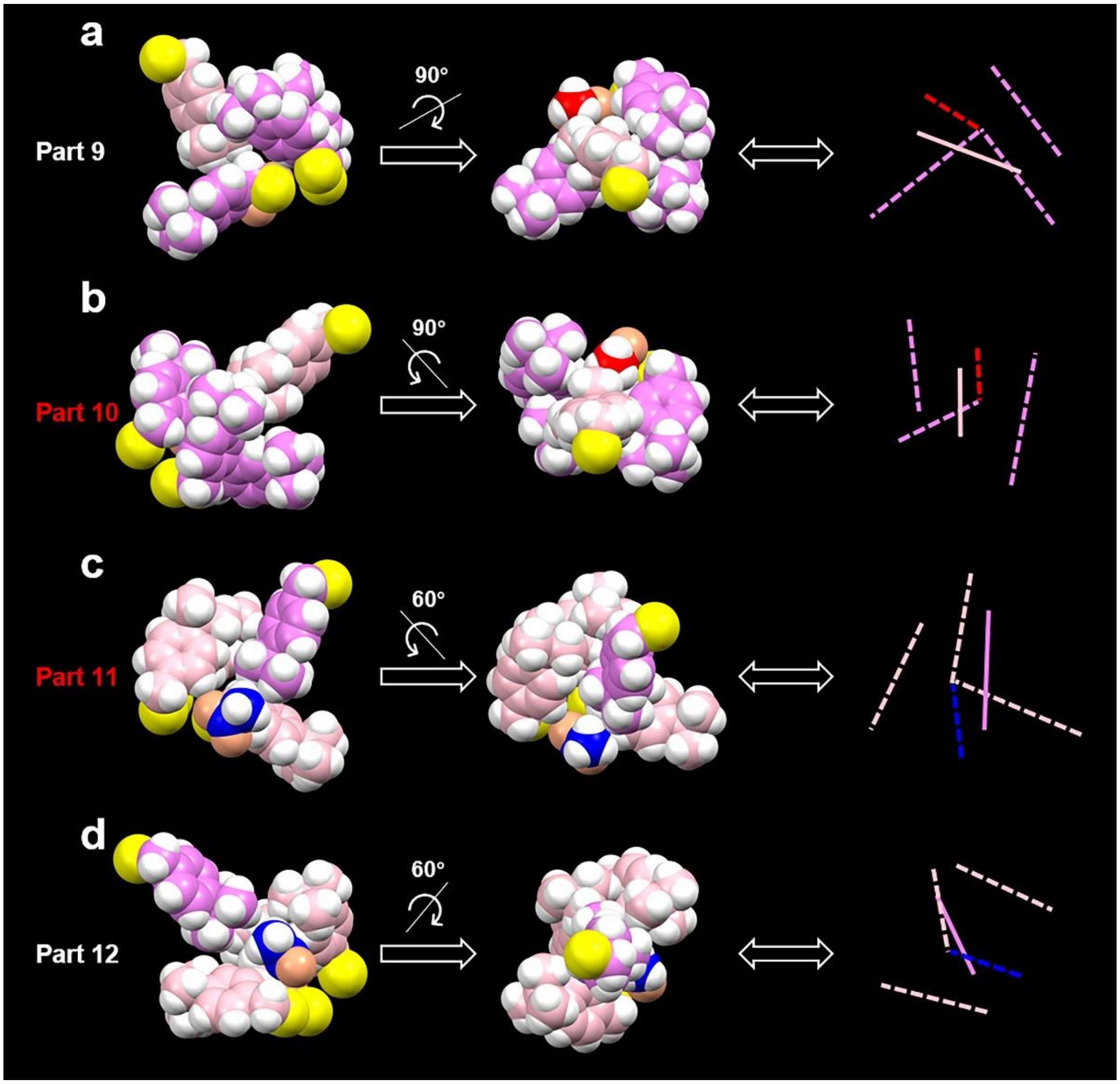

Fig. S27 Illustration is the enlarged section circled in Fig. S26. Color codes: yellow, S; light orange, O; pink/blue, C in the four neighbouring **Ag_{40}** molecules in Plane 3; violet/red, C in the central **Ag_{40}** molecule; pink and violet C atoms in TBBM; blue and red C atoms in CH_3COO.

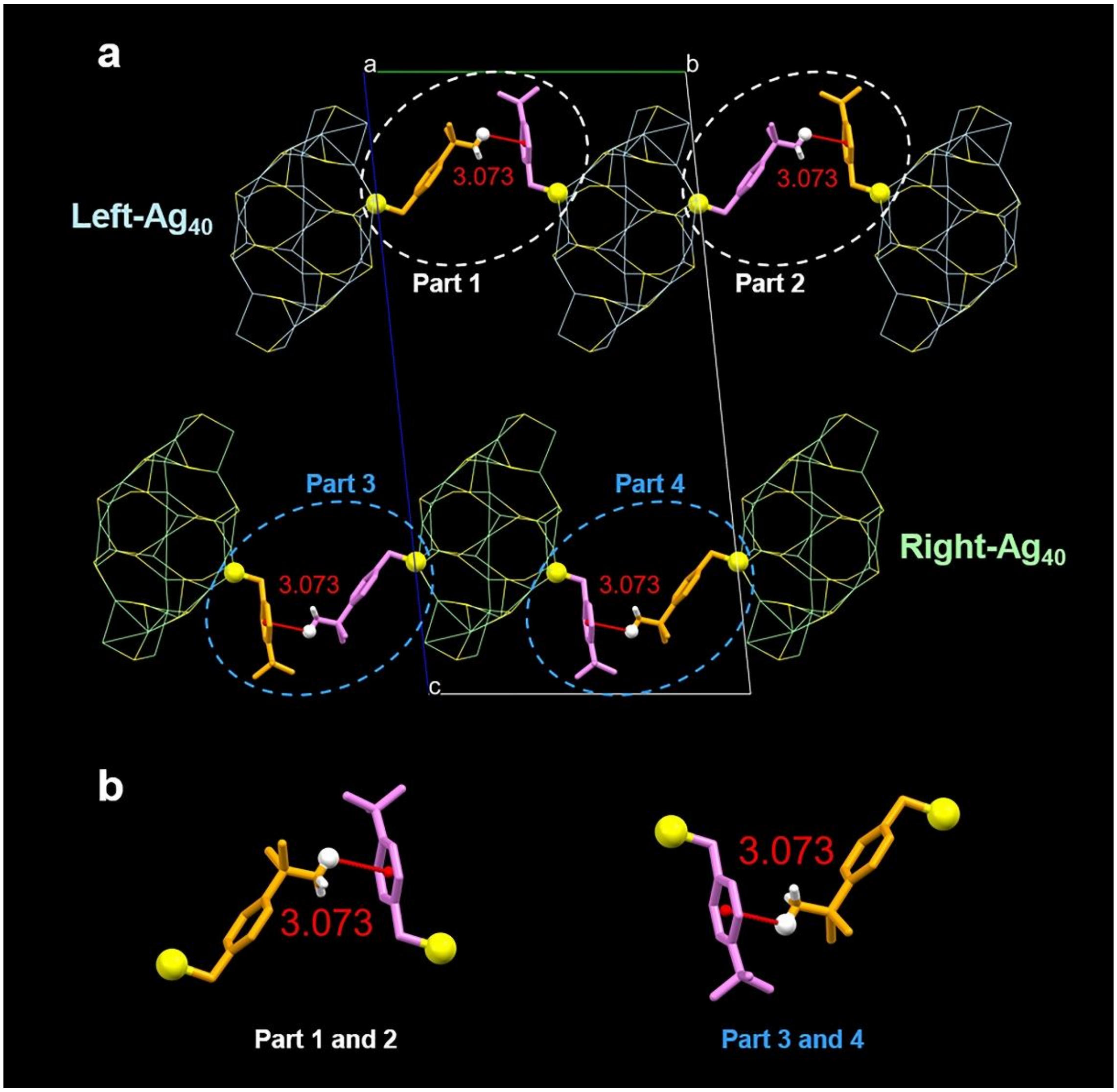

Fig. S28 (**a**) Illustration of the intercluster C-H···π interactions in the racemic **Ag_{40}** crystals along the a axis. (**b**) is the enlarged section circled in (**a**). Color codes: light green/light blue, Ag; yellow, S; violet/orange, C; white, H; red, centroid of benzene ring. The red numerical value and dashed lines correspond to the measured distances of C-H···π interactions. Ag_{13} kernel, partial H, C atoms and all O atoms are omitted for clarity.

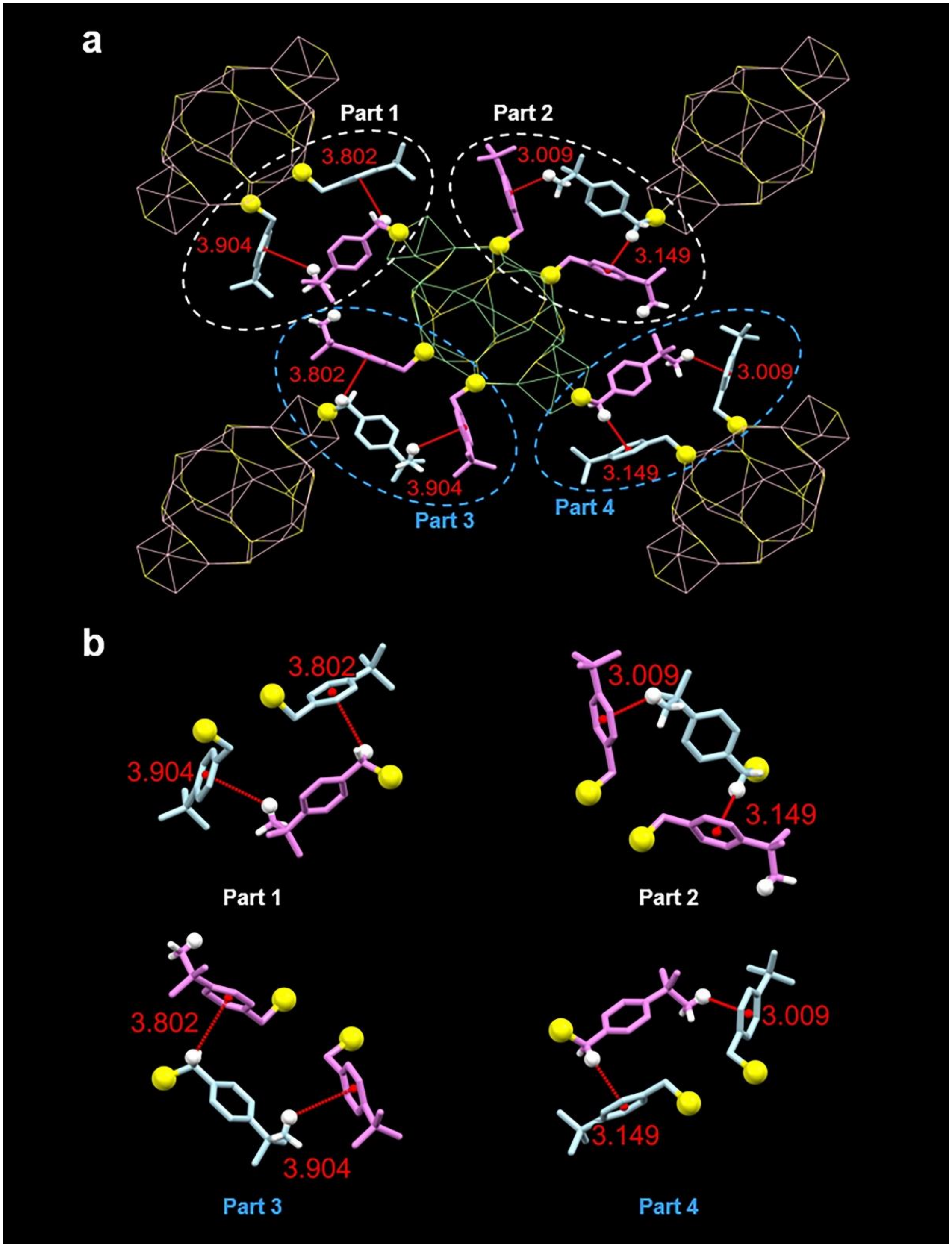

Fig. S29 (**a**) Illustration of the intercluster C-H···π interactions in the homochiral **Ag_{40}** crystals in Plane 1 of Fig. S21, along the b axis. (**b**) is the enlarged section circled in (**a**). Color codes: pink/light green, Ag; yellow, S; light blue/violet, C; white, H; red, centroid of benzene ring. The red numerical value and dashed lines correspond to the measured distances of C-H···π interactions. Ag_{13} kernel, partial H, C atoms and all O atoms are omitted for clarity.

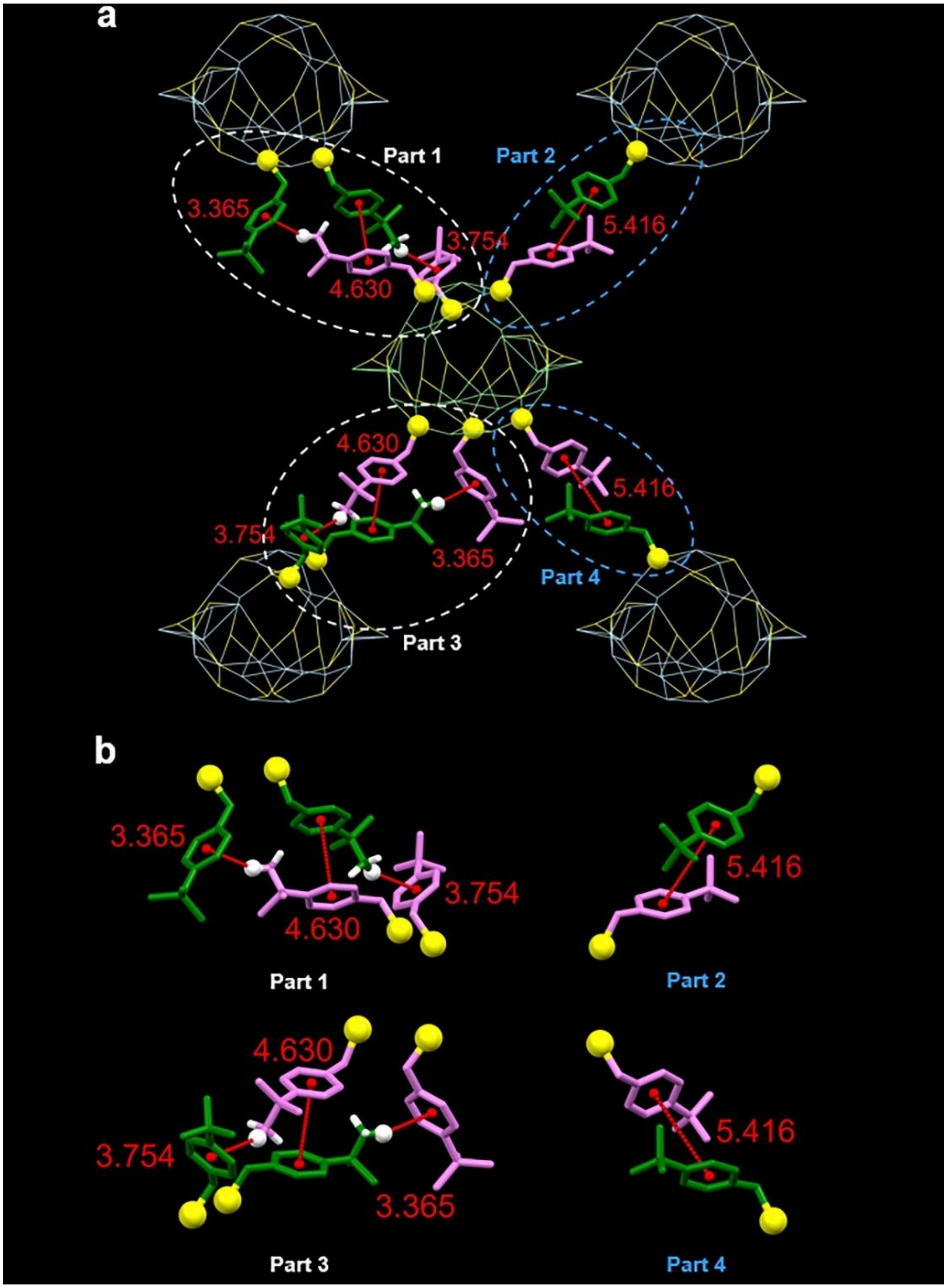

Fig. S30 (**a**) Illustration of the intercluster C-H···π and π···π interactions in the homochiral $\mathbf{Ag_{40}}$ crystals in Plane 2 of Fig. S21, along the c axis. (**b**) is the enlarged section circled in (**a**). Color codes: light blue/light green, Ag; yellow, S; violet/green, C; white, H; red, centroid of benzene ring. The red numerical value and dashed lines correspond to the measured distances of C-H···π interactions. Ag_{13} kernel, partial H, C atoms and all O atoms are omitted for clarity.

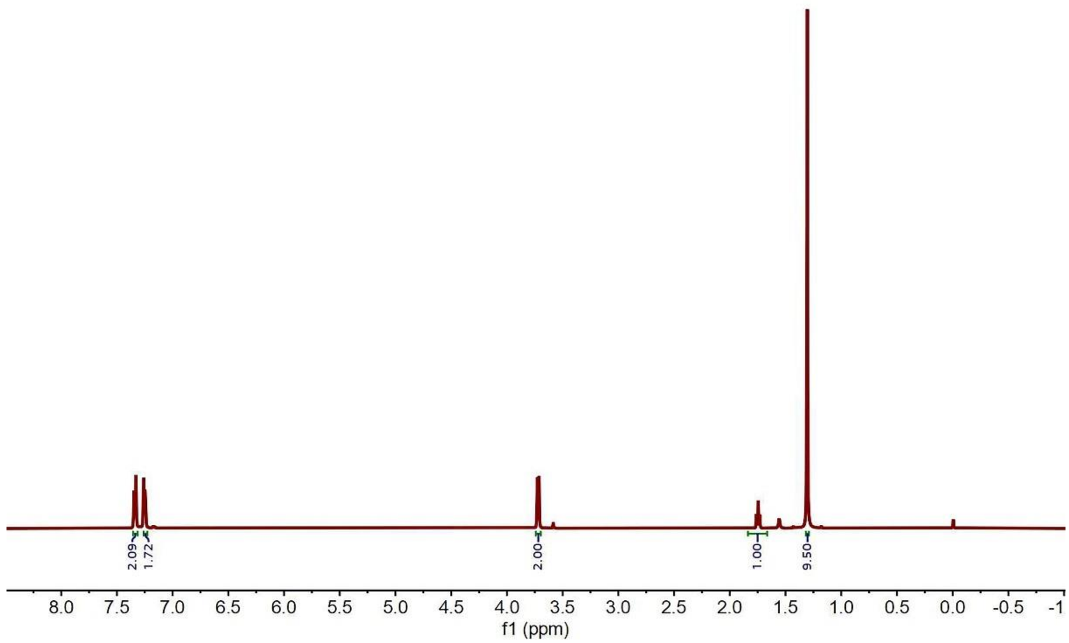

Fig. S31 ^{1}H-NMR spectrum of TBBM in $CDCl_3$.

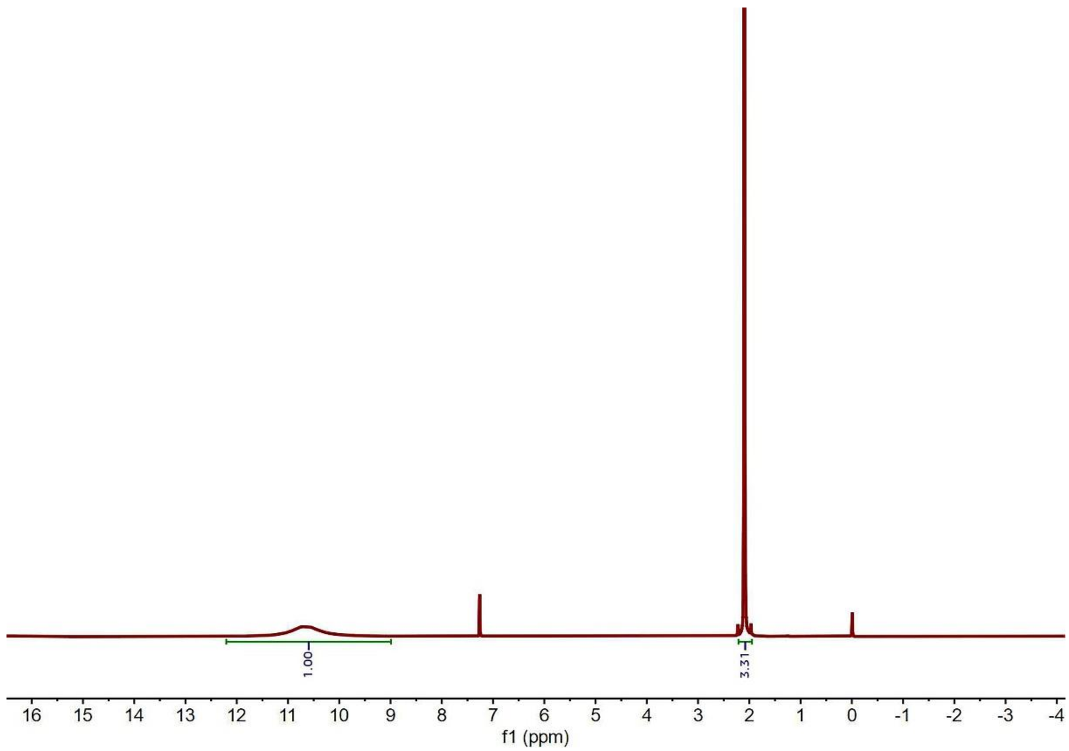

Fig. S32 ^{1}H-NMR spectrum of CH_3COOH in $CDCl_3$.

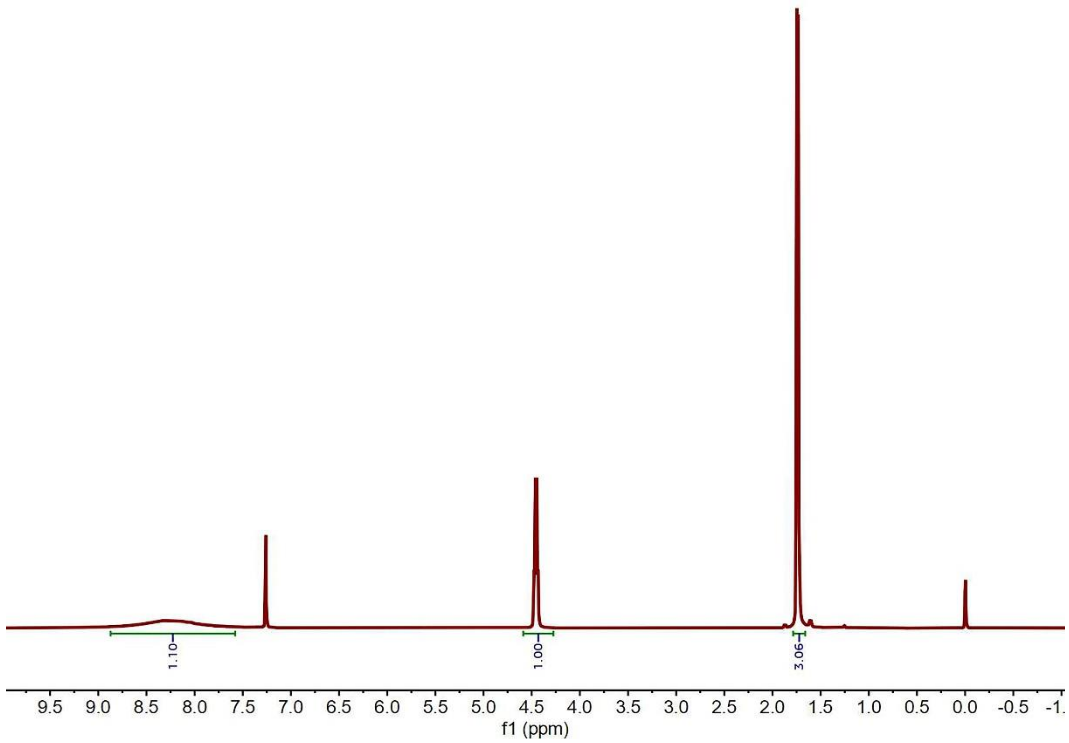

Fig. S33 ^{1}H-NMR spectrum of R-2-chloropropionic acid in $CDCl_3$.

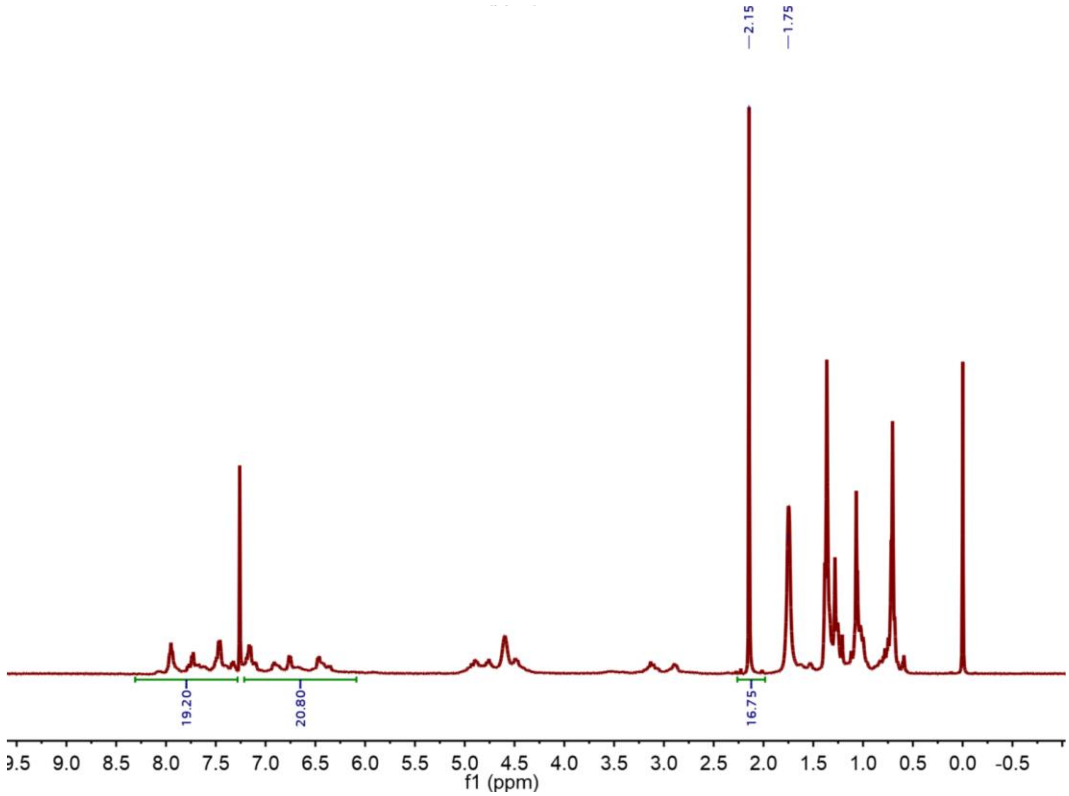

Fig. S34 ^{1}H-NMR spectrum of **Ag_{40}** nanocluster reacted with R-2-chloropropionic acid in $CDCl_3$. The molar ratio of **Ag_{40}** to R-2-chloropropionic acid is set as 1:12 (corresponding to the maximum value of CD spectra). The 2.15 ppm signal belongs to the methyl group of free CH_3COOH replaced by R-2-chloropropionic acid. The 1.75 ppm signal is contributed by two substances: i) the methyl group of R-2-chloropropionic acid, and ii) the methyl group of CH_3COOH on **Ag_{40}** nanocluster.

Calculation method of ligand exchange quantity: 2.15 ppm is the free acetic acid molecule after exchange (Fig. S32). Taking the number of hydrogen (40 H) in the benzene ring region of the cluster as the standard, and integrating at 2.15 ppm, the result is 16.75 H. Thus, the average amount of CH_3COOH exchanged is 16.75/3 = 5.6.

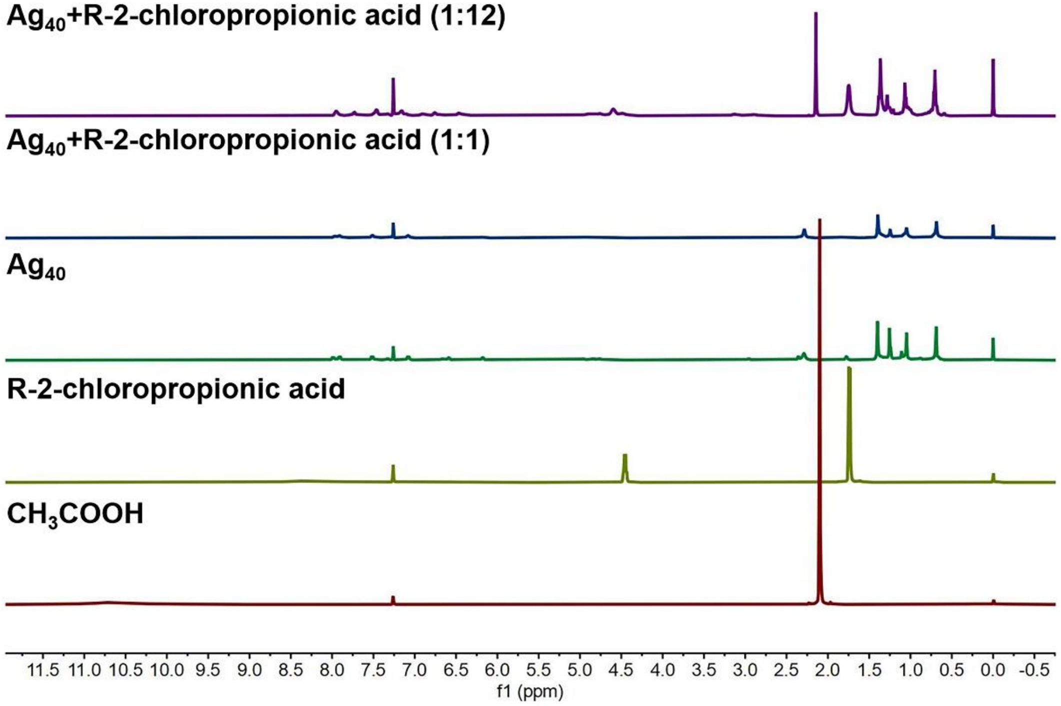

Fig. S35 ^{1}H-NMR spectra comparison of **Ag_{40}** nanocluster reacted with R-2-chloropropionic acid.

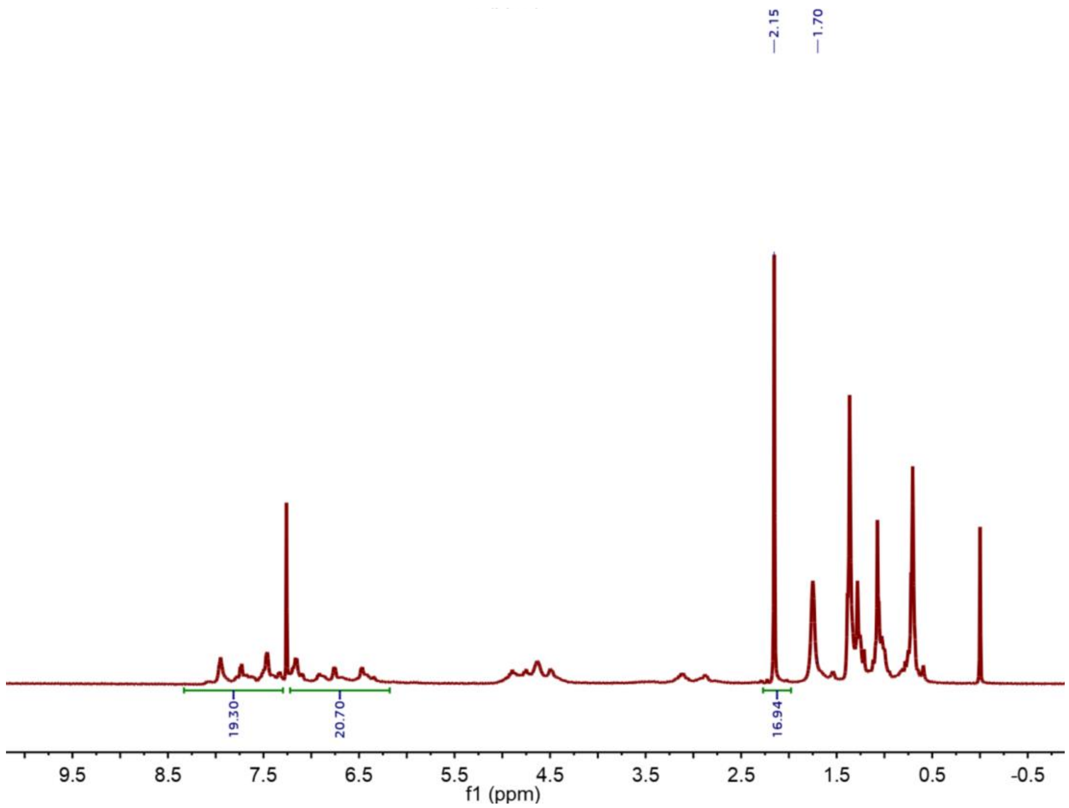

Fig. S36 ^{1}H-NMR spectrum of **Ag_{40}** nanocluster reacted with S-2-chloropropionic acid in $CDCl_3$. The molar ratio of **Ag_{40}** to S-2-chloropropionic acid is set as 1:12. The 2.15 ppm signal belongs to the methyl group of free CH_3COOH replaced by S-2-chloropropionic acid. The 1.70 ppm sigenal is contributed by two substances: i) the methyl group of S-2-chloropropionic acid, and ii) the methyl group of CH_3COOH in **Ag_{40}** nanocluster.

According to the same calculation method as that of R-2-chloropropionic acid, the obtained ligand exchange number is 5.6 (statistical results).

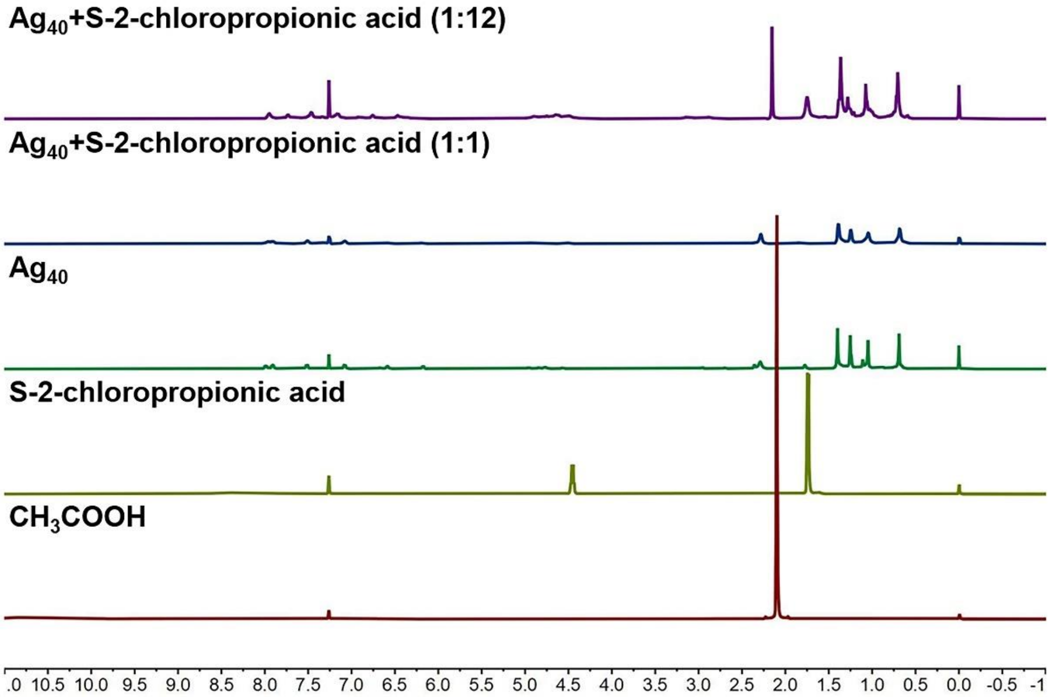

Fig. S37 ^{1}H-NMR spectra comparison of **Ag_{40}** nanocluster reacted with S-2-chloropropionic acid.

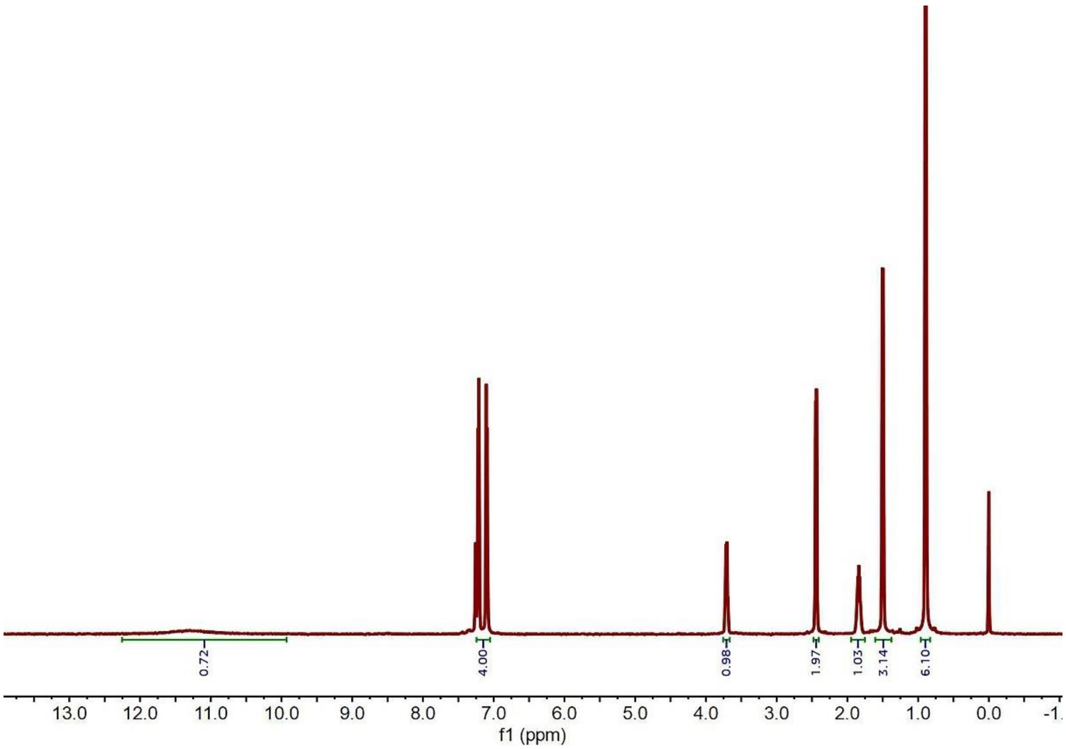

Fig. S38 ^{1}H-NMR spectrum of R-ibuprofen in $CDCl_3$.

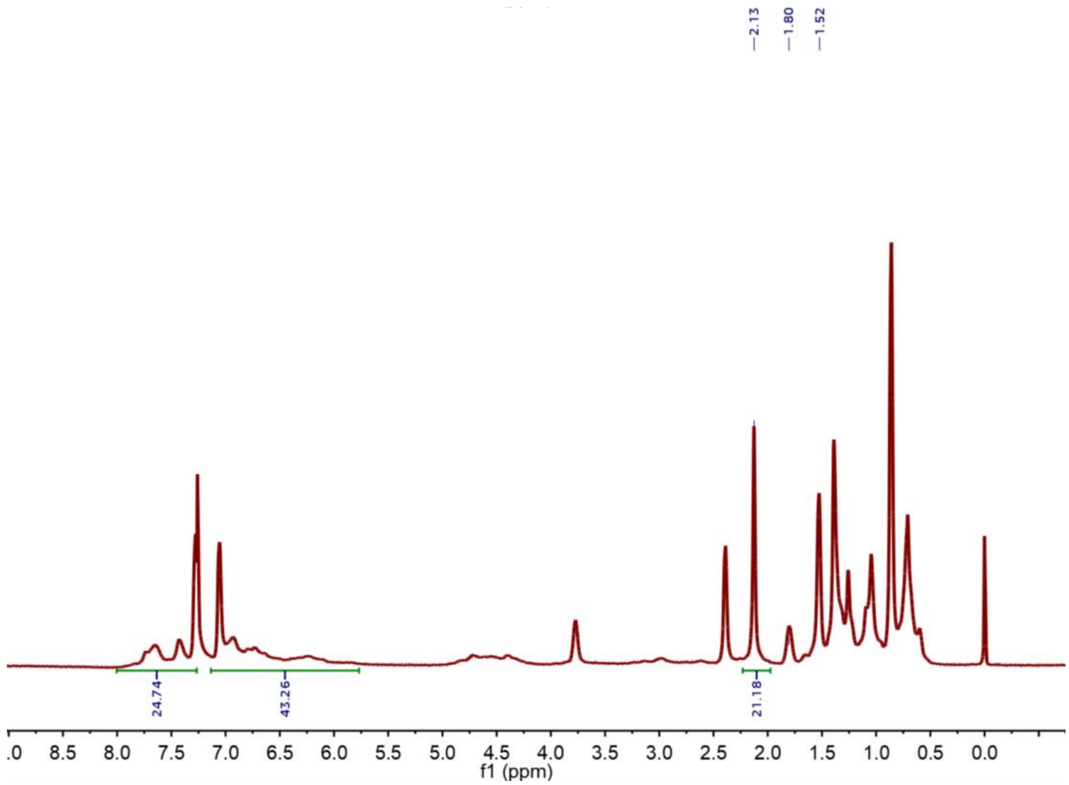

Fig. S39 ^{1}H-NMR spectrum of **Ag_{40}** nanocluster reacted with R-ibuprofen in $CDCl_3$. The molar ratio of **Ag_{40}** to R-ibuprofen is set as 1:7. The 2.13 ppm signal belongs to the methyl group of free CH_3COOH replaced by R-ibuprofen. The 1.80 ppm signal belongs to the methyl group of CH_3COOH in **Ag_{40}** nanocluster. The 1.52 ppm signal belongs to the methyl group (bonded to α-carbon) of R-ibuprofen.

The calculation method is the same as above. It should be noted that since ibuprofen itself has H in the benzene ring region, the total number of hydrogens in all benzene ring regions is set to 68 (40 + 7 x 4 = 68). The amount of CH_3COOH exchanged is 21.18/3 = 7.07. This value represents that the ability of ibuprofen binding to clusters is much higher than that of acetic acid.

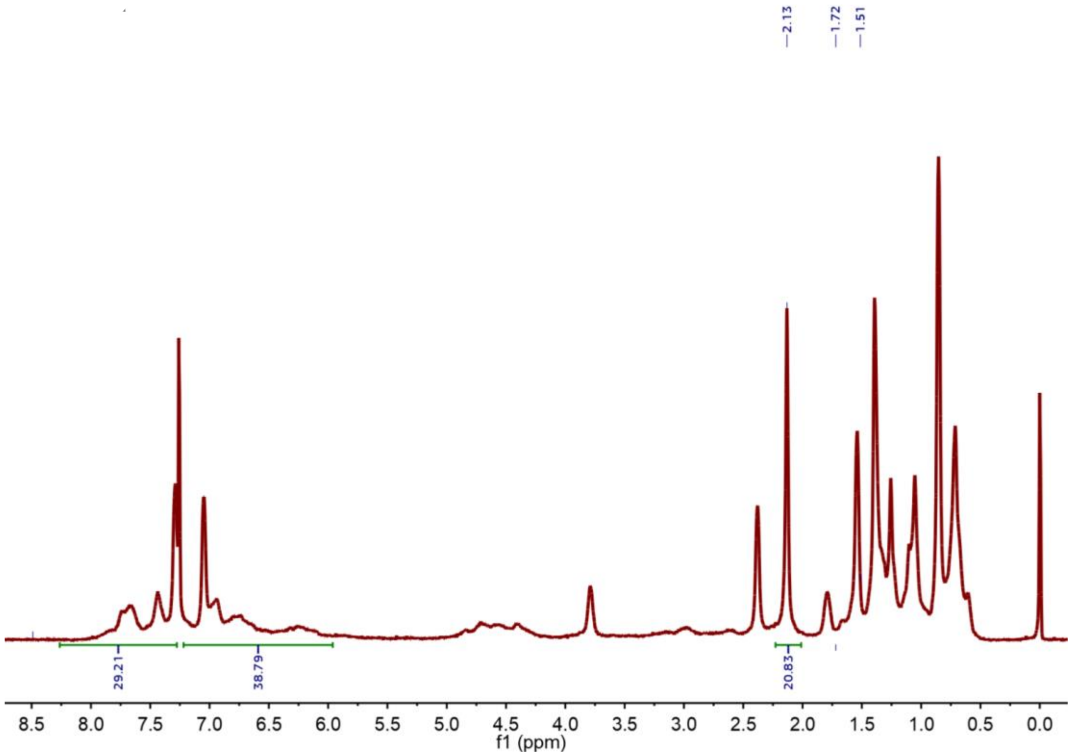

Fig. S40 ^{1}H-NMR spectrum of $\mathbf{Ag_{40}}$ nanocluster reacted with S-ibuprofen in $CDCl_3$. The molar ratio of $\mathbf{Ag_{40}}$ to S-ibuprofen are set as 1:7. The 2.13 ppm signal belongs to the methyl group of free CH_3COOH replaced by S-ibuprofen. The 1.72 ppm signal belongs to the methyl group of CH_3COOH in $\mathbf{Ag_{40}}$ nanocluster. The 1.51 ppm sigenal ibelongs to the methyl group (bonded to α-carbon) of S-ibuprofen.

The calculation method is the same as above. It should be noted that since ibuprofen itself has H in the benzene ring region, the total number of hydrogens in all benzene ring regions is set to 68 (40 + 7 x 4 = 68). The amount of CH_3COOH exchanged is 20.83/3 = 6.94. This value represents that the ability of ibuprofen to bind to clusters is much higher than that of acetic acid.

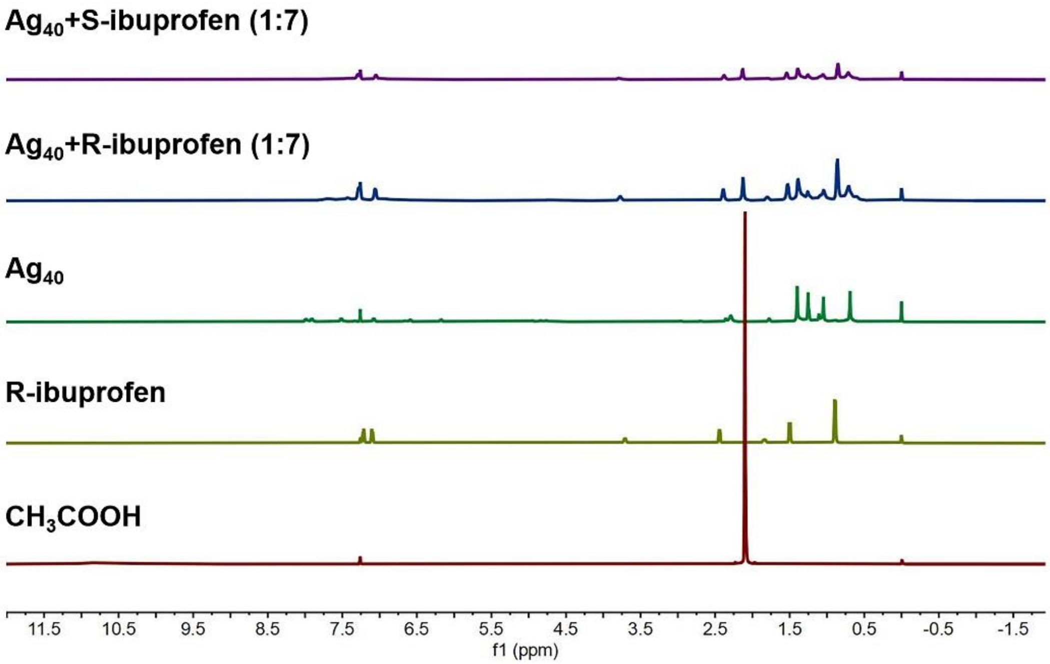

Fig. S41 ^{1}H-NMR spectra comparison of **Ag_{40}** nanocluster reacted with R/S-ibuprofen.

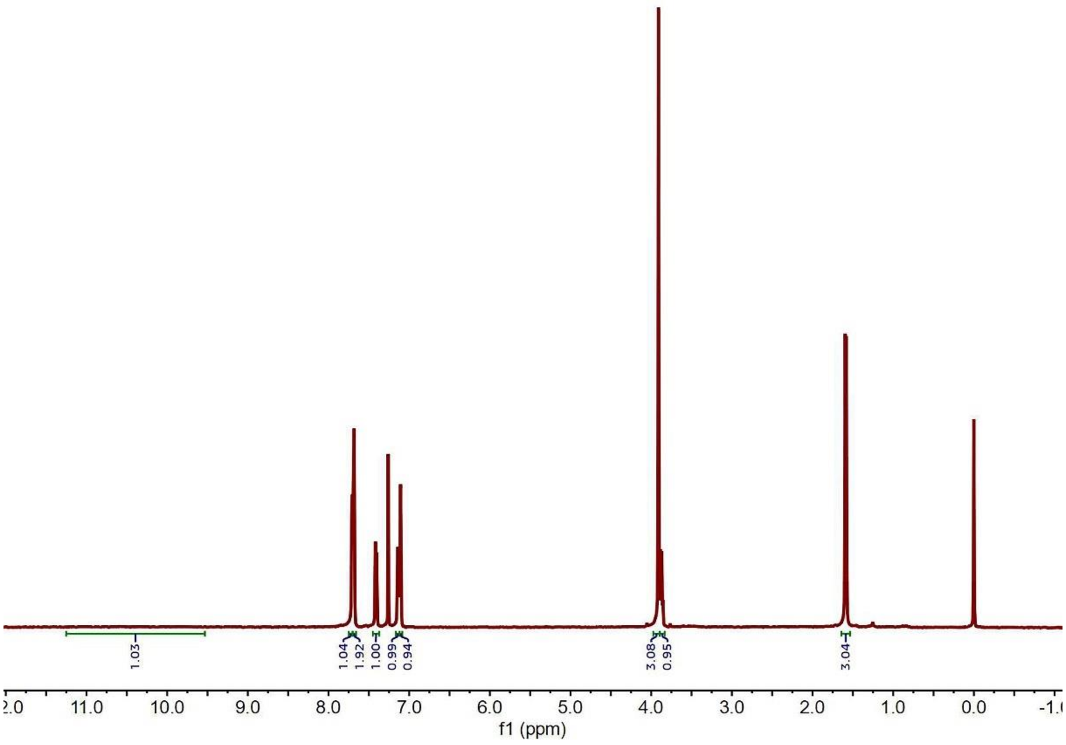

Fig. S42 ^{1}H-NMR spectrum of R-naproxen in $CDCl_3$.

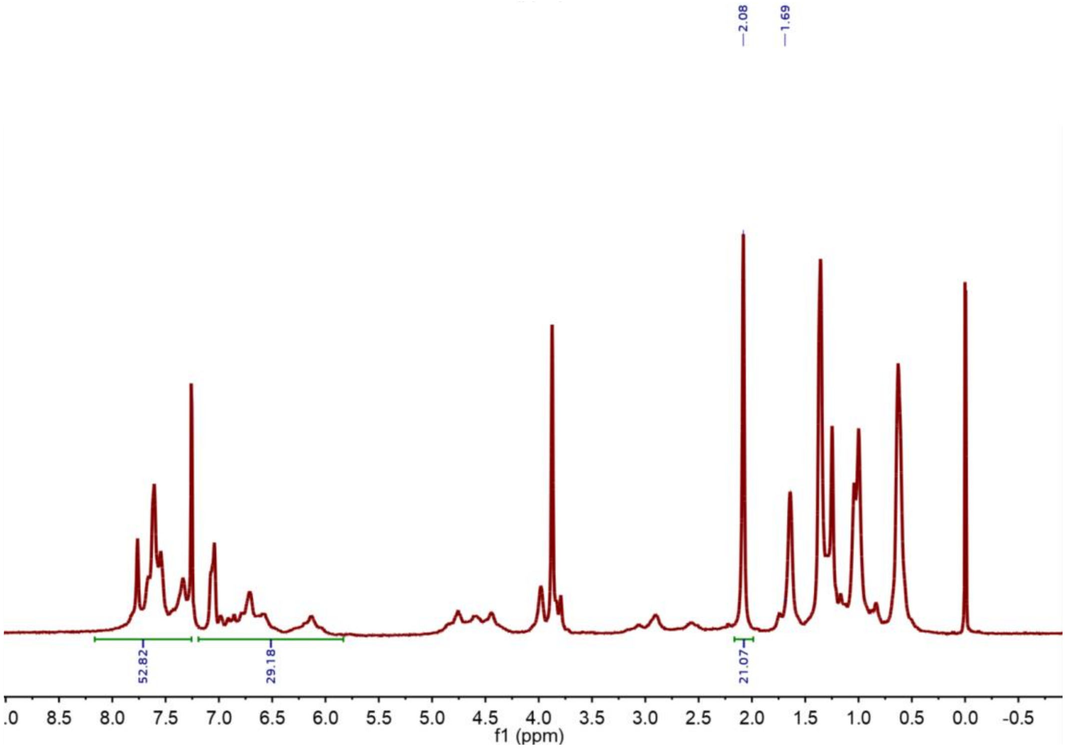

Fig. S43 ^{1}H-NMR spectrum of **Ag_{40}** nanocluster reacted with R-naproxen in $CDCl_3$. The molar ratio of **Ag_{40}** to R-naproxen is set as 1:7. The 2.08 ppm signal belongs to the methyl group of free CH_3COOH replaced by R-naproxen. The 1.69 ppm signal is contributed by two substances: i) the methyl group of R- R-naproxen, and ii) the methyl group of CH_3COOH on **Ag_{40}** nanocluster.

The calculation process is as follows:

The calculation method is the same as above. It should be noted that since naproxen itself has H in the benzene ring region, the total number of hydrogens in all benzene ring regions is set to 68 (40 + 7 x 6 = 82). The amount of CH_3COOH exchanged is 21.07/3 = 7.02. This value represents that the ability of naproxen binding to clusters is much higher than that of acetic acid.

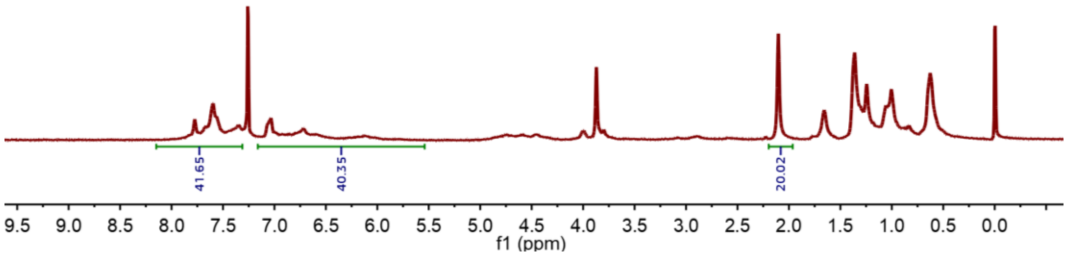

Fig. S44 ^{1}H-NMR spectrum of **Ag$_{40}$** nanocluster reacted with S-naproxen in $CDCl_3$. The molar ratio of **Ag$_{40}$** to S-naproxen is set as 1:7. The 2.08 ppm signal belongs to the methyl group of free CH_3COOH replaced by S-naproxen. The 1.69 ppm signal is contributed by two substances: i) the methyl group of S-naproxen; ii) the methyl group of CH_3COOH on **Ag$_{40}$** nanocluster.

The calculation method is the same as above. It should be noted that since naproxen itself has H in the benzene ring region, the total number of hydrogens in all benzene ring regions is set to 68 (40 + 7 x 6 = 82). The amount of CH_3COOH exchanged is 20.02/3 = 6.7. This value represents that the ability of naproxen to bind to clusters is much higher than that of acetic acid.

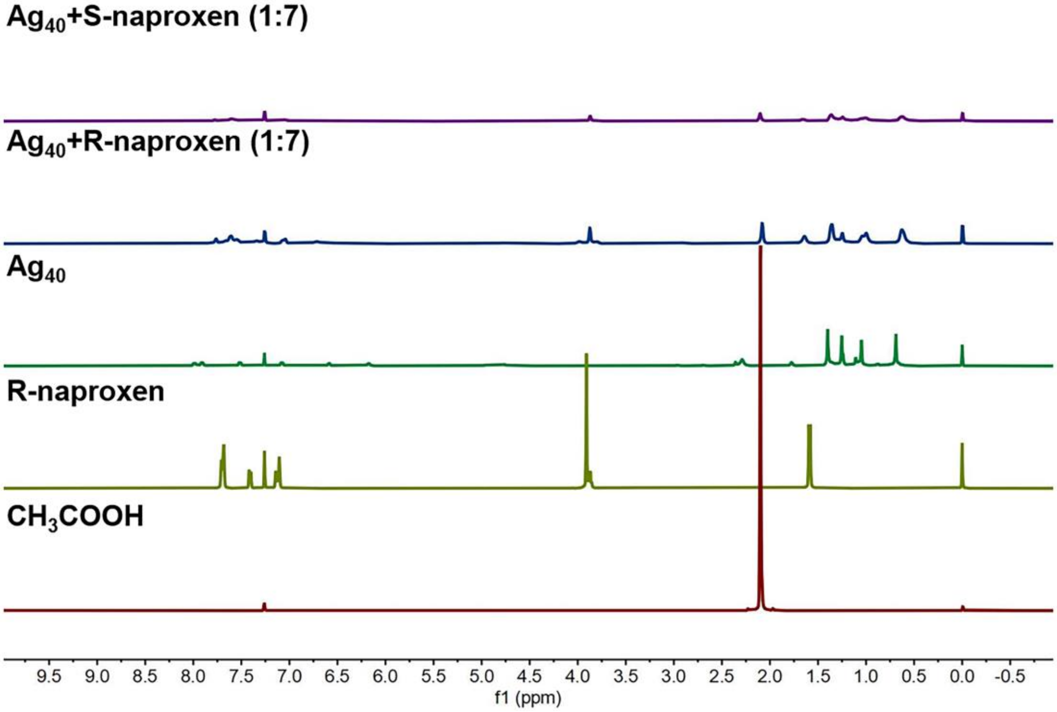

Fig. S45 ^{1}H-NMR spectra comparison of **Ag_{40}** nanocluster reacted with R/S-naproxen.

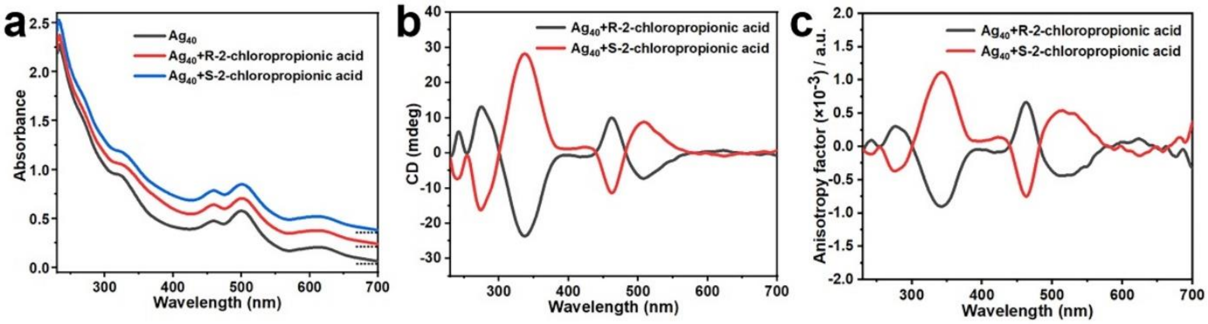

Fig. S46 (**a**) UV-Vis spectra of **Ag_{40}**, **Ag_{40}** (R-2-chloropropionic acid) and **Ag_{40}** (S-2-chloropropionic acid). (**b**) CD spectra of **Ag_{40}** (R-2-chloropropionic acid) and **Ag_{40}** (S-2-chloropropionic acid). (**c**) g-factor of **Ag_{40}** (R-2-chloropropionic acid) and **Ag_{40}** (S-2-chloropropionic acid).

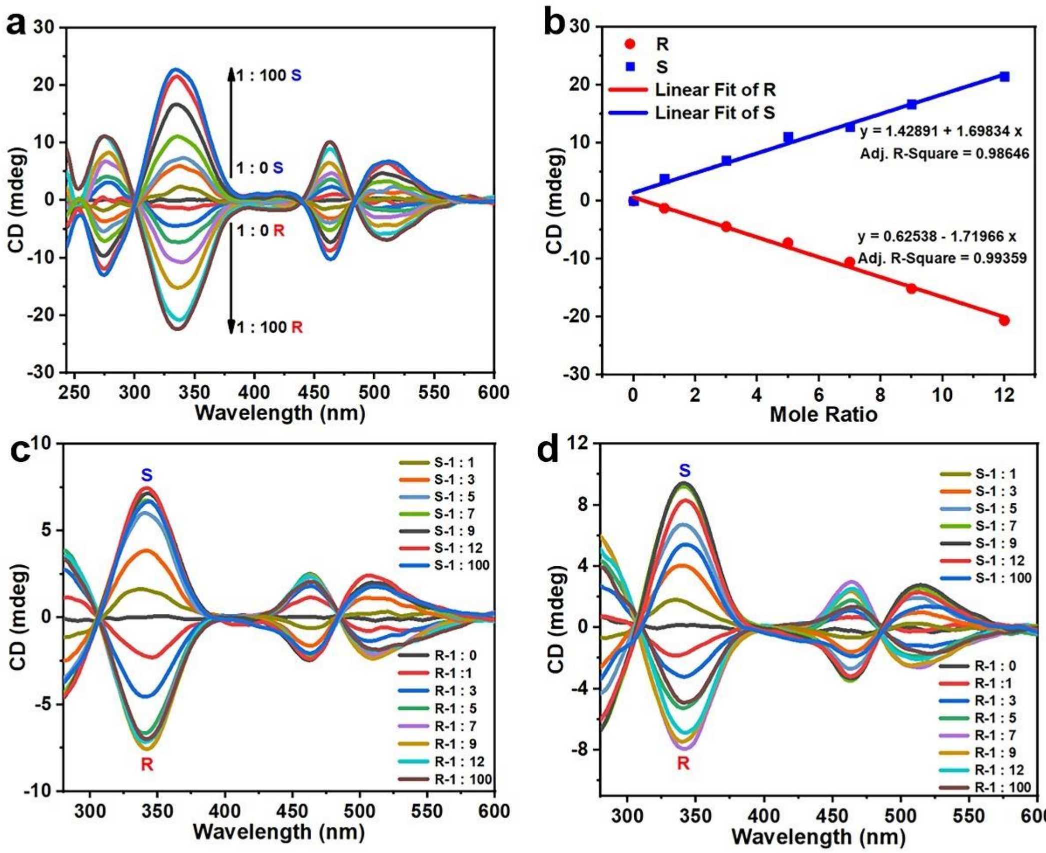

Fig. S47 Application of **Ag_{40}** in chiral sensing. (**a**) CD spectra of **Ag_{40}** with varying concentration of R/S-2-chloropropionic acid, the molar ratios of **Ag_{40}** to R/S-2-chloropropionic acid are set as 1:1, 1:3, 1:5, 1:7, 1:9, 1:12 and 1:100 in CH_2Cl_2. (**b**) The fitted curve by plotting CD readings at 337 nm in Fig. S47a against the mole ratios of chiral 2-chloropropionic acid. (**c**) CD spectra of **Ag_{40}** with varying concentration of R/S-ibuprofen, the molar ratios of **Ag_{40}** to R/S-ibuprofen are set as 1:1, 1:3, 1:5, 1:7, 1:9, 1:12 and 1:100 in CH_2Cl_2. (**d**) CD spectra of **Ag_{40}** with varying concentration of R/S-naproxen, the molar ratios of **Ag_{40}** to R/S-naproxen are set as 1:1, 1:3, 1:5, 1:7, 1:9, 1:12 and 1:100 in CH_2Cl_2. The concentration of **Ag_{40}** is 5.8×10^{-6} mol/L.

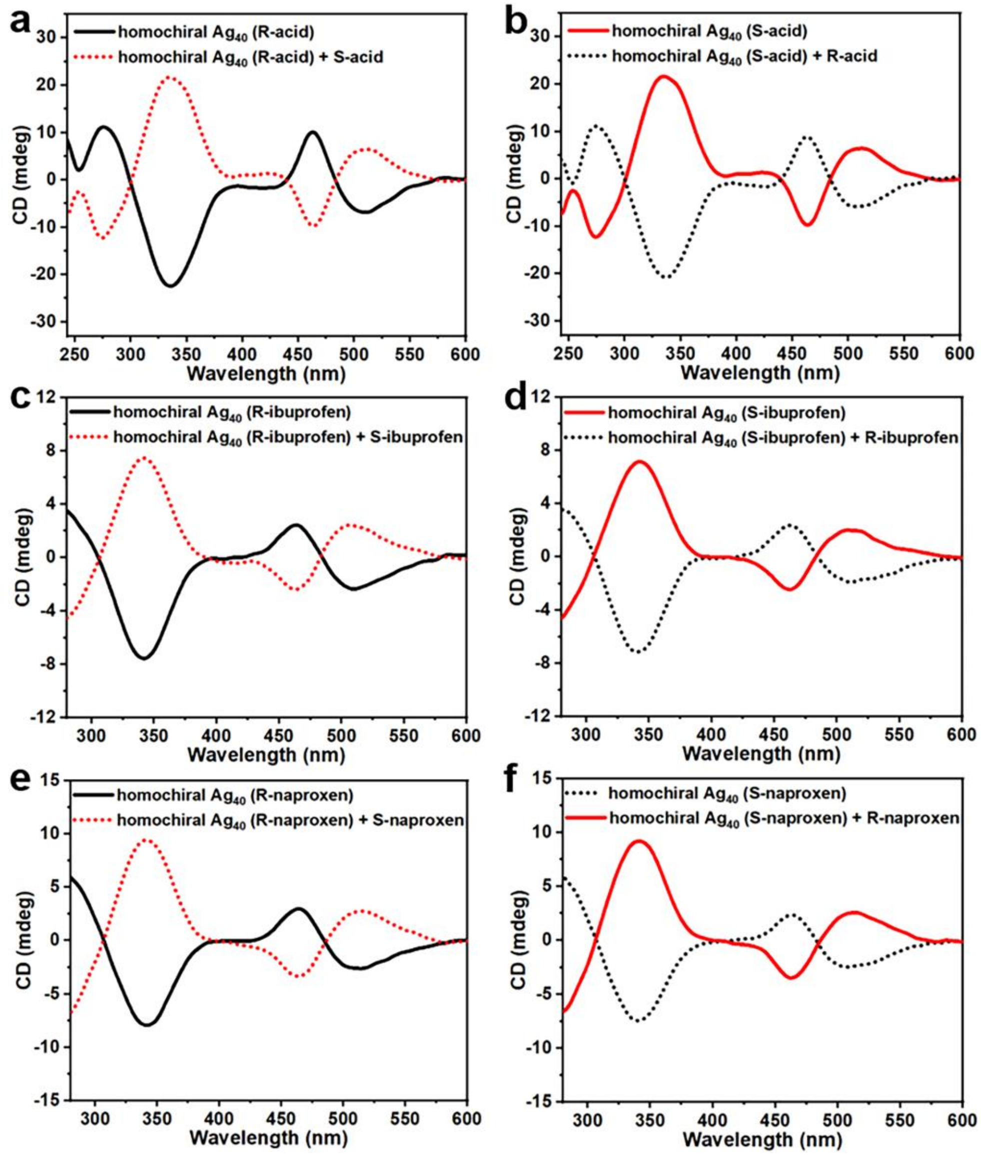

Fig. S48 The CD spectra of homochiral **Ag_{40}** (chiral acids) before and after reaction with the chiral acids of opposite chirality. (**a**) The CD spectra of homochiral **Ag_{40}** (R-2-chloropropionic acid) with S-2-chloropropionic acid; R/S-2-chloropropionic acid is abbreviated R/S-acid. (**b**) The CD spectra of homochiral **Ag_{40}** (S-2-chloropropionic acid) with R-2-chloropropionic acid. (**c**) The CD spectra of homochiral **Ag_{40}** (R-ibuprofen) with S-ibuprofen. (**d**) The CD spectra of homochiral **Ag_{40}** (S-ibuprofen) with R-ibuprofen. (**e**) The CD spectra of homochiral **Ag_{40}** (R-naproxen) with S-naproxen. (**f**) The CD spectra of homochiral **Ag_{40}** (S-naproxen) with R-naproxen.

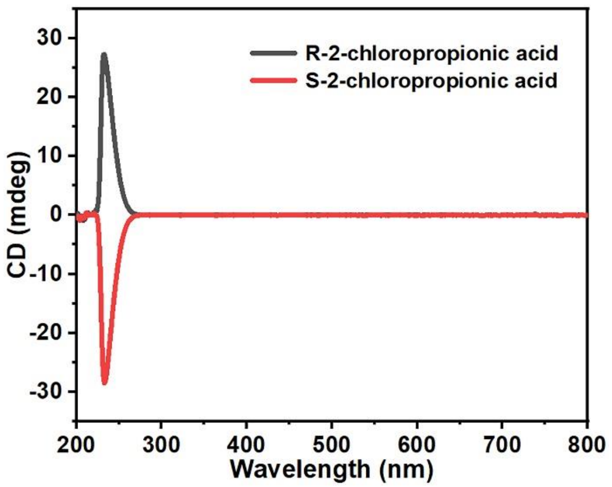

Fig. S49 CD spectra of R/S-2-chloropropionic acid in CH_2Cl_2.

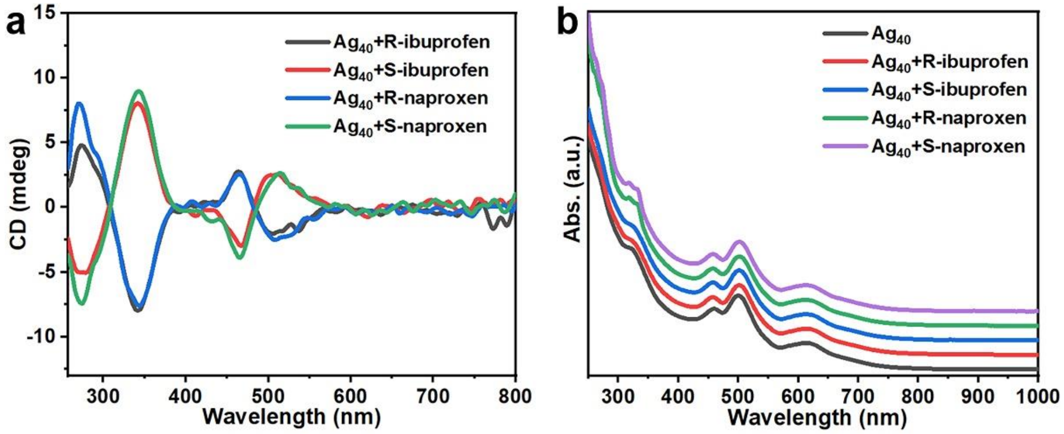

Fig. S50 CD and UV-vis spectra of **Ag_{40}** nanocluster with R/S-ibuprofen and R/S-naproxen. (**a**) CD spectra. (**b**) UV-vis spectra.

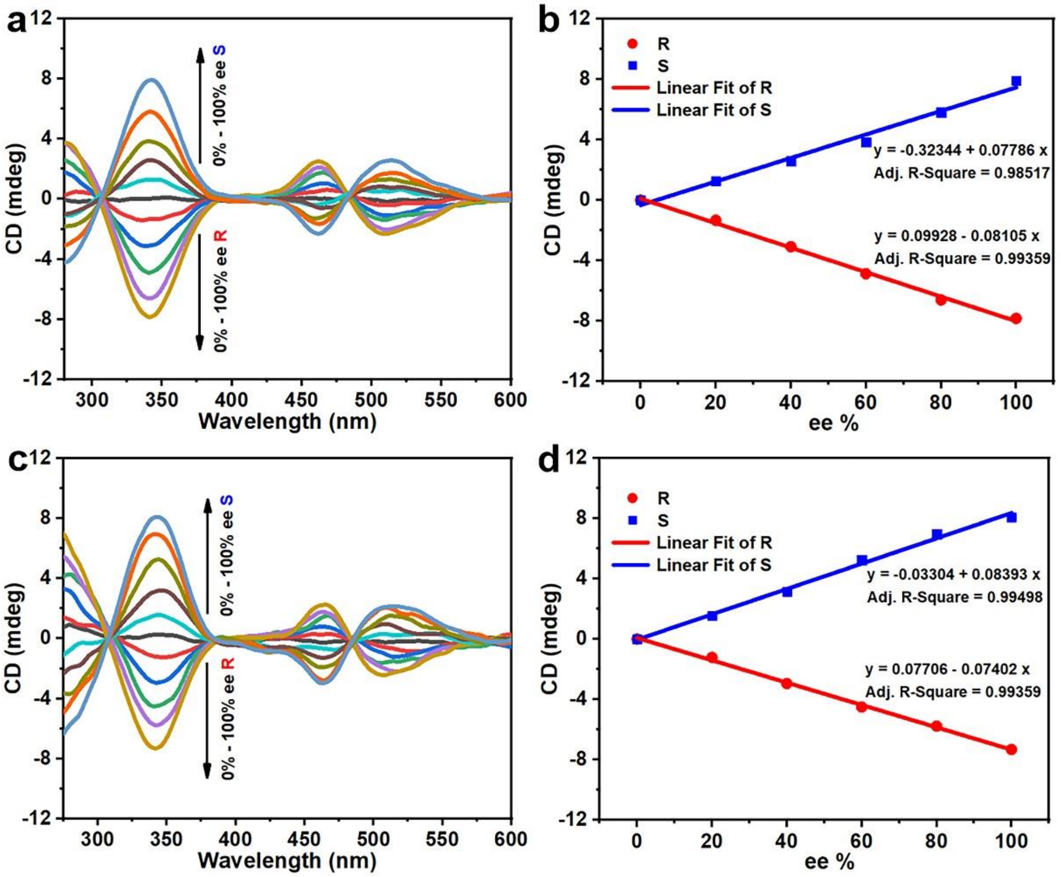

Fig. S51(**a**) CD spectra of the reaction mixture of varied ee value (0, 20, 40, 60, 80, 100%) of R/S-ibuprofen and **Ag**$_{40}$ in CH_2Cl_2. (**b**) The fitted curve by plotting CD readings at 342 nm in Fig. S51a against ee values of chiral ibuprofen. (**c**) CD spectra of the reaction mixture of varied ee value (0, 20, 40, 60, 80, 100%) of R/S-naproxen and **Ag**$_{40}$ in CH_2Cl_2. (d) The fitted curve by plotting CD readings at 342 nm in Fig. S51c against ee values of chiral naproxen. Pure R/S-ibuprofen and R/S-naproxen regarded as ee = 100%.

Table S1. Type and quantity of crystals crystallized.

Type of crystals	Quantity
Total crystal vials	432
Crystal vials of one crystal	42
Crystal vials for *P-1* space group	430
Crystal vials for $P2_12_12_1$ space group	2

Table S2. Crystal data and structure refinement for racemic $Ag_{40}(TBBM)_{20}(CH_3COO)_{12}$ NC.

Empirical formula	$C_{244}H_{335}Ag_{40}O_{24}S_{20}(CH_2Cl_2)_5(MeCN)_3$
Formula weight	8608.10
Temperature/K	293(2)
Crystal system	triclinic
Space group	P-1
a/Å	20.5883(2)
b/Å	21.2117(2)
c/Å	40.8956(3)
α/°	83.7160(10)
β/°	86.9810(10)
γ/°	82.9680(10)
Volume/Å^3	17604.8(3)
Z	2
ρ_{calc}g/cm^3	1.624
μ/mm^{-1}	18.823
F(000)	8382.0
Crystal size/mm^3	0.1 × 0.08 × 0.06
Radiation	Cu Kα (λ = 1.54184)
2Θ range for data collection/°	5.738 to 153.488
Index ranges	$-25 \le h \le 25$, $-25 \le k \le 26$, $-47 \le l \le 50$
Reflections collected	193033
Independent reflections	69132 [R_{int} = 0.0956, R_{sigma} = 0.0888]
Data/restraints/parameters	69132/6215/3145
Goodness-of-fit on F^2	1.072
Final R indexes [I>=2σ (I)]	R_1 = 0.0858, wR_2 = 0.2358
Final R indexes [all data]	R_1 = 0.1037, wR_2 = 0.2509
Largest diff. peak/hole / e Å^{-3}	3.57/-3.99

Table S3. Crystal data and structure refinement for Ag_{40}-R piece 1.

Empirical formula	$C_{244}H_{336}Ag_{40}O_{24}S_{20}(CH_2Cl_2)_{5.95}(MeCN)_1$
Formula weight	8609.11
Temperature/K	170.0
Crystal system	orthorhombic
Space group	$P2_12_12_1$
a/Å	26.0053(9)
b/Å	33.3937(11)
c/Å	34.2186(8)
α/°	90
β/°	90
γ/°	90
Volume/Å^3	29715.9(16)
Z	4
ρ_{calc}g/cm^3	1.924
μ/mm^{-1}	2.757
F(000)	16768.0
Crystal size/mm^3	3 × 0.5 × 0.5
Radiation	Mo Kα (λ = 0.71073)
2Θ range for data collection/°	4.962 to 61.664
Index ranges	$-29 \le h \le 34$, $-43 \le k \le 35$, $-46 \le l \le 44$
Reflections collected	214683
Independent reflections	72628 [R_{int} = 0.0996, R_{sigma} = 0.1331]
Data/restraints/parameters	72628/5207/2742
Goodness-of-fit on F^2	0.979
Final R indexes [I>=2σ (I)]	R_1 = 0.0701, wR_2 = 0.1583
Final R indexes [all data]	R_1 = 0.1200, wR_2 = 0.1781
Largest diff. peak/hole / e Å^{-3}	3.74/-1.38
Flack parameter	0.020(18)

Table S4. Crystal data and structure refinement for Ag_{40}-R piece 2.

Empirical formula	$C_{244}H_{336}Ag_{40}O_{24}S_{20}(CH_2Cl_2)_{5.95}(MeCN)_1$
Formula weight	8609.11
Temperature/K	170
Crystal system	orthorhombic
Space group	$P2_12_12_1$
a/Å	26.1732(13)
b/Å	33.576(2)
c/Å	34.3743(13)
α/°	90
β/°	90
γ/°	90
Volume/Å^3	30208(3)
Z	4
ρ_{calc}g/cm^3	1.893
μ/mm^{-1}	2.712
F(000)	16768.0
Crystal size/mm^3	3 × 0.45 × 0.45
Radiation	Mo Kα (λ = 0.71073)
2Θ range for data collection/°	4.788 to 61.63
Index ranges	$-32 \le h \le 32$, $-40 \le k \le 41$, $-42 \le l \le 48$
Reflections collected	152208
Independent reflections	67553 [R_{int} = 0.1252, R_{sigma} = 0.1909]
Data/restraints/parameters	67553/5176/2742
Goodness-of-fit on F^2	1.012
Final R indexes [I>=2σ (I)]	R_1 = 0.0934, wR_2 = 0.2050
Final R indexes [all data]	R_1 = 0.1615, wR_2 = 0.2336
Largest diff. peak/hole / e Å^{-3}	3.10/-1.52
Flack parameter	0.03(3)

Table S5. Crystal data and structure refinement for Ag_{40}-R piece 3.

Empirical formula	$C_{244}H_{336}Ag_{40}O_{24}S_{20}(CH_2Cl_2)_{5.95}(MeCN)_1$
Formula weight	8609.11
Temperature/K	170(2)
Crystal system	orthorhombic
Space group	$P2_12_12_1$
a/Å	26.0591(9)
b/Å	33.3757(13)
c/Å	34.2869(10)
α/°	90
β/°	90
γ/°	90
Volume/Å^3	29820.7(18)
Z	4
ρ_{calc}g/cm^3	1.918
μ/mm^{-1}	2.748
F(000)	16768.0
Crystal size/mm^3	0.45 × 0.45 × 0.3
Radiation	Mo Kα (λ = 0.71073)
2Θ range for data collection/°	4.838 to 61.498
Index ranges	$-29 \leq h \leq 36$, $-41 \leq k \leq 35$, $-39 \leq l \leq 46$
Reflections collected	205762
Independent reflections	72284 [R_{int} = 0.1297, R_{sigma} = 0.1669]
Data/restraints/parameters	72284/5203/2784
Goodness-of-fit on F^2	0.991
Final R indexes [I>=2σ (I)]	R_1 = 0.0755, wR_2 = 0.1700
Final R indexes [all data]	R_1 = 0.1508, wR_2 = 0.2010
Largest diff. peak/hole / e Å^{-3}	3.55/-1.42
Flack parameter	0.00(3)

Table S6. Crystal data and structure refinement for Ag_{40}-R piece 4.

Empirical formula	$C_{244}H_{336}Ag_{40}O_{24}S_{20}(CH_2Cl_2)_{5.95}(MeCN)_1$
Formula weight	8609.11
Temperature/K	170(2)
Crystal system	orthorhombic
Space group	$P2_12_12_1$
a/Å	26.0950(6)
b/Å	33.4677(9)
c/Å	34.2216(9)
α/°	90
β/°	90
γ/°	90
Volume/Å^3	29887.1(13)
Z	4
ρ_{calc}g/cm^3	1.913
μ/mm^{-1}	2.741
F(000)	16768.0
Crystal size/mm^3	0.35 × 0.25 × 0.1
Radiation	Mo Kα (λ = 0.71073)
2Θ range for data collection/°	4.762 to 61.546
Index ranges	$-33 \leq h \leq 33$, $-47 \leq k \leq 40$, $-47 \leq l \leq 48$
Reflections collected	192665
Independent reflections	71864 [R_{int} = 0.1153, R_{sigma} = 0.1588]
Data/restraints/parameters	71864/5184/2785
Goodness-of-fit on F^2	0.962
Final R indexes [I>=2σ (I)]	R_1 = 0.0689, wR_2 = 0.1523
Final R indexes [all data]	R_1 = 0.1299, wR_2 = 0.1767
Largest diff. peak/hole / e Å^{-3}	2.88/-1.15
Flack parameter	0.02(3)

Table S7. Crystal data and structure refinement for Ag_{40}-R piece 5.

Empirical formula	$C_{244}H_{336}Ag_{40}O_{24}S_{20}(CH_2Cl_2)_{5.95}(MeCN)_1$
Formula weight	8609.11
Temperature/K	170(2)
Crystal system	orthorhombic
Space group	$P2_12_12_1$
a/Å	26.0950(6)
b/Å	33.4677(9)
c/Å	34.2216(9)
α/°	90
β/°	90
γ/°	90
Volume/Å^3	29887.1(13)
Z	4
ρ_{calc}g/cm^3	1.913
μ/mm^{-1}	2.741
F(000)	16768.0
Crystal size/mm^3	0.7 × 0.6 × 0.15
Radiation	Mo Kα (λ = 0.71073)
2Θ range for data collection/°	4.896 to 61.662
Index ranges	$-33 \leq h \leq 32$, $-46 \leq k \leq 42$, $-43 \leq l \leq 42$
Reflections collected	208219
Independent reflections	73005 [R_{int} = 0.0908, R_{sigma} = 0.1073]
Data/restraints/parameters	73005/5191/2837
Goodness-of-fit on F^2	1.003
Final R indexes [I>=2σ (I)]	R_1 = 0.0600, wR_2 = 0.1440
Final R indexes [all data]	R_1 = 0.1001, wR_2 = 0.1639
Largest diff. peak/hole / e Å^{-3}	3.82/-1.40
Flack parameter	0.017(16)

Table S8. Crystal data and structure refinement for Ag_{40}-L.

Empirical formula	$C_{244}H_{336}Ag_{40}O_{24}S_{20}(CH_2Cl_2)_{1.81}(MeCN)_{2.27}$
Formula weight	8609.11
Temperature/K	170(2)
Crystal system	orthorhombic
Space group	$P2_12_12_1$
a/Å	26.0676(5)
b/Å	33.3838(7)
c/Å	34.1255(9)
α/°	90
β/°	90
γ/°	90
Volume/Å^3	29697.2(12)
Z	4
ρ_{calc}g/cm^3	1.926
μ/mm^{-1}	2.759
F(000)	16768.0
Crystal size/mm^3	0.15 × 0.15 × 0.08
Radiation	Mo Kα (λ = 0.71073)
2Θ range for data collection/°	4.88 to 61.53
Index ranges	-31 ≤ h ≤ 33, -43 ≤ k ≤ 43, -38 ≤ l ≤ 43
Reflections collected	216997
Independent reflections	72013 [R_{int} = 0.0758, R_{sigma} = 0.0960]
Data/restraints/parameters	72013/5241/2840
Goodness-of-fit on F^2	0.997
Final R indexes [I>=2σ (I)]	R_1 = 0.0564, wR_2 = 0.1320
Final R indexes [all data]	R_1 = 0.0936, wR_2 = 0.1486
Largest diff. peak/hole / e Å^{-3}	2.88/-0.85
Flack parameter	-0.018(15)

References

1. O. V. Dolomanov, L. J. Bourhis, R. J. Gildea, J. A. K. Howard, H. Puschmann. Olex2: A complete structure solution, refinement and analysis program. *J. Appl. Crystallogr.* **2009,** *42*, 339-341.

2. G. M. Sheldrick. Shelxt - integrated space-group and crystal-structure determination. *Acta Crystallogr. A Found. Adv.* **2015,** *71*, 3-8.
